# The Brescia Internationally Validated European Guidelines on Minimally Invasive Pancreatic Surgery (EGUMIPS)

**DOI:** 10.1097/SLA.0000000000006006

**Published:** 2023-07-14

**Authors:** Mohammad Abu Hilal, Tess M.E. van Ramshorst, Ugo Boggi, Safi Dokmak, Bjørn Edwin, Tobias Keck, Igor Khatkov, Jawad Ahmad, Hani Al Saati, Adnan Alseidi, Juan S. Azagra, Bergthor Björnsson, Fatih M. Can, Mathieu D’Hondt, Mikhail Efanov, Francisco Espin Alvarez, Alessandro Esposito, Giovanni Ferrari, Bas Groot Koerkamp, Andrew A. Gumbs, Melissa E. Hogg, Cristiano G.S. Huscher, Benedetto Ielpo, Arpad Ivanecz, Jin-Young Jang, Rong Liu, Misha D.P. Luyer, Krishna Menon, Masafumi Nakamura, Tullio Piardi, Olivier Saint-Marc, Steve White, Yoo-Seok Yoon, Alessandro Zerbi, Claudio Bassi, Frederik Berrevoet, Carlos Chan, Felipe J. Coimbra, Kevin C.P. Conlon, Andrew Cook, Christos Dervenis, Massimo Falconi, Clarissa Ferrari, Isabella Frigerio, Giuseppe K. Fusai, Michelle L. De Oliveira, Antonio D. Pinna, John N. Primrose, Alain Sauvanet, Alejandro Serrablo, Sameer Smadi, Ali Badran, Magomet Baychorov, Elisa Bannone, Eduard A. van Bodegraven, Anouk M.L.H. Emmen, Alessandro Giani, Nine de Graaf, Jony van Hilst, Leia R. Jones, Giovanni B. Levi Sandri, Alessandra Pulvirenti, Marco Ramera, Niki Rashidian, Mushegh A. Sahakyan, Bas A. Uijterwijk, Pietro Zampedri, Maurice J.W. Zwart, Sergio Alfieri, Stefano Berti, Giovanni Butturini, Fabrizio Di Benedetto, Giuseppe M. Ettorre, Felice Giuliante, Elio Jovine, Riccardo Memeo, Nazario Portolani, Andrea Ruzzenente, Roberto Salvia, Ajith K. Siriwardena, Marc G. Besselink, Horacio J. Asbun

**Affiliations:** * Department of General Surgery, Istituto Ospedaliero Fondazione Poliambulanza, Brescia, Italy; † Amsterdam UMC, location University of Amsterdam, Department of Surgery, Amsterdam, the Netherlands; ‡ Cancer Center Amsterdam, the Netherlands; § Division of General and Transplant Surgery, University of Pisa, Pisa, Italy; ∥ Department of HPB surgery and liver transplantation, AP-HP, Beaujon Hospital, DMU DIGEST, Clichy, France; ¶ The Intervention Centre, Department of Hepato-Pancreato-Biliary Surgery, Oslo University Hospital and Institute of Medicine, Oslo University, Oslo, Norway; # Clinic for Surgery, University of Schleswig-Holstein Campus Lübeck, Lübeck, Germany; ** Department of Surgery, Moscow Clinical Scientific Center, Moscow, Russia; †† Department of Surgery, University Hospitals Coventry and Warwickshire, West Midlands, UK; ‡‡ Department of Surgery, Salmanyia Medical Complex, Manama, Bahrain; §§ Department of Surgery, University of California - San Francisco, San Francisco, California; ∥∥ Department of General & Minimally Invasive Surgery (Laparoscopy & Robotic), Centre Hospitalier de Luxembourg, Luxembourg, Luxembourg; ¶¶ Department of Surgery in Linköping and Department of Biomedical and Clinical Sciences, Linköping University, Linköping, Sweden; ## Department of General Surgery, Bayindir Sogutozu Hospital, Ankara, Turkey; *** Department of Digestive and Hepatobiliary/Pancreatic Surgery, Groeninge Hospital, Kortrijk, Belgium; ††† Department of Hepato-Pancreato-Biliary Surgery, Hospital Germans Trias i Pujol, Barcelona, Spain; ‡‡‡ Department of Surgery, Pancreas Institute, Verona University Hospital, Verona, Italy; §§§ Division of Minimally invasive Surgical Oncology, ASST Grande Ospedale Metropolitano Niguarda, Milan, Italy; ∥∥∥ Department of Surgery, Erasmus MC Cancer Institute, Rotterdam, Netherlands; ¶¶¶ Department of Visceral Surgery, Centre Hospitalier Intercommunal de Poissy/Saint-Germain-en-Laye, Poissy, France; ### Department of Surgery, Northshore University HealthSystem, Chicago, Illinois; **** Department of Oncologic Surgery, Casa di Cura Cobellis, Vallo Della Lucania, Salerno, Italy; †††† Hepato-Biliary and Pancreatic Surgery Unit, Hospital del Mar, Barcelona, Spain; ‡‡‡‡ Department of Abdominal and General Surgery, University Medical Center Maribor, Maribor, Slovenia; §§§§ Departments of Surgery and Cancer Research Institute, Seoul National University College of Medicine, Seoul, Korea; ∥∥∥∥ Faculty of Hepatopancreatobiliary Surgery, The First Medical Center of Chinese People’s Liberation Army (PLA) General Hospital, Beijing, China; ¶¶¶¶ Department of Surgery, Catharina Hospital, Eindhoven, the Netherlands; #### Institute of Liver Studies, King’s College Hospital, London, UK; ***** Department of Surgery and Oncology, Graduate School of Medical Sciences, Kyushu University, Fukuoka, Japan; ††††† Department of Hepatobiliary, Pancreatic, and Digestive Oncological Surgery, Robert Debré University Hospital, Reims, France; ‡‡‡‡‡ Department of Surgery, Centre Hospitalier Regional Orleans, Orleans, France; §§§§§ Department of HPB and Liver Transplant Surgery, The Freeman Hospital, Newcastle, United Kingdom; ∥∥∥∥∥ Department of Surgery, Seoul National University Bundang Hospital, Seoul, Korea; ¶¶¶¶¶ Department of Biomedical Sciences, Humanitas University, Pieve Emanuele, MI, Italy and IRCCS Humanitas Research Hospital, Rozzano, Italy; ##### Department of General and HPB Surgery and Liver Transplantation, Ghent University Hospital, Ghent, Belgium; ****** Surgery Department, Instituto Nacional de Ciencias Medicas y Nutricion Salvador Zubiran, Mexico, Mexico; †††††† Department of Abdominal Surgery, AC Camargo Cancer Center, São Paulo, Brazil; ‡‡‡‡‡‡ Department of Surgery, St. Vincent’s University Hospital Dublin, Ireland; §§§§§§ Southampton Clinical Trials Unit, University Hospital Southampton, Southampton, UK; ∥∥∥∥∥∥ Department of Surgery, AGIA OLGA Hospital, Athens, Greece; ¶¶¶¶¶¶ Pancreas Translational & Clinical Research Center, San Raffaele IRCCS, Università Vita-Salute, Milan, Italy; ###### Unit of Statistics, Istituto Ospedaliero Fondazione Poliambulanza, Brescia, Italy; ******* Department of HPB Surgery, Pederzoli Hospital, Peschiera del Garda, Italy; ††††††† HPB & Liver Transplant Unit, Royal Free London, London, UK; ‡‡‡‡‡‡‡ Department of Surgery and Transplantation, Swiss HPB Center, University Hospital Zurich, Switzerland; §§§§§§§ Department of Abdominal and Transplant Surgery, Cleveland Clinic Florida, Weston, Florida; ∥∥∥∥∥∥∥ Department of Surgery, University Hospital Southampton NHS Foundation Trust, Southampton, United Kingdom; ¶¶¶¶¶¶¶ Department of HPB Surgery and Liver Transplantation, Hospital of Beaujon, Paris, France; ####### HPB Surgical Division, Miguel Servet University Hospital, Zaragoza, Spain; ******** Department of Surgery, King Hussein Medical Center, Amman, Jordan; †††††††† Division of General Surgery and Liver Transplantation, S. Camillo Forlanini Hospital, Rome, Italy; ‡‡‡‡‡‡‡‡ The Intervention Centre, Oslo University Hospital, Oslo, Norway; §§§§§§§§ Department of Research & Development, Division of Emergencies and Critical Care, Oslo University Hospital, Oslo, Norway; ∥∥∥∥∥∥∥∥ Department of Surgery N1, Yerevan State Medical University, Yerevan, Armenia; ¶¶¶¶¶¶¶¶ Department of Surgery, Fondazione Policlinico Universitario A. Gemelli IRCCS, Rome, Italy; ######## Department of General Surgery, “Sant’Andrea” Hospital La Spezia, La Spezia, Italy; ********* Hepatopancreatobiliary Surgery and Liver Transplant Unit, University of Modena and Reggio Emilia, Modena, Italy; ††††††††† Alma Mater Studiorum, University of Bologna, AOU Sant’Orsola Malpighi, IRCCS at Maggiore Hospital, Bologna, Italy; ‡‡‡‡‡‡‡‡‡ Department of Hepato-Pancreatic-Biliary Surgery, Miulli Hospital, Bari, Italy; §§§§§§§§§ Department of Clinical and Experimental Sciences, Surgical Clinic, University of Brescia, Italy; ∥∥∥∥∥∥∥∥∥ Department of Surgery, General and Hepatobiliary Surgery, University Hospital G.B. Rossi, University and Hospital Trust of Verona, Verona, Italy; ¶¶¶¶¶¶¶¶¶ Hepatobiliary and Pancreatic Surgery Unit, Manchester University NHS FT, Manchester, UK; ######### Hepato-Biliary and Pancreas Surgery, Miami Cancer Institute, Miami, Florida, USA

**Keywords:** evidence-based guidelines, minimally invasive pancreatic surgery, laparoscopic pancreatic surgery, robotic pancreatic surgery

## Abstract

**Objective:**

To develop and update evidence-based and consensus-based guidelines on laparoscopic and robotic pancreatic surgery.

**Summary Background Data:**

Minimally invasive pancreatic surgery (MIPS), including laparoscopic and robotic surgery, is complex and technically demanding. Minimizing the risk for patients requires stringent, evidence-based guidelines. Since the International Miami Guidelines on MIPS in 2019, new developments and key publications have been reported, necessitating an update.

**Methods:**

Evidence-based guidelines on 22 topics in 8 domains were proposed: terminology, indications, patients, procedures, surgical techniques and instrumentation, assessment tools, implementation and training, and artificial intelligence. The Brescia Internationally Validated European Guidelines on Minimally Invasive Pancreatic Surgery (EGUMIPS, September 2022) used the Scottish Intercollegiate Guidelines Network (SIGN) methodology to assess the evidence and develop guideline recommendations, the Delphi method to establish consensus on the recommendations among the Expert Committee, and the AGREE II-GRS tool for guideline quality assessment and external validation by a Validation Committee.

**Results:**

Overall, 27 European experts, 6 international experts, 22 international Validation Committee members, 11 Jury Committee members, 18 Research Committee members, and 121 registered attendees of the 2-day meeting were involved in the development and validation of the guidelines. In total, 98 recommendations were developed, including 33 on laparoscopic, 34 on robotic, and 31 on general MIPS, covering 22 topics in 8 domains. Out of 98 recommendations, 97 reached at least 80% consensus among the experts and congress attendees, and all recommendations were externally validated by the Validation Committee.

**Conclusions:**

The EGUMIPS evidence-based guidelines on laparoscopic and robotic MIPS can be applied in current clinical practice to provide guidance to patients, surgeons, policy-makers, and medical societies.

Minimally invasive pancreatic surgery (MIPS) has become increasingly popular in the last decades and is now considered an important part of current pancreatic surgery practice. This evolution has been supported by large literature series from expert centers,^1,2^ training programs,^3–7^ and promising results of the majority of randomized controlled trials.^8–13^ Nevertheless, MIPS—including laparoscopic and robotic surgery—is complex and technically demanding surgery associated with a long learning curve and high postoperative morbidity rates, and as such, it calls for stringent implementation of evidence-based guidelines to minimize patient harm. For this reason, in 2019, the Miami International Evidence-based Guidelines on Minimally Invasive Pancreas Resection (IG-MIPR) were established,^14^ aiming to guide the safe adoption of MIPS covering many relevant topics such as indications, patient selection, learning curves, training, and center volumes. However, since then, new literature has become available, and a significant expansion in robotic procedures has been seen. The IG-MIPR mainly included general guidelines on MIPS without distinguishing between laparoscopic and robotic surgery. The guidelines concluded that laparoscopic, robotic, and open pancreas resection each have their role, and future research should focus on the utility and (technical) advantages of each approach. Several studies have recently supported this, as different outcomes after laparoscopic and robotic surgery have been reported.^1,10,15–18^

Therefore, an update of the previous guidelines, which includes separate guidelines for laparoscopic and robotic pancreatic surgery, is needed. The First Internationally Validated European Guidelines on Minimally Invasive Pancreatic Surgery (EGUMIPS) finally achieved consensus in Brescia (September 2022), aiming to provide new terminology on surgical approaches and separate guidelines on robotic and laparoscopic surgery based on the most recent available body of evidence. The Brescia EGUMIPS guidelines were developed by a large faculty of experts and researchers following an unique combination of a validated and novel methodology covering 8 relevant domains: terminology, indications, patients, procedures, surgical techniques and instrumentation, assessment tools, implementation and training, and artificial intelligence.

## METHODS

The guideline development followed 3 validated methodologies, which had previously been used in the development of the Miami guidelines. The Scottish Intercollegiate Guidelines Network (SIGN) methodology was used to assess the evidence and develop guideline recommendations by working groups consisting of Experts and Researchers,^19^ the Delphi method to establish consensus on the recommendations among the Expert Committee,^20^ and the AGREE II-GRS tool for methodological guideline quality assessment and external validation by the Validation Committee.^21^ The Validation Committee functioned independently, as it did not participate in formulating the recommendations and did not receive any information regarding the specific details of the guidelines before the meeting. They were only regularly updated on the ongoing overall process by the Chairman. To validate and assess the public voting process, to evaluate the interaction between the Expert and Validation Committee, and to ensure all methodologies were followed correctly, an independent Jury Committee was appointed. The Jury Committee completed a specifically designed form after each meeting day to assess quality aspects of the guidelines development process.

Before the development of the guidelines, these different Committees were established. First, a Steering Committee of 6 members was established on the grounds of their clinical and scientific expertise and knowledge in MIPS. This committee and the congress Chairman identified an Expert Committee of 21 European and 6 international experts, a Validation Committee of 22 members including 17 pancreatic surgeons, 2 methodologists, and 3 patients’ representatives, a Jury Committee of 11 members, and a Research Committee of 18 members dedicated to research in MIPS (Supplementary Appendix S1-6, Supplemental Digital Content 1, http://links.lww.com/SLA/E730). In the Expert Committee and Validation Committee, a geographically balanced selection was ensured between surgeons practicing only open surgery, surgeons practicing mainly minimally invasive surgery, and surgeons practicing both. After the group selection, 8 key domains for guideline development were identified by the congress Chairman and the Steering Committee, which included: terminology, indications, patient selection, procedures, surgical techniques and instrumentation, assessment tools, implementation and training, and artificial intelligence. All domains were subsequently subdivided into 22 relevant topics with a total of 29 clinical questions on laparoscopic, 29 on robotic, and 18 on general MIPS, created and reviewed by the Chairman and the Steering Committee. The Expert Committee, the Steering Committee, and the Research Committee members were divided into working groups over the 8 different domains. Steering Committee members participated in 2 domains. Experts were allocated to a domain based on their expertise, and an equal number of laparoscopic-focused surgeons and robotic-focused surgeons within each domain was assured. Experts were allocated to either the laparoscopic or the robotic questions based on their common practice approach to achieve reliable and separate evidence on both approaches. Eventually, each working group consisted of 4-6 experts (2-3 laparoscopic and 2-3 robotic) and 3-5 surgical researchers.

For every domain, systematic reviews of available literature on robotic and laparoscopic pancreatic surgery, including comparative studies with open pancreatic surgery, were performed by the working groups using the PubMed, Embase, and Cochrane databases (the overall PRISMA diagram is shown in Supplementary Figure 1, Supplemental Digital Content 1, http://links.lww.com/SLA/E730). Studies with a minimum sample size of 20 patients and published in the English language were included. All studies found eligible after screening were reviewed and summarized in separate evidence tables. The SIGN methodology was used to score the quality of each study and assign a level of evidence (Supplementary Appendix S7, Supplemental Digital Content 1, http://links.lww.com/SLA/E730).^19^ Based on the evidence and their quality, recommendations were formulated for each clinical question by the experts of the working group. A form of recommendation, based on a GRADE rating (ie, strong or weak, Supplementary Appendix S8, Supplemental Digital Content 1, http://links.lww.com/SLA/E730),^22^ was given for every recommendation as well. Each group delivered their final recommendations with a GRADE rating and the included evidence to the Chairman. In total, 4 online meetings were held with the Research Committee and the Chairman to evaluate the literature review process (dates March 8, 2022, April 2, 2022, May 4, 2022, June 7, 2022). The recommendations of all domains were merged into a questionnaire and circulated to the experts for a first voting round, per the Delphi methodology.^20^ Experts had the voting option to agree or disagree with a particular recommendation and could also comment. Recommendations that achieved an agreement rate of 80% or higher were approved; otherwise, recommendations were returned to the original working group to be revised. Revised recommendations were entered into a second Delphi voting round, after which the identical procedure was repeated. Results of the voting rounds were only accessible by the Chairman and were kept anonymous. On June 23 and August 4, 2022, the first and second Delphi questionnaires respectively, were sent to all experts. On September 28, 2022, before the official guideline meeting, a premeeting was held with the Chairman, Steering Committee, Expert Committee, and Research Committee. During this meeting, a final third Delphi round was held where comments on all recommendations were discussed and minor changes were made. On September 29 and 30, 2022, an in-person meeting took place.

At the start of the plenary meeting, a professional oath ensuring the commitment to an unbiased process was sworn publically by each Committee leader to the Chairman. During the 2-day meeting, all evidence-based recommendations were presented by each domain working group. After each statement, the attending audience (n= 170, consisting of residents, fellows, or surgeons who registered for the conference through the EGUMIPS website and Expert and Jury committee members) voted using a digital voting device for an agreement or disagreement on the given statement. The final audience vote was immediately shown on the presentation screen for transparency and to encourage the discussion. For each topic, the Validation Committee assessed the guideline process and quality according to the AGREE II-GRS instrument^21^ and reviewed the language of the recommendations. This was done during private Validation Committee sessions after each domain presentation. At the end of the 2-day meeting, the Validation Committee presented a report including the quality scores on each topic and suggestions for adjustments or eliminations. The audience revoted on the recommendations revised by the Validation Committee and that initially received an audience agreement rate below 80%. All other adjustments and suggestions were reviewed and accepted by the Chairman, Steering Committee, and Expert Committee.

The Brescia guidelines were endorsed by the International Hepato-Pancreato-Biliary Association, European-African Hepato-Pancreato-Biliary Association, Society of American Gastrointestinal and Endoscopic Surgeons, Società Italiana di Chirurgia, International Consortium on MIPS, European Consortium on MIPS, Associazione Italiana per lo Studio del Pancreas and Women in Surgery (Supplementary Appendix S9-14, Supplemental Digital Content 1, http://links.lww.com/SLA/E730). Representative members of each of those societies were among the experts participating in the guideline process.

## RESULTS

The 8 domains consisted of 22 topics, including 76 clinical questions; 29 on laparoscopic, 29 on robotic, and 18 on general MIPS. Eventually, 98 evidence-based recommendations were established; 33 for laparoscopic, 34 for robotic, and 31 for general MIPS. A flowchart of the process is shown in Supplementary Figure 2, Supplemental Digital Content 1, http://links.lww.com/SLA/E730. The complete set of laparoscopic (L), robotic (R), and general (G) questions, recommendations, and GRADE rating per domain and topic is provided in Supplementary Table S1, Supplemental Digital Content 1, http://links.lww.com/SLA/E730. A more extensive document, including the expert agreement rate, audience agreement rate, topic quality score, comments, and literature, is provided as Supplementary File A, Supplemental Digital Content 1, http://links.lww.com/SLA/E730.

For many topics, the recommendations for laparoscopic and robotic approaches were similar, although the laparoscopic recommendations generally received a stronger GRADE due to a higher level of evidence. Differences between the laparoscopic and robotic recommendations were mainly observed in the topics of learning curves, cost-effectiveness, and artificial intelligence. Compared with the Miami Guidelines, 3 new domains were introduced: terminology, assessment tools, and artificial intelligence.

### Domain 1: Terminology

In the domain terminology, definitions were established for the different types of surgical approaches and conversions. The recommendations are shown in Table 1. The new set of agreed definitions of surgical approaches is shown in Table 2.

**TABLE 1:** Domain 1 Terminology; Questions and Recommendations on Laparoscopic (L), Robotic (R) and General (G) MIPS

Clinical questions (CQs)		Recommendation (R)	Evidence level	Form of recommendation
Topic 1: Types of surgical approaches				
G1	What other approaches should be considered in data collection, registries, and research, besides the laparoscopic, robot-assisted, and open approach?	In MIPS besides the open, laparoscopic, and robot-assisted approaches, also pure robotic, roboscopic, combined, hand-assisted, and single-port approaches should be reported in the surgical series, as defined in Table 2.	Expert opinion	Strong (upgraded by experts)
G2	Should there be a different terminology if combined approaches are used simultaneously versus subsequently?	In MIPS, the terminology for combined simultaneous and subsequent approaches during the index procedure should not be different.	Expert opinion	Strong (upgraded by experts)
Topic 2: Definition of conversion				
G3	How should we define the passage from a laparoscopic to a robotic approach or vice versa if this was not intended in a a. nonurgent situation	a. In pancreatic surgery, a nonurgent change between different minimally-invasive modalities is not a conversion and should be defined as presented in Table 2.	Expert opinion	Strong (upgraded by experts)
	b. urgent situation	b. When the switching from one approach to another is caused by an emergency, it should be reported in the surgical series as a conversion to elucidate its impact on surgical outcomes.	Expert opinion	Strong (upgraded by experts)
L4	Do all conversions to open in LS have the same impact on patients’ outcomes?	In laparoscopic pancreatic surgery, urgent conversions are usually associated with an adverse impact on patients’ outcomes compared with nonurgent conversions. An effort should be made to perform an elective conversion before getting into an emergency conversion.	Low	Strong (upgraded by experts)
R4	Do all conversions to open in RAS have the same impact on patients’ outcomes?	In robot-assisted pancreatic surgery, urgent conversions are usually associated with an adverse impact on patients’ outcomes compared with nonurgent conversions. An effort should be made to perform an elective conversion before getting into an emergency conversion.	Low	Strong (upgraded by experts)
L5	How should we define a nonurgent conversion in LS?	In laparoscopic pancreatic surgery, a “nonurgent conversion” is a conversion to laparotomy for unexpected conditions (I.g. tumor extension/adhesions to adjacent organs/equipment failure) but not in an emergency setting. During the conversion phase, the patient’s vital parameters are stable, and there is no active bleeding	Low	Strong (upgraded by experts)
R5	How should we define a nonurgent conversion in RAS?	In robot-assisted pancreatic surgery, a “nonurgent conversion” is a conversion to laparotomy for unexpected conditions (I.g. tumor extension/adhesions to adjacent organs/equipment failure) but not in an emergency setting. During the conversion phase, the patient’s vital parameters are stable, and there is no active bleeding	Low	Strong (upgraded by experts)
L6	How should we define an urgent conversion in LS?	In laparoscopic pancreatic surgery, an “urgent conversion” is an unplanned conversion for unexpected potentially life-threatening conditions such as bleeding or other conditions affecting patients’ vital parameters.	Low	Strong (upgraded by experts)
R6	How should we define an urgent conversion in RAS?	In robot-assisted pancreatic surgery, an “urgent conversion” is an unplanned conversion for unexpected potentially life-threatening conditions such as bleeding or other conditions affecting patients’ vital parameters.	Low	Strong (upgraded by experts)
L7	How should we define an unintended conversion in LS (I.g. gastrojejunostomy performed open, even though it was initially planned laparoscopically)?	In laparoscopic pancreatic surgery, the unplanned use of a laparotomy to complete the procedure must be defined as a nonurgent conversion.	Expert opinion	Strong (upgraded by experts)
R7	How should we define an unintended conversion in RAS (I.g. gastrojejunostomy performed open, even though it was initially planned laparoscopically)?	In robot-assisted pancreatic surgery, the unplanned use of a laparotomy to complete the procedure must be defined as a nonurgent conversion.	Expert opinion	Strong (upgraded by experts)

LS indicates laparoscopic surgery; MIPS, minimally invasive pancreatic surgery; RAS, robot-assisted surgery.

**TABLE 2:** Terminology and Definitions of Surgical Approaches

Approach	Definition
Laparoscopic	The procedure is fully performed through laparoscopic ports.
Roboscopic (This applies to PD/TP/CP)	The procedure is performed minimally invasively, using both laparoscopic and robot-assisted approaches. It is characterized by the placement of 3–4 robotic ports and 1 or more laparoscopic ports. The robot can be docked at any time during the surgery. The resection is performed laparoscopically and reconstructive phase by combining the laparoscopic and robotic techniques. At least 1 anastomosis is performed using the robot-assisted technique.
Pure Robotic	The procedure is performed through 3–4 robotic ports and 1 or more laparoscopic ports. The robot is docked at the beginning of the surgery. Both the pancreas resection and reconstructive phase (when expected) are carried out using robotic instruments. No laparoscopic energy device is used in pure-robotic procedures.
Robot-assisted (This applies to PD/TP/CP/DP)	The procedure is performed through 3–4 robotic ports and 1 or more laparoscopic ports. The robot is docked at the beginning of the surgery. The pancreas resection phase is carried out using both robotic and laparoscopic instruments. When expected, the reconstructive phase is carried out using exclusively robotic instruments.
Open	The procedure is fully performed through a laparotomy incision without the use of any minimally invasive technique.
Hand-assisted Laparoscopic	The procedure is performed through laparoscopic ports and an auxiliary hand port. The procedure is performed laparoscopically, 1 surgeon’s hand is placed through the hand port and mostly used for retraction and palpation.
Hand-assisted Robotic	The procedure is performed under robotic assistance and through robotic ports. An auxiliary hand-port is also used. The procedure is performed robotically, 1 surgeon’s hand is placed through the hand port and mostly used for retraction and palpation.
Single-port Laparoscopic	The procedure is performed through a single glove port using several either standards or specific laparoscopic instruments.
Single-port Robot-assisted	The procedure is performed using a single specific robotic access with or without an additional robotic/laparoscopic port.
Combined Robot-assisted/Open	It is a combined robotic/open procedure. The resection phase of the procedure is performed with a robot-assisted approach. During the reconstructive phase, at least 1 of the anastomoses is performed by a mini-laparotomy. This procedure is further classified according to the number of anastomoses performed with open approach as follows: **Type I: only 1 anastomosis** **Type II: 2 or more anastomoses**
Combined Laparoscopic/Open	It is a combined laparoscopic/open procedure. The resection phase of the procedure is performed with a laparoscopic approach. During the reconstructive phase, at least one of the anastomoses is performed by a mini-laparotomy. This procedure is further classified according to the number of anastomoses performed with the open approach as follows: **Type I: only 1 anastomosis** **Type II: 2 or more anastomoses**
Combined Roboscopic/Open	It is a combined robotic /laparoscopic/open procedure. The resection and reconstructive phase (when expected) are performed by combining the laparoscopic and/or robot-assisted with open approaches. During the reconstructive phase, at least 1 of the anastomoses is performed by a mini-laparotomy. This procedure is further classified according to the number of anastomoses performed with the open approach as follows: **Type I: only 1 anastomosis** **Type II: 2 or more anastomoses** When the surgery is completed by a laparotomy, it should be defined as “converted”.
Converted	Any minimally invasive pancreatic resection laparoscopic/robotic/roboscopic/combined that required a formal not-intended conversion to laparotomy at any stage of the procedure (resection or reconstruction) for any reasons (bleeding, vascular resection, difficult anastomosis, not-progression, etc.)

CP indicates central pancreatectomy; DP, distal pancreatectomy; PD, pancreatoduodenectomy; TP, total pancreatectomy.

### Domain 2: Indications

In the domain indications, both laparoscopy and robot-assisted were considered alternative approaches to distal pancreatectomy and pancreatoduodenectomy in the treatment of benign, premalignant, and malignant lesions when performed by experienced surgeons in high-volume centers. The strengths of the recommendations were higher for those related to distal pancreatectomy. The recommendations were not profoundly different from the Miami Guidelines and are shown in Supplementary Table S1, Supplemental Digital Content 1, http://links.lww.com/SLA/E730.

### Domain 3: Patients

In the domain patients, no contraindications were identified for laparoscopic and robotic pancreatic resections regarding age, obesity, previous abdominal surgery, and size of the lesion (see Supplementary Table S1, Supplemental Digital Content 1, http://links.lww.com/SLA/E730), as also stated in the Miami Guidelines. Scarce evidence exists regarding the use of vascular resection and neoadjuvant therapy before laparoscopic and robotic pancreatic resections. Further investigation into this topic is warranted.

### Domain 4: Procedures

The recommendations of the domain procedures are shown in Table 3. Both the laparoscopic and robot-assisted approaches were considered appropriate alternatives for enucleation, total pancreatectomy, and vessel-sparing and vessel-resecting spleen-preserving distal pancreatectomy. The role of both approaches in central pancreatectomy has yet to be determined. Also, there is insufficient evidence to define a superior anasomostic technique during robotic and laparoscopic pancreatoduodenectomy, and it remains at the surgeon’s preference.

**TABLE 3:** Domain 4 Procedures; Questions and Recommendations on Laparoscopic (L), Robotic (R), and General (G) MIPS

Clinical questions (CQs)		Recommendation (R)	Evidence level	Form of recommendation
Topic 7: Pancreatoduodenectomy				
L17	What is the preferred anastomosis technique in LPD?	There is insufficient evidence to define a superior anastomotic technique during LPD. The choice of anastomosis during LPD is the surgeon’s preference.	Expert opinion	Weak
R17	What is the preferred anastomosis technique in RPD?	There is insufficient evidence to define a superior anastomotic technique during RPD. The choice of anastomosis during RPD is the surgeon’s preference.	Expert opinion	Weak
Topic 8: Distal Pancreatectomy				
L18	What are the recommendations on LS for the different spleen-preserving techniques?	In laparoscopic spleen preserving distal pancreatectomy, both vessel-sparing and vessel-resecting techniques are appropriate alternatives for the treatment of benign and premalignant diseases.	Low	Strong (upgraded by experts)
R18	What are the recommendations on RAS for the different spleen-preserving techniques?	In robot-assisted spleen-preserving distal pancreatectomy, both vessel-sparing and vessel-resecting techniques are appropriate alternatives for the treatment of benign and premalignant diseases.	Low	Strong (upgraded by experts)
Topic 9: Parenchymal-sparing				
L19	What is the role of LS in central pancreatectomy, regardless of indication?	The role of LS in central pancreatectomy has yet to be determined. Future studies are recommended.	Low	Strong
R19	What is the role of RAS in central pancreatectomy, regardless of indication?	The role of RAS in central pancreatectomy has yet to be determined. Future studies are recommended.	Low	Strong
L20	What is the role of LS in enucleation?	Laparoscopic enucleation of pancreatic lesions in selected patients should be considered as an appropriate alternative to open enucleation.	Moderate	Strong (upgraded by experts)
R20	What is the role of RAS in enucleation?	Robot-assisted enucleation of pancreatic lesions in selected patients should be considered as an appropriate alternative to open enucleation.	Moderate	Strong (upgraded by experts)
Topic 10: Total Pancreatectomy				
L21	What is the role of LS in total pancreatectomy, taking into account different indications?	Laparoscopic total pancreatectomy is an alternative approach to open total pancreatectomy when performed in selected patients by experienced surgeons in high-volume centers.	Low	Weak
R21	What is the role of RAS in total pancreatectomy, taking into account different indications?	Robot-assisted total pancreatectomy is an alternative approach to open total pancreatectomy when performed in selected patients by experienced surgeons in high-volume centers.	Low	Weak

LPD indicates laparoscopic pancreatoduodenectomy; LS, laparoscopic surgery; MIPS, minimally invasive pancreatic surgery; RAS, robot-assisted surgery; RPD, robot-assisted pancreatoduodenectomy.

### Domain 5: Surgical Techniques and Instrumentation

In the domain of surgical techniques and instrumentation, a wide set of recommendations is provided for techniques in pancreatoduodenectomy and distal pancreatectomy, surgical devices, vessel and hemorrhage control, stump closure after distal pancreatectomy, and drain management. The recommendations are shown in Tables 4–6.

**TABLE 4:** Domain 5 Surgical Techniques and Instrumentation; Questions and Recommendations on Laparoscopic (L), Robotic (R) and General (G) MIPS

Clinical questions (CQs)		Recommendation (R)	Evidence level	Form of recommendation
Topic 11: Techniques in Pancreatoduodenectomy				
G22	What are the anatomic landmarks when performing a minimally invasive Kocher Maneuver?	22.1. For the safe completion of the Kocher maneuver during MIPS, it is advised to follow these landmarks: a. Medial edge: exposure of the inferior vena cava (up to the right edge of the aorta) to identify the left renal vein and the origin of the superior mesenteric artery. b. Anterior edge: entire visualization of the entire posterior surface of the head of the pancreas. c. Inferior edge: mobilization of the duodenum from the transverse mesocolon up to the right margin of the ligament of Treitz beneath the superior mesenteric vessels. d. Superior edge: hepatic caudate lobe.	Expert opinion	Weak
		22.2. To safely accomplish specific artery-first approaches and venous vascular control during MIPS, a wider mobilization to expose the SMA may be necessary.	Expert opinion	Weak
G23	Is there a specific indication toward the artery-first approach in MIPD?	23.1 An artery-first approach is feasible during MIPD. The indications between MIPD and OPD are the same.	Low	Strong (upgraded by experts)
		23.2 The artery-first approach during MIPD should be tailored on a case-by-case basis. Surgeons should be aware of each approach (anterior, posterior, left, right, and combined) to SMA dissection keeping in mind that the right SMA approach could be appropriate but may reveal limitations in specific patients in which combined approaches are recommended.	Low	Weak
G24	At what stage should the pancreatic parenchyma be divided?	24.1 Standardization of the timing of surgical steps, including pancreatic transection, to safely perform MIPD is recommended when possible.	Low	Weak
		24.2 Dividing the pancreas after a broad dissection from the portal-mesenteric axis at both the upper and lower edges of the pancreatic neck and possibly completing a retropancreatic tunnel and a broad Kocher maneuver is advisable during MIPD.	Low	Weak
		24.3 In MIPD, the pancreatic neck is preferentially divided from the inferior to the superior margin. This approach leads to the identification of the main pancreatic duct, which could be selectively divided with cold scissors.	Low	Strong (upgraded by experts)
G25	Are there any benefits or specific indications for the biliary tree’s early or delayed division?	In MIPD, biliary duct division is performed after clear visualization of the pertinent vascular anatomy, including aberrant arteries. The timing of the division is the surgeons’ preference	Low	Weak

MIPD indicates minimally invasive pancreatoduodenectomy; MIPS, minimally invasive pancreatic surgery; OPD, open pancreatoduodenectomy; SMA, superior mesenteric artery.

**TABLE 5:** Domain 5 Surgical Techniques and Instrumentation; Questions and Recommendations on Laparoscopic (L), Robotic (R), and General (G) MIPS

Topic 12: Techniques in Distal Pancreatectomy				
G26	What is the best approach for the dissection/control of the splenic vessels?	26.1 When appropriate, dissection between the pancreas and splenic vessels should be carefully performed with a combination of blunt dissection and energy devices after complete mobilization of the colonic splenic flexure.	Low	Strong (upgraded by experts)
		26.2 Careful attention should be given to control small arterial and venous branches into the pancreas (with clips and/or energy devices) when splenic vessels need to be preserved.	Low	Strong (upgraded by experts)
		26.3 A tailored approach to the splenic artery should be encouraged according to individual cases and vascular anatomy. Surgeons should be familiar with both the anterior and posterior approaches.	Low	Strong (upgraded by experts)
		26.4 When dividing the pancreas at the level of the neck, clear visualization of the splenic/portal vein junction should be obtained before ligation and division of the splenic vein. When dividing the pancreas to the left of the celiac trunk, the splenic vessels could be individually ligated or incorporated in the pancreatic division according to surgeon preference.	Low	Strong (upgraded by experts)
		26.5 Accurate preoperative planning and revision of imaging are recommended to evaluate the patient’s arterial and venous vascular anatomy to safely approach splenic vessels.	Low	Strong (upgraded by experts)
G27	Is there any indication for a pancreatic hanging maneuver in MIPD	The pancreatic hanging maneuver is an appropriate option during MIDP.	Low	Strong (upgraded by experts)
Topic 13: Surgical Devices				
G28	What type of energy and instruments should be used during the dissection phase?	The choice of energy devices and instruments for dissection during MIPS should be based on surgeons’ preferences.	Low	Strong (upgraded by experts)
G29	What is the role of the hand-assisted technique for pancreatic resections?	There is a limited role for hand-assisted procedures in contemporary minimally invasive pancreatic surgical practice.	Low	Strong (upgraded by experts)
Topic 14: Vessel and Hemorrhage control				
G30	Is there any approach indicated when venous resections are considered during MIPD?	30.1 A careful expansion of selection criteria for MIPD to include major venous resections can be an option for highly experienced pancreatic surgeons in high-volume centers. Surgeons performing minimally invasive vascular resection should participate in a registry or have a prospectively maintained database to follow their outcomes.	Low	Weak
		30.2 Reserving the venous resection as the final step of a MIPD once dissection is completed and after correct exposure of the portal-mesenteric axis is recommended to minimize clamp time.	Low	Strong (upgraded by experts)
G31	Is there any approach indicated when arterial resections are considered during MIPS?	Arterial resection and/or reconstruction open or MI is not common practice. The MI approach for arterial resection/reconstruction or DP with celiac axis resection can be performed by highly experienced pancreatic surgeons in carefully selected pancreas tumors. Surgeons performing minimally invasive vascular resection should participate in a registry or have a prospectively maintained database to follow and report their outcomes	Low	Strong (upgraded by experts)

DP indicates distal pancreatectomy; MI, minimally invasive; MIPS, minimally invasive pancreatic surgery; MIDP, minimally invasive distal pancreatectomy; MIPD, minimally invasive pancreatoduodenectomy.

**TABLE 6:** Domain 5 Surgical Techniques and Instrumentation; Questions and Recommendations on Laparoscopic (L), Robotic (R), and General (G) MIPS

G32	What are the optimal techniques for the control of hemorrhage during MIPS?	32.1 Of paramount importance in minimizing excessive blood loss during MIPS is optimizing prevention strategies by assuring adequate exposure, gentle dissection, and securing critical vessels.	Low	Strong (upgraded by experts)
		32.2 Targeted interventions should be applied to treat intraoperative bleeding based on the extent and type of bleeding vessels. Bipolar cautery could be used to stop limited bleeding from small venous branches. Moderate venous bleeding can be temporally controlled by gauze compression and then by venous or arterial vessel clipping or suturing.	Low	Strong (upgraded by experts)
G33	What are the optimal techniques for control of hemorrhage during MIDP with spleen preservation?	33.1 Proximal preparation and slinging of the splenic artery and vein before proceeding with pancreatic dissection is suggested during a Kimura’s MI spleen preserving DP. This will allow their temporary clamping in case of hemorrhage or definitive section (Warshaw’s MIDP/splenectomy) if hemostasis is not achieved.	Low	Strong (upgraded by experts)
		33.2 Avoiding splenic injury is important during spleen-preserving pancreatic resections. Surgeons should be familiar with the best surgical practices to stop splenic bleeding.	Low	Strong (upgraded by experts)
Topic 15: Stump closure after Distal Pancreatectomy				
G34	What are the technical details of pancreatic stump transection with staple devices indicated for the division of pancreatic parenchyma in MIDP?	34.1 In MIDP, a standardized technique for using a stapler to obtain adequate pancreatic stump compression is not available, although a gradual stepwise compression is advised.	Low	Strong (upgraded by experts)
		34.2 The optimal choice of cartridges tailored to pancreatic parenchymal features is currently lacking and should be further investigated.	Low	Weak
L35	Should staple versus another type of closure be used for the stump closure in LDP?	A stapling device can be considered for pancreatic stump closure in LDP. However, there are no clear advantages over other pancreatic stump closure techniques to prevent postoperative pancreatic fistula.	Moderate	Strong
R35	Should staple versus another type of closure be used for the stump closure in RDP?	A stapling device can be considered for pancreatic stump closure in RDP. However, there are no clear advantages over other pancreatic stump closure techniques to prevent postoperative pancreatic fistula.	Moderate	Strong
L36	Should staple line reinforcement versus no reinforcement be used for stump closure in LDP when a stapler is used?	Available evidence shows that the standard use of staple line reinforcements for pancreatic stump closure in LDP demonstrates no statistical, clinical benefits over no reinforcement stapling.	Moderate	Strong
R36	Should staple line reinforcement versus no reinforcement be used for stump closure in RDP when a stapler is used?	Available evidence shows that the standard use of staple line reinforcements for pancreatic stump closure in RDP demonstrates no statistical, clinical benefits over no reinforcement stapling	Moderate	Strong
Topic 16: Drain management				
L37	Are there any specific recommendations on the use and positioning of drains in LDP other than those known in the traditional open approach?	There is limited evidence to support the routine use of drains in LDP. Further studies are required.	Low	Strong
R37	Are there any specific recommendations on the use and positioning of drains in RDP other than those known in the traditional open approach?	There is limited evidence to support the routine use of drains in RDP. Further studies are required.	Low	Strong
L38	Are there any specific recommendations on the use and positioning of drains in LPD other than those known in the traditional open approach?	Drain placement could be considered during LPD depending on patient, pancreas, and procedure risks, regardless of the approach. However, no evidence exists on the specific use of drains in LPD.	Moderate	Strong
R38	Are there any specific recommendations on the use and positioning of drains in RPD other than those known in the traditional open approach?	Drain placement could be considered during RPD depending on patient, pancreas, and procedure risks, regardless of the approach. However, no evidence exists on the specific use of drains in RPD.	Moderate	Strong

DP indicates distal pancreatectomy; LDP, laparoscopic distal pancreatectomy; LPD, laparoscopic pancreatoduodenectomy; MI, minimally invasive; MIDP, minimally invasive distal pancreatectomy; MIPS, minimally invasive pancreatic surgery; RDP, robot-assisted distal pancreatectomy; RPD, robot-assisted pancreatoduodenectomy.

### Domain 6: Assessment Tools

In the domain assessment tools, the following core parameters in the assessment of laparoscopic and robotic MIPS were defined: severe morbidity, mortality, postoperative pancreatic fistula, conversion rate, and patient-reported outcomes. R0 resection rate, 3-year overall survival, and disease-free survival were considered core outcomes for PDAC. Several outcome measurements such as Benchmarks, Textbook Outcome, Comprehensive Complication Index, and Clavien-Dindo classification and patient outcomes as Patient Reported Outcome Measures (PROMs) and Quality-Adjusted Life Year (QALY) were considered suitable to assess the validity and efficacy of laparoscopic and robotic pancreatic resections, but it was deemed necessary to develop a multidimensional composite outcome measure to assess the entire operative process and validity (see Supplementary Table S1, Supplemental Digital Content 1, http://links.lww.com/SLA/E730).

### Domain 7: Implementation and Training

In the domain of implementation and training, new insight is provided on center volumes, learning curves, and the cost-effectiveness of laparoscopic and robotic pancreatic resections, as shown in Table 7.

**TABLE 7:** Domain 7 Implementation and Training; Questions and Recommendations on Laparoscopic (L), Robotic (R) and General (G) MIPS

Clinical questions (CQs)		Recommendation (R)	Evidence level	Form of recommendation
Topic 18: Volumes and Learning Curves				
L41	What center volume should be maintained for the safe implementation of LPR (LPD/LDP)?	Center volume strongly affects outcomes after LPD. Morbidity, mortality, and R0 rate are better when LPD is done in centers performing at least 20 LPD procedures per year. Centers should aim to perform at least 20 LPD procedures per year; however, it may be acceptable for centers to perform a lower volume per year as long as they can demonstrate maintenance of equivalent outcomes and they have a well-trained multidisciplinary pancreas team.	Moderate	Strong
R41	What center volume should be maintained for the safe implementation of RPR (RPD/RDP)?	Center volume strongly affects outcomes after RPD. Morbidity, mortality, and R0 rate are better when RPD is done in centers performing at least 20 RPD procedures per year. Centers should aim to perform at least 20 RPD procedures per year; however, it may be acceptable for centers to perform a lower volume per year as long as they can demonstrate maintenance of equivalent outcomes and they have a well-trained multidisciplinary pancreas team.	Moderate	Strong
L42	What are the suggested learning curves and surgeon volumes for LPR (LPD/LDP)?	The learning curve for operative time is 16 procedures for LDP and 39 for LPD. The learning curve for postoperative complications is 25 procedures for LDP and 25–80 for LPD. During the learning curve, surgeons are recommended to participate in a structured training program and ensure that competency is reached.	Moderate	Strong
R42	What are the suggested learning curves and surgeon volumes for RPR (RPD/RDP)?	The learning curve for operative time is 15 procedures for RDP and 25 for RPD. The learning curve for postoperative complications is 21 for RDP and 25–40 for RPD. During this period, surgeons are recommended to participate in a structured training program and ensure that competency is reached.	Moderate	Strong
Topic 19: Training				
L43	What training and preparation should surgeons pursue before performing LPR, and what is their impact?	A potentially higher rate of severe complications suggests the need for caution in introducing LPR techniques. Procedure-specific training programs for LPR mitigated the learning curve. Formal mentorship and structured training programs, which could include virtual reality, bio tissue drills, and off-site and on-site proctoring, facilitate the safe introduction and expansion of LPR.	Moderate	Weak
R43	What training and preparation should surgeons pursue before performing RPR, and what is their impact?	A potentially higher rate of severe complications suggests the need for caution in introducing RPR techniques. Procedure-specific training programs for RPR mitigated the learning curve. Formal mentorship and structured training programs, which could include virtual reality, bio tissue drills, and off- and on-site proctoring, facilitate the safe introduction and expansion of RPR.	Moderate	Weak
Topic 20: Registries				
G44	What should be the role of national and international registries in the wider implementation of MIPS?	The wider implementation of MIPS should be promoted by national and international HPB associations who should strongly encourage the development, implementation, and coordination of national registries and participation in international registries, as it will enhance the position of the country in the international debate and propagate/disseminate collaborative studies, for example, snapshot studies.	Moderate	Strong
G45	Should centers be asked to include patients having MIPS in registries for quality control?	For MIPS, inclusion into registries for quality control by validated national and international centralized registries should be strongly encouraged to allow for transparent analysis and discussions for surgical procedures over time and new surgical techniques.	Moderate	Strong
**Topic 21: Cost-effectiveness**				
L46	Is the laparoscopic approach more costly than the traditional open approach?	The intraoperative costs are higher for LPR compared with OPR but may be offset by the reduction in the length of hospital stay and functional recovery time.	Moderate	Strong
R46	Is the robot-assisted approach more costly than the traditional open approach?	Studies assessing costs for robot-assisted pancreatic surgery are encouraged and should include capital costs, maintenance, and training.	Low	Strong

HPB indicates hepato-pancreato-biliary; LDP, laparoscopic distal pancreatectomy; LPD, laparoscopic pancreatoduodenectomy; LPR, laparoscopic pancreatic resections; MIPS, minimally invasive pancreatic surgery; OPR, open pancreatic resections; RDP, robot-assisted distal pancreatectomy; RPD, robot-assisted pancreatoduodenectomy; RPR, robot-assisted pancreatic resections.

### Domain 8: Artificial Intelligence

The first established recommendations on the role of artificial intelligence in future MIPS are shown in supplementary Table S1, Supplemental Digital Content 1, http://links.lww.com/SLA/E730. Artificial intelligence in MIPS is expected to impact all areas of surgical practice, from preoperative risk assessment and surgical planning to augmenting surgeons’ intraoperative abilities up to tailored follow-up strategies. However, as of now, surgery should not be done without the control of a human surgeon. Surgeons should be encouraged to facilitate the development of artificial intelligence data gathering.

## DISCUSSION

The first internationally validated evidence-based guidelines finalized during the EGUMIPS meeting in Brescia (September 2022) provide 33 recommendations on laparoscopic, 34 on robotic, and 31 on general MIPS for 22 topics in 8 domains.

The Brescia guidelines build on the Miami guidelines published in 2019 and incorporate the body of evidence developed since then, as well as introduce new domains that have recently gained interest. These evidence-based consensus guidelines have been developed by a large number of European and international experts in pancreatic surgery by a strict guideline methodology. Emphasis was placed on the individual aspects of the robotic and laparoscopic approach. Both laparoscopic and robotic experts were carefully selected in the preparation process, and the questions and literature review were separated for both approaches. Although the recommendations for both approaches appeared to be largely similar, the learning curves for robotic MIPS are reportedly shorter compared with laparoscopic MIPS, laparoscopic MIPS is cost-effective while the cost-effectiveness of robotic MIPS remains unclear, and artificial intelligence is expected to be crucial in future robotic MIPS.

While there was already a lot of evidence on laparoscopic distal pancreatectomy and laparoscopic pancreatoduodenectomy (LPD), robotic distal pancreatectomy and robotic pancreatoduodenectomy (RPD) have increasingly been studied in recent years due to the latest emergence of robotic platforms. After conflicting results from the multicenter LEOPARD-2 trial^10^ that raised concerns regarding the safety of LPD, on the one hand, and the PLOT trial^8^ and the PADULAP trial^9^ that reported positive results after LPD, on the other, interest in the role of RPD increased. The recently completed German monocenter EUROPA trial (DRKS00020407), the ongoing European multicenter DIPLOMA-2 trial (ISRCTN27483786), and the Chinese multicenter PORTAL trial (NCT04400357) are expected to shed more light on this. Initially, the Validation Committee and audience disagreed with some of the experts’ recommendations on the domain “indications.” Wording such as “should be considered” was perceived as overly firm given the limited currently available and debatable evidence. A constructive discussion within the Validation Committee led to the proposal to soften and clarify the statements by changing the wording. When the public voted again on those revised statements, all of them achieved at least 80% agreement. Moreover, the domain “indications” was further divided into specific indications such as benign, premalignant, and malignant lesions to reflect the different levels of severity or potential for harm associated with each type of condition. Studies that reported outcomes for all indications only were therefore excluded during the literature review phase. As a result, the current established guidelines are based on studies that reported outcomes separately for benign, premalignant, or malignant indications. In our opinion, this makes the current guidelines more reliable.

Besides indications, surgeon learning curves and minimum center volumes were the most debated topics. Defining center volumes and learning curves was considered crucial to guarantee the safety of MIPS. However, it also raised the fear that a universal minimum number of cases per year considered acceptable for maintaining skills cannot be reached worldwide, mostly because of low volume in some countries or the lack of centralization. In addition, cultural work dynamics have their impact. For example, even in countries where centralization is strongly implemented, a unit with a larger number of surgeons working independently would still not allow one surgeon to perform 20 MIPDs. While in other countries, multiple surgeons may be involved in the same procedure, each performing and registering one part of the procedure as the first operator, thus making it difficult to assess the true number of procedures performed per surgeon and, herewith, the real learning curve. Moreover, several confounding factors can strongly affect the learning curve assessment and definition. Those include previous surgical experience, previous MIPS experience, and previous MIS experience in other fields, such as minimally invasive liver surgery, which is common considering that most pancreatic surgeons are also hepatobiliary surgeons. This reflects the difficulty to achieve firm and generalized agreements on those topics, which translated into a lack of audience support during the voting rounds. After the revisions made by the Validation Committee implying that centers with a lower volume but with a well-trained multidisciplinary pancreas team should not be excluded from MIPS when acceptable outcomes are guaranteed, the audience accepted all final recommendations.

Furthermore, the Brescia guidelines introduce 3 important novel domains: terminology, assessment tools, and artificial intelligence (AI). Despite the 2017 IHPBA guidelines on terminology, there is still a large variety in how different approaches are reported as surgeons adopt the robot differently during their surgical procedures. Some report using the robot and only robotic instruments throughout the whole procedure, while others report using a combination of laparoscopic and robotic instruments. Consequently, numerous terms are currently interchangeably used in literature, such as “robot-assisted,” “robotic,” “roboscopic,” and “hybrid,” without clear definitions. However, as those approaches have different surgical, clinical, and economic implications, they would affect the precision and homogeneity of research studies and outcome comparisons. Including a domain on terminology in the Brescia guidelines has now resulted in a new set of terminology and definitions, agreed on with high consensus rates by the experts and the public. “Robotic” should be used as an umbrella term to describe the general use of robotics in MIPS, while “robot-assisted” and “pure-robotic” should be used as procedure-specific terms to differentiate the precise use of the robot during a surgical procedure. The new terminology could not retrospectively be adjusted in the questions and recommendations according to the guideline methodology, but it is agreed that these definitions should now be adopted in databases, registries, and studies to ensure a homogenous language for the whole surgical community. We would support this new terminology being adopted by other surgical specialties as well for the sake of standardization of surgical terminology and clarity in reporting.

Similarly, the domain of AI was included as it is increasingly being used in medical practice and has demonstrated growing applicability. This domain was welcomed by the pancreatic society. Although there is still little evidence available on this topic, consensus could be achieved on 4 recommendations regarding its role and position in future MIPS. Lastly, assessment tools were incorporated as a new domain to comply with the increasing importance of accurate outcome measurement in surgical care. In this domain, all outcome metrics were elaborated, and their applicability was clarified to enable future accurate outcome comparisons. It was, however, stressed that the available outcome metrics do not tell the whole surgical story, leaving a great need for a more holistic end point that takes into account both surgical and in particular, patient aspects. This was also emphasized by the 3 patient representatives who were included in the Validation Committee, since patients’ perspective, in addition to surgical outcomes, is an important indicator of surgical quality. Their suggestions received considerable room and attention during the private Validation Committee deliberations, which enriched the current guidelines with a different point of view.

Compared with the previous guidelines, new evidence has emerged on various topics, including the role of MIPS in cancer, spleen preservation, learning curves, drain management, and center volumes. Still, limited evidence is available on the best anastomotic techniques in MIPD, central pancreatectomy, quality of life, and cost-effectiveness of the robot-assisted approach. Future research is encouraged to explore the advantages of both approaches and address the aforementioned knowledge gaps.

## CONCLUSION

The 2022 EGUMIPS meeting in Brescia has resulted in a large number of evidence-based recommendations on laparoscopic and robotic pancreatic surgery, established by a group of recognized international and European experts in the field of minimally invasive and open pancreatic surgery. The Brescia guidelines provide the most up-to-date evidence and can provide evidence-based guidance to pancreatic surgeons, policy-makers, and patients.

## Supplementary Material
