# Supplementary material for: The Brescia Internationally Validated European Guidelines on Minimally Invasive Pancreatic Surgery (EGUMIPS)
Source: Ann Surg. 2023 Jul 14;279(1):45–57. doi: 10.1097/SLA.0000000000006006 (PMC10727198; doi:10.1097/SLA.0000000000006006)
Supplement: Supplementary file 1 [file sla-279-045-s001.docx]

**SUPPLEMENTARY TABLES**

**Supplementary Table S1**. Summary of clinical questions and recommendations on laparoscopic (L), robotic (R) and general (G) minimally invasive pancreatic surgery.

| **Clinical Questions (CQs)** | | **Recommendation (R)** | **Evidence Level** | **Form of recommendation** |
| --- | --- | --- | --- | --- |
| **DOMAIN 1: TERMINOLOGY**  **Topic 1: Types of surgical approaches** | | | | |
| G1 | What other approaches should be considered in data collection, registries, and research, besides the laparoscopic, the robot-assisted, and the open approach? | In minimally-invasive pancreatic surgery, besides the open, laparoscopic, and robot-assisted approaches, also pure robotic, roboscopic, combined, hand-assisted, and single-port approaches should be reported in surgical series, as defined in Supplementary File A2. | Expert opinion | Strong (upgraded by experts) |
| G2 | Should there be a different terminology if combined approaches are used simultaneously versus subsequently? | In MIPS, the terminology for combined simultaneous and subsequent approaches during the index procedure should not be different. | Expert opinion | Strong (upgraded by experts) |
| **Topic 2: Definition of Conversion** | | | | |
| G3 | How should we define the passage from a laparoscopic to a robotic approach or vice versa if this was not intended in a   1. non-urgent situation 2. urgent situation | 1. In pancreatic surgery, a non-urgent change between different minimally-invasive modalities is not a conversion and should be defined as presented in Supplementary File A2. | Expert opinion | Strong (upgraded by experts) |
|  |  | 1. When the switching from one approach to another is caused by an emergency, it should be reported in surgical series as a conversion to elucidate its impact on surgical outcomes. | Expert opinion | Strong (upgraded by experts) |
| L4 | Do all conversions to open in laparoscopic surgery **(LS)** have the same impact on patients’ outcome? | In laparoscopic pancreatic surgery, urgent conversions are usually associated with an adverse impact on patients’ outcomes compared to non-urgent conversions. An effort should be made to perform an elective conversion prior to getting into an emergency conversion. | Low | Strong (upgraded by experts) |
| R4 | Do all conversions to open in robot-assisted surgery **(RAS)** have the same impact on patients’ outcome? | In robot-assisted pancreatic surgery, urgent conversions are usually associated with an adverse impact on patients’ outcomes compared to non-urgent conversions. An effort should be made to perform an elective conversion prior to getting into an emergency conversion. | Low | Strong (upgraded by experts) |
| L5 | How should we define a non-urgent conversion in LS? | In laparoscopic pancreatic surgery, a “non-urgent conversion” is a conversion to laparotomy for unexpected conditions (i.g. tumor extension/adhesions to adjacent organs/equipment failure) but not in an emergency setting. During the conversion phase, the patient's vital parameters are stable and there is no active bleeding | Low | Strong (upgraded by experts) |
| R5 | How should we define a non-urgent conversion in RAS? | In robot-assisted pancreatic surgery, a “non-urgent conversion” is a conversion to laparotomy for unexpected conditions (i.g. tumor extension/adhesions to adjacent organs/equipment failure) but not in an emergency setting. During the conversion phase, the patient's vital parameters are stable and there is no active bleeding | Low | Strong (upgraded by experts) |
| L6 | How should we define an urgent conversion in LS? | In laparoscopic pancreatic surgery, an “urgent conversion” is an unplanned conversion for unexpected potentially life-threatening conditions such as bleeding or other conditions affecting patients’ vital parameters. | Low | Strong (upgraded by experts) |
| R6 | How should we define an urgent conversion in RAS? | In robot-assisted pancreatic surgery, an “urgent conversion” is an unplanned conversion for unexpected potentially life-threatening conditions such as bleeding or other conditions affecting patients’ vital parameters. | Low | Strong (upgraded by experts) |
| L7 | How should we define an unintended conversion in LS (i.g. gastrojejunostomy performed open, even though it was initially planned laparoscopically)? | In laparoscopic pancreatic surgery, the unplanned use of a laparotomy to complete the procedure must be defined as a non-urgent conversion. | Expert opinion | Strong (upgraded by experts) |
| R7 | How should we define an unintended conversion in RAS (i.g. gastrojejunostomy performed open, even though it was initially planned laparoscopically)? | In robot-assisted pancreatic surgery, the unplanned use of a laparotomy to complete the procedure must be defined as a non-urgent conversion. | Expert opinion | Strong (upgraded by experts) |
| **DOMAIN 2: INDICATIONS**  **Topic 3: Benign and pre-malignant lesions** | | | | |
| L8 | What is the role of LS in the management of benign or pre-malignant lesions in the pancreatic body and tail? | Laparoscopic distal pancreatectomy **(LDP)** should be considered over the traditional open approach in the management of benign or pre-malignant lesions in the pancreatic body and tail, when performed by experienced surgeons in high-volume centers. It is associated with a shorter time to functional recovery, shorter hospital stay, and reduced blood loss with similar complication and CR-POPF rates compared to open distal pancreatectomy. | High | Strong |
| R8 | What is the role of RAS in the management of benign or pre-malignant lesions in the pancreatic body and tail? | 8.1 Robot-assisted distal pancreatectomy **(RDP)** should be considered as a valid approach in the management of benign or pre-malignant lesions in the pancreatic body and tail compared to laparoscopic or open distal pancreatectomy, when performed by experienced surgeons in high-volume centers. | Moderate | Strong |
|  |  | 8.2 RDP is comparable to LDP in terms of splenic preservation and early postoperative results, when performed by experienced surgeons in high-volume centers. | Moderate | Strong |
| L9 | What is the role of LS in the management of benign or pre-malignant lesions in the pancreatic head? | Laparoscopic pancreatoduodenectomy **(LPD)** can be considered as an alternative approach to open pancreatoduodenectomy (OPD) in the management of benign or pre-malignant lesions in the pancreatic head, when performed by experienced surgeons in high-volume centers. | Low | Weak |
| R9 | What is the role of RAS in the management of benign or pre-malignant lesions in the pancreatic head? | Robot-assisted pancreatoduodenectomy **(RPD)** can be considered as an alternative approach to open pancreatoduodenectomy (OPD) in the management of benign or pre-malignant lesions in the pancreatic head, when performed by experienced surgeons in high-volume centers. | Low | Weak |
| **Topic 4: Pancreatic Ductal Adenocarcinoma (PDAC)** | | | | |
| L10 | What is the role of LS in the management of PDAC in the pancreatic body and tail? | Laparoscopic left radical pancreatectomy should be considered as an alternative approach in the management of resectable PDAC in the pancreatic body and tail, when performed by experienced surgeons in high volume centers | Moderate | Weak |
| R10 | What is the role of RAS in the management of PDAC in the pancreatic body and tail? | Robot-assisted left radical pancreatectomy should be considered as an alternative approach in the management of resectable PDAC in the pancreatic body and tail, when performed by experienced surgeons in high-volume centers. | Moderate | Weak |
| L11 | What is the role of LS in the management of PDAC in the pancreatic head? | LPD can be considered as an alternative approach in the management of PDAC in the pancreatic head, when performed by experienced surgeons in high-volume centers. Surgeons performing LPD must participate in a registry or follow their outcomes in a prospectively maintained database. | High | Weak |
| R11 | What is the role of RAS in the management of PDAC in the pancreatic head? | RPD can be considered as an alternative approach in the management of PDAC in the pancreatic head, when performed by experienced surgeons in high-volume centers. Surgeons performing RPD must participate in a registry or follow their outcomes in a prospectively maintained database. | Low | Weak |
| **Topic 5: Periampullary adenocarcinoma** | | | | |
| L12 | What is the role of LS in the management of non-pancreatic periampullary adenocarcinoma (NPPC)? (ampullary, duodenal and cholangiocarcinoma) | LPD is an acceptable alternative for patients with a resectable non-pancreatic periampullary adenocarcinoma (NPPC), when performed by experienced surgeons in high-volume centers. Surgeons performing LPD must participate in a registry or follow their outcomes in a prospectively maintained database. | Low | Weak |
| R12 | What is the role of RAS in the management of non-pancreatic periampullary adenocarcinoma (NPPC)? (ampullary, duodenal and cholangiocarcinoma) | RPD is an acceptable alternative for patients with a resectable non-pancreatic periampullary adenocarcinoma (NPPC), when performed by experienced surgeons in high-volume centers. Surgeons performing RPD must participate in a registry or follow their outcomes in a prospectively maintained database. | Low | Weak |
| **DOMAIN 3: PATIENTS**  **Topic 6: High-risk patients** | | | | |
| L13 | Are there any contraindications for laparoscopic pancreatic resections **(LPR)** related to  13.1 patients age  13.2 obesity  13.3 previous abdominal surgery | 13.1 Age alone should not be a contra-indication for LPD and LDP. LPD and  LDP are appropriate alternatives. | Moderate | Strong |
|  |  | 13.2 Obesity alone should not be considered as a contraindication for performing LDP or LPD. However, obesity is a risk factor for postoperative complications (especially POPF). | Low | Weak |
|  |  | 13.3 Previous abdominal surgery alone should not be considered as an absolute contraindication for LDP/LPD. | Low | Weak |
| R13 | Are there any contraindications for robot-assisted pancreatic resections **(RPR)** related to  13.1 patients age  13.2 obesity  13.3 previous abdominal surgery | 13.1 Age alone should not be a contra-indication for RPD. RPD is an appropriate alternative for elderly patients. | Low | Weak |
|  |  | 13.2 Obesity alone should not be considered as a contraindication for performing RDP or RPD. However, obesity is a risk factor for postoperative complications (especially POPF). | Low | Weak |
|  |  | 13.3 Previous abdominal surgery alone should not be considered as an absolute contraindication for RDP/RPD. | Expert opinion | Weak |
| L14 | Are LPR an appropriate alternative in patients with large lesions? | Size of the lesion alone is not a contraindication for LDP. There is no evidence on LPD. | Low | Weak |
| R14 | Are RPR an appropriate alternative in patients with large lesions? | Size of the lesion alone is not a contraindication for RDP. There is no evidence on RPD. | Low | Weak |
| L15 | Are LPR an appropriate alternative in patients with vascular involvement? | Scarce evidence exists regarding the use of vascular resection in LPR. LPD with vascular resection should only be performed by highly experienced surgeons and in high-volume centers. Further investigation is warranted on this topic and data on patient treatment and outcomes needs to be entered in prospective registries and databases. | Low | Weak |
| R15 | Are RPR an appropriate alternative in patients with vascular involvement? | No evidence exists regarding the use of vascular resection in RPR. RPD with vascular resection should only be performed by highly experienced surgeons and in high-volume centers. Further investigation is warranted on this topic and data on patient treatment and outcomes needs to be entered in prospective registries and databases. | Low | Weak |
| L16 | Are LPR indicated for the treatment of PDAC after neoadjuvant therapy? | There is no data available regarding LPD/LDP after neoadjuvant therapy, further investigation is warranted. | Low | Weak |
| R16 | Are RPR indicated for the treatment of PDAC after neoadjuvant therapy? | There is no data available regarding RPD/RDP after neoadjuvant therapy, further investigation is warranted. | Low | Weak |
| **DOMAIN 4: PROCEDURES**  **Topic 7: Pancreatoduodenectomy** | | | | |
| L17 | What is the preferred anastomosis technique in LPD? | There is insufficient evidence to define a superior anastomotic technique during LPD. The choice of anastomosis during LPD is the surgeon’s preference. | Expert opinion | Weak |
| R17 | What is the preferred anastomosis technique in RPD? | There is insufficient evidence to define a superior anastomotic technique during RPD. The choice of anastomosis during RPD is the surgeon’s preference. | Expert opinion | Weak |
| **Topic 8: Distal Pancreatectomy** | | | | |
| L18 | What are the recommendations on LS for the different spleen-preserving techniques? | In laparoscopic spleen preserving distal pancreatectomy, both vessel-sparing and vessel-resecting techniques are appropriate alternatives for the treatment of benign and pre-malignant diseases. | Low | Strong (upgraded by experts) |
| R18 | What are the recommendations on RAS for the different spleen-preserving techniques? | In robot-assisted spleen preserving distal pancreatectomy, both vessel-sparing and vessel-resecting techniques are appropriate alternatives for the treatment of benign and pre-malignant diseases. | Low | Strong (upgraded by experts) |
| **Topic 9: Parenchymal-sparing** | | | | |
| L19 | What is the role of LS in central pancreatectomy, regardless of indication? | The role of LS in central pancreatectomy has yet to be determined. Future studies are recommended. | Low | Strong |
| R19 | What is the role of RAS in central pancreatectomy, regardless of indication? | The role of RAS in central pancreatectomy has yet to be determined. Future studies are recommended. | Low | Strong |
| L20 | What is the role of LS in enucleation? | Laparoscopic enucleation of pancreatic lesions in selected patients should be considered as an appropriate alternative to open enucleation. | Moderate | Strong (upgraded by experts) |
| R20 | What is the role of RAS in enucleation? | Robot-assisted enucleation of pancreatic lesions in selected patients should be considered as an appropriate alternative to open enucleation. | Moderate | Strong (upgraded by experts) |
| **Topic 10: Total Pancreatectomy** | | | | |
| L21 | What is the role of LS in total pancreatectomy, taking into account different indications? | Laparoscopic total pancreatectomy is an alternative approach to open total pancreatectomy when performed in selected patients by experienced surgeons in high volume centers. | Low | Weak |
| R21 | What is the role of RAS in total pancreatectomy, taking into account different indications? | Robot-assisted total pancreatectomy is an alternative approach to open total pancreatectomy when performed in selected patients by experienced surgeons in high volume centers. | Low | Weak |
| **DOMAIN 5: SURGICAL TECHNIQUES AND INSTRUMENTATION**  **Topic 11: Techniques in Pancreatoduodenectomy** | | | | |
| G22 | What are the anatomical landmarks when performing a minimally invasive Kocher Maneuver? | 22.1. For the safe completion of the Kocher maneuver during MIPS, it is advised to follow these landmarks:   1. Medial edge: exposure of the inferior vena cava (up to the right edge of the aorta) to identify the left renal vein and the origin of the superior mesenteric artery. 2. Anterior edge: entire visualization of the entire posterior surface of the head of the pancreas. 3. Inferior edge: mobilization of the duodenum from the transverse mesocolon up to the right margin of the ligament of Treitz beneath the superior mesenteric vessels. 4. Superior edge: hepatic caudate lobe. | Expert opinion | Weak |
|  |  | 22.2. To safely accomplish specific artery first approaches and venous vascular control during MIPS, a wider mobilization to expose the SMA may be necessary. | Expert opinion | Weak |
| G23 | Is there a specific indication toward the artery first approach in minimally invasive pancreatoduodenectomy (MIPD)? | 23.1 An artery first approach is feasible during MIPD. The indications between MIPD and OPD are the same. | Low | Strong (upgraded by experts) |
|  |  | 23.2 The artery first approach during MIPD should be tailored on a case-by-case basis. Surgeons should be aware of each approach (anterior, posterior, left, right, and combined) to SMA dissection keeping in mind that the right SMA approach could be appropriate but may reveal limitations in specific patients in which combined approaches are recommended. | Low | Weak |
| G24 | At what stage should the pancreatic parenchyma be divided? | 24.1 Standardization of the timing of surgical steps, including pancreatic transection, to safely perform MIPD is recommended when possible. | Low | Weak |
|  |  | 24.2 Dividing the pancreas after a broad dissection from the portal-mesenteric axis at both the upper and lower edges of the pancreatic neck and possibly completing a retropancreatic tunnel and a broad Kocher maneuver is advisable during MIPD. | Low | Weak |
|  |  | 24.3 In MIPD, the pancreatic neck is preferentially divided from the inferior to the superior margin. This approach leads to the identification of the main pancreatic duct, which could be selectively divided with cold scissors. | Low | Strong (upgraded by experts) |
| G25 | Are there any benefits or specific indications for the biliary tree's early or delayed division? | In MIPD, biliary duct division is performed after clear visualization of the pertinent vascular anatomy including aberrant arteries. The timing of the division is the surgeons’ preference | Low | Weak |
| **Topic 12: Techniques in Distal Pancreatectomy** | | | | |
| G26 | What is the best approach for dissection/control of the splenic vessels? | 26.1 When appropriate, dissection between the pancreas and splenic vessels should be carefully performed with a combination of blunt dissection and energy devices after complete mobilization of the colonic splenic flexure. | Low | Strong (upgraded by experts) |
|  |  | 26.2 Careful attention should be given to control small arterial and venous branches into the pancreas (with clips and/or energy devices) when splenic vessels need to be preserved. | Low | Strong (upgraded by experts) |
|  |  | 26.3 A tailored approach to the splenic artery should be encouraged according to individual cases and vascular anatomy. Surgeons should be familiar with both the anterior and posterior approaches. | Low | Strong (upgraded by experts) |
|  |  | 26.4 When dividing the pancreas at the level of the neck, clear visualization of the splenic/portal vein junction should be obtained prior to ligation and division of the splenic vein. When dividing the pancreas to the left of the celiac trunk, the splenic vessels could be individually ligated or incorporated in the pancreatic division according to surgeon preference. | Low | Strong (upgraded by experts) |
|  |  | 26.5 Accurate preoperative planning and revision of imaging is recommended to evaluate the patient's arterial and venous vascular anatomy to safely approach splenic vessels. | Low | Strong (upgraded by experts) |
| G27 | Is there any indication for a pancreatic hanging maneuver in minimally invasive distal pancreatectomy (MIDP)? | The pancreatic hanging maneuver is an appropriate option during MIDP. | Low | Strong (upgraded by experts) |
| **Topic 13: Surgical Devices** | | | | |
| G28 | What type of energy and instruments should be used during the dissection phase? | The choice of energy devices and instruments for dissection during MIPS should be based upon surgeons' preferences. | Low | Strong (upgraded by experts) |
| G29 | What is the role of the hand-assisted technique for pancreatic resections? | There is a limited role for hand-assisted procedures in contemporary minimally invasive pancreatic surgical practice. | Low | Strong (upgraded by experts) |
| **Topic 14: Vessel and Hemorrhage control** | | | | |
| G30 | Is there any approach indicated when venous resections are considered during MIPD? | 30.1 A careful expansion of selection criteria for MIPD to include major venous resections can be an option for highly experienced pancreatic surgeons in high-volume centers. Surgeons performing minimally invasive vascular resection should participate in a registry or have a prospectively maintained database to follow their outcomes. | Low | Weak |
|  |  | 30.2 Reserving the venous resection as the final step of a MIPD once dissection is completed and after correct exposure of the portal-mesenteric axis is recommended to minimize clamp time. | Low | Strong (upgraded by experts) |
| G31 | Is there any approach indicated when arterial resections are considered during MIPS? | Arterial resection and/or reconstruction open or MI is not common practice. The MI approach for arterial resection/reconstruction or DP with coeliac axis resection can be performed by highly experienced pancreatic surgeons in carefully selected pancreas tumors. Surgeons performing minimally invasive vascular resection should participate in a registry or have a prospectively maintained database to follow and report their outcomes | Low | Strong (upgraded by experts) |
| G32 | What are the optimal techniques for control of hemorrhage during MIPS? | 32.1 Of paramount importance in minimizing excessive blood loss during MIPS is optimizing prevention strategies by assuring adequate exposure, gentle dissection, and securing critical vessels. | Low | Strong (upgraded by experts) |
|  |  | 32.2 Targeted interventions should be applied to treat intraoperative bleeding based on the extent and type of bleeding vessels. Bipolar cautery could be used to stop limited bleeding from small venous branches. Moderate venous bleeding can be temporally controlled by gauze compression and then by venous or arterial vessel clipping or suturing. | Low | Strong (upgraded by experts) |
| G33 | What are the optimal techniques for control of hemorrhage during MIDP with spleen preservation? | 33.1 Proximal preparation and slinging of the splenic artery and vein before proceeding with pancreatic dissection is suggested during a Kimura's MI spleen preserving DP. This will allow their temporarily clamping in case of hemorrhage or definitive section (Warshaw’s MIDP/splenectomy) if hemostasis is not achieved. | Low | Strong (upgraded by experts) |
|  |  | 33.2 Avoiding splenic injury is important during spleen preserving pancreatic resections. Surgeons should be familiar with best surgical practices to stop splenic bleeding. | Low | Strong (upgraded by experts) |
| **Topic 15: Stump closure after Distal Pancreatectomy** | | | | |
| G34 | What are the technical details of pancreatic stump transection with staple devices indicated for the division of pancreatic parenchyma in MIDP? | 34.1 In MIDP, a standardized technique for using a stapler to obtain adequate pancreatic stump compression is not available, although a gradual stepwise compression is advised. | Low | Strong (upgraded by experts) |
|  |  | 34.2 The optimal choice of cartridges tailored to pancreatic parenchymal features is currently lacking and should be further investigated. | Low | Weak |
| L35 | Should staple versus another type of closure be used for the stump closure in LDP? | A stapling device can be considered for pancreatic stump closure in LDP. However, there are no clear advantages over other pancreatic stump closure techniques to prevent postoperative pancreatic fistula. | Moderate | Strong |
| R35 | Should staple versus another type of closure be used for the stump closure in RDP? | A stapling device can be considered for pancreatic stump closure in RDP. However, there are no clear advantages over other pancreatic stump closure techniques to prevent postoperative pancreatic fistula. | Moderate | Strong |
| L36 | Should staple line reinforcement versus no reinforcement be used for stump closure in LDP when a stapler is used? | Available evidence shows that the standard use of staple line reinforcements for pancreatic stump closure in LDP demonstrates no statistically clinical benefits over no reinforcement stapling. | Moderate | Strong |
| R36 | Should staple line reinforcement versus no reinforcement be used for stump closure in RDP when a stapler is used? | Available evidence shows that the standard use of staple line reinforcements for pancreatic stump closure in RDP demonstrates no statistically clinical benefits over no reinforcement stapling | Moderate | Strong |
| **Topic 16: Drain management** | | | | |
| L37 | Are there any specific recommendations on the use and the positioning of drains in LDP, other than those known in the traditional open approach? | There is limited evidence to support the routine use of drains in LDP. Further studies are required. | Low | Strong |
| R37 | Are there any specific recommendations on the use and the positioning of drains in RDP, other than those known in the traditional open approach? | There is limited evidence to support the routine use of drains in RDP. Further studies are required. | Low | Strong |
| L38 | Are there any specific recommendations on the use and the positioning of drains in LPD, other than those known in the traditional open approach? | Drain placement could be considered during LPD depending on patient, pancreas and procedure risks, regardless of the approach. However, no evidence exists on the specific use of drains in LPD. | Moderate | Strong |
| R38 | Are there any specific recommendations on the use and the positioning of drains in RPD, other than those known in the traditional open approach? | Drain placement could be considered during RPD depending on patient, pancreas and procedure risks, regardless of the approach. However, no evidence exists on the specific use of drains in RPD. | Moderate | Strong |
| **DOMAIN 6: ASSESSMENT TOOLS**  **Topic 17: Tools and Methods** | | | | |
| L39 | Which parameters should be defined as core outcomes in the assessment of LPR? | Core parameters in the assessment of LPR should include:  Severe morbidity, mortality, postoperative pancreatic fistula, conversion rate and patient-reported outcomes. Postoperative complications should be assessed up to 90 days. R0 resection rate, 3-year overall survival, and disease-free survival should be considered core outcomes for PDAC. | Expert opinion | Strong |
| R39 | Which parameters should be defined as core outcomes in the assessment of RPR? | Core parameters in the assessment of RPR should include:  Severe morbidity, mortality, postoperative pancreatic fistula, conversion rate and patient-reported outcomes. Postoperative complications should be assessed up to 90 days. R0 resection rate, 3-year overall survival, and disease-free survival should be considered core outcomes for PDAC. | Expert opinion | Strong |
| L40 | Which are the most suitable outcome measurements that can be used to assess the validity of LPR? | 40.1 The most suitable outcome measurements to assess the validity and efficacy of LPR are procedural outcomes as Benchmarks, Textbook Outcome, Comprehensive Complication Index, and Clavien-Dindo classification and patient outcomes as Patient Reported Outcome Measures (PROMs) and Quality-Adjusted Life Year (QALY). However, system-based outcomes such as costs, time, and resources should also be taken into consideration. All outcome measurements should be applied as appropriate based on the validity and efficacy aspect being measured. | Expert opinion | Strong |
|  |  | 40.2 A multidimensional composite outcome measure should be developed in order to assess the entire operative process and validity of LPR. | Expert opinion | Strong |
| R40 | Which are the most suitable outcome measurements that can be used to assess the validity of RPR? | 40.1 The most suitable outcome measurements to assess the validity and efficacy of RPR are procedural outcomes as Benchmarks, Textbook Outcome, Comprehensive Complication Index, and Clavien-Dindo classification and patient outcomes as Patient Reported Outcome Measures (PROMs) and Quality-Adjusted Life Year (QALY). However, system-based outcomes such as costs, time, and resources should also be taken into consideration. All outcome measurements should be applied as appropriate based on the validity and efficacy aspect being measured. | Expert opinion | Strong |
|  |  | 40.2 A multidimensional composite outcome measure should be developed in order to assess the entire operative process and validity of RPR. | Expert opinion | Strong |
| **DOMAIN 7: IMPLEMENTATION AND TRAINING**  **Topic 18: Volumes and Learning Curves** | | | | |
| L41 | What center volume should be maintained for the safe implementation of LPR (LPD/LDP)? | Center volume strongly affects outcomes after LPD. Morbidity, mortality, and R0 rate are better when LPD is done in centers performing at least 20 LPD procedures per year. Centers should aim to perform at least 20 LPD procedures per year, however it may be acceptable for centers to perform a lower volume per year as long as they can demonstrate maintenance of equivalent outcomes and they have a well-trained multidisciplinary pancreas team. | Moderate | Strong |
| R41 | What center volume should be maintained for the safe implementation of RPR (RPD/RDP)? | Center volume strongly affects outcomes after RPD. Morbidity, mortality, and R0 rate are better when RPD is done in centers performing at least 20 RPD procedures per year. Centers should aim to perform at least 20 RPD procedures per year, however it may be acceptable for centers to perform a lower volume per year as long as they can demonstrate maintenance of equivalent outcomes and they have a well-trained multidisciplinary pancreas team. | Moderate | Strong |
| L42 | What are the suggested learning curves and surgeon volumes for LPR (LPD/LDP)? | The learning curve for operative time is 16 procedures for LDP and 39 for LPD. The learning curve for postoperative complications is 25 procedures for LDP and 25-80 for LPD. During the learning curve surgeons are recommended to participate in a structured training program and ensure that competency is reached. | Moderate | Strong |
| R42 | What are the suggested learning curves and surgeon volumes for RPR (RPD/RDP)? | The learning curve for operative time is 15 procedures for RDP and 25 for RPD. The learning curve for postoperative complications is 21 for RDP and 25-40 for RPD. During this period, surgeons are recommended to participate in a structured training program and assure that competency is reached. | Moderate | Strong |
| **Topic 19: Training** | | | | |
| L43 | What training and preparation should surgeons pursue before performing LPR and what is their impact? | A potentially higher rate of severe complications suggests the need for caution in introducing LPR techniques. Procedure-specific training programs for LPR mitigated the learning curve. Formal mentorship and structured training programs, which could include virtual reality, bio tissue drills, and off- and on-site proctoring facilitate safe introduction and expansion of LPR. | Moderate | Weak |
| R43 | What training and preparation should surgeons pursue before performing RPR and what is their impact? | A potentially higher rate of severe complications suggests the need for caution in introducing RPR techniques. Procedure-specific training programs for RPR mitigated the learning curve. Formal mentorship and structured training programs, which could include virtual reality, bio tissue drills, and off- and on-site proctoring facilitate safe introduction and expansion of RPR. | Moderate | Weak |
| **Topic 20: Registries** | | | | |
| G44 | What should be the role of national and international registries in the wider implementation of MIPS? | The wider implementation of MIPS should be promoted by national and international HPB associations who should strongly encourage the development, implementation and coordination of national registries and participation into international registries, as it will enhance the position of the country in the international debate and propagate/disseminate collaborative studies, e.g., snapshot studies. | Moderate | Strong |
| G45 | Should centers be asked to include patients having MIPS in registries for quality control? | For MIPS, inclusion into registries for quality control by validated national and international centralized registries should be strongly encouraged to allow for transparent analysis and discussions for surgical procedures over time and new surgical techniques. | Moderate | Strong |
| **Topic 21: Cost-effectiveness** | | | | |
| L46 | Is the laparoscopic approach more costly than the traditional open approach? | The intraoperative costs are higher for LPR compared to OPR but may be offset by the reduction in length of hospital stay and functional recovery time. | Moderate | Strong |
| R46 | Is the robot-assisted approach more costly than the traditional open approach? | Studies assessing costs for robot-assisted pancreatic surgery are encouraged and should include capital costs, maintenance and training. | Low | Strong |
| **DOMAIN 8: ARTIFICIAL INTELLIGENCE (AI)**  **Topic 22: The role of AI in pancreatic surgery** | | | | |
| L47 | What is the role of AI in the future expansion of LPR? | 47.1 AI in MIPS is expected to impact all areas of surgical practice, from preoperative risk assessment and surgical planning to augmenting surgeons' intra-operative abilities up to tailored follow-up strategies. Surgeons should be continuously updating themselves with these upcoming innovations and encourage their introduction into their clinical practice once their validity is proven. Surgeons should be encouraged to facilitate the development of AI data gathering. | Low | Weak |
|  |  | 47.2 Enhanced computer vision with augmented reality, virtual reality, and mixed reality are fields of AI that should continue to be developed because these technologies have great potential to improve surgical outcomes and to reduce short-term complications of LPR. | Low | Weak |
| R47 | What is the role of AI in the future expansion of RPR? | 47.1 Autonomous actions are central to AI in future RPR. Today, tools with only a level 1 (telemanipulation – robot-assisted surgery) or a level 2 (limited autonomous actions) and arguably level 3 autonomy are available. Level 4 and 5 autonomy could eventually be possible; however, as of now, surgery should not be done without the control of a human surgeon who is fully capable of performing and completing the procedure via robotic assistance, standard laparoscopy and/or open surgical techniques. Surgeons should be encouraged to facilitate the development of AI data gathering. | Low | Weak |
|  |  | 47.2 Initially, it may be preferable for only parts of pancreatic surgical procedures to become fully automated with an emphasis on creating autonomous dexemes (parts of surgical gestures) and then surgemes (parts of entire operations) that are safe for patients and can be proven to provide patient benefit. | Low | Weak |

**SUPPLEMENTARY FILE A**

**DOMAIN I. TERMINOLOGY**

*Topic 1: Types of surgical approaches Quality score: 85.0%*

**G1. What other approaches should be considered in data collection, registries, and research, besides the laparoscopic, the robot-assisted, and the open approach?**

Recommendation: In minimally-invasive pancreatic resections, besides the open, laparoscopic, and robot-assisted approaches, also pure robotic, roboscopic, combined, hand-assisted, and single-port approaches should be reported in surgical series, as defined in Table 1 (GRADE 1 EXPERT OPINION, expert agreement 86.5% , audience agreement 87.2%).

Comments: In literature, a large variety of different approaches are reported for pancreatic resections. This includes laparoscopic, robot-assisted, open, single-port, hand-assisted, and hybrid procedures. However, for each of these terms, there is no clear definition which makes that they are often used to describe different approaches depending on the study. To address this problem, in 2017, the IHPBA developed guidelines for the standardization of terminology in MIPS.^1^ However, five years after the publication of these guidelines, they are still not universally adopted and a specific terms such as “hybrid” procedure are still in use.
Proposed action: Pancreatic resection approaches should be classified based on the new proposed standardized definition, to allow reliable comparison between surgical series.

**G2. Should there be a different terminology if combined approaches are used simultaneously versus subsequently?**Recommendation: In MIPS, the terminology for combined simultaneous and subsequent approaches during the index procedure should not be different (GRADE 1 EXPERT OPINION, expert agreement 86.5% , audience agreement 88.5%).
Comments: There is no evidence on this topic in the literature.
Proposed action: In future surgical studies, authors should report on combined approaches in the method section.

*Topic 2: Definition of conversion Quality score: 83.0%*
**G3. How should we define the passage from a laparoscopic to a robotic approach or vice versa if this was not intended in a; (A) non-urgent situation, (B) urgent situation.**Recommendation: (A) In pancreatic surgery, a non-urgent change between different minimally-invasive modalities is not a conversion and should be defined as presented in Table 1. (B) When the switching from one approach to another is caused by an emergency, it should be reported in surgical series as a conversion to elucidate its impact on surgical outcomes (GRADE 1 EXPERT OPINION, expert agreement 94.6% , audience agreement 88.8%).
Comments: The IHPBA guidelines for terminology in MIPS define a conversion as the use of two different approaches due to failure to proceed or due to an emergent need to change approach^1^. In this definition, the case of the passage from a laparoscopic to a robotic-assisted approach, and vice versa, is not specified. To date, there is no evidence on whether such a passage should be considered a conversion when unplanned. No study assesses the impact of these events on intraoperative and postoperative outcomes. However, according to experts' opinion, the switch from a robotic to a laparoscopic procedure shouldn’t impact postoperative morbidity as both the robot-assisted and laparoscopic procedures are proven to offer similar benefits in terms of surgical outcomes.
Proposed action: Switching to an alternative minimally invasive approach during pancreatic surgery should be reported in surgical series. Future studies are needed to investigate the impact of conversion from a minimally invasive approach to another.

**L4. Do all conversions to open in laparoscopic surgery (LS) have the same impact on patients’ outcome?**Recommendation: In laparoscopic pancreatic surgery, urgent conversions are usually associated with an adverse impact on patients’ outcomes compared to non-urgent conversions. An effort should be made to perform an elective conversion prior to getting into an emergency conversion (GRADE 1C, expert agreement 97.3% , audience agreement 93.3%).

**R4. Do all conversions to open in robot-assisted surgery (RAS) have the same impact on patients’ outcome?**Recommendation: In robot-assisted pancreatic surgery, urgent conversions are usually associated with an adverse impact on patients’ outcomes compared to non-urgent conversions. An effort should be made to perform an elective conversion prior to getting into an emergency conversion (GRADE 1C, expert agreement 97.3% , audience agreement 96.5%).
Comments L4-R4: Only two studies investigated the impact of urgent and non-urgent conversion in minimally invasive (either laparoscopic or robotic) pancreatic resections.^2, 3^ For urgent conversion only, these studies show increased intraoperative blood loss, need for transfusions, and postoperative complications. However, both studies are limited by the small numbers and therefore not powered to demonstrate an impact on mortality and postoperative complications with low incidence. The IHPBA guidelines on terminology for MIPS suggested to classify conversion as “urgent/reactive” or “non-urgent/conditional”.^1^
Proposed Action L4-R4: The intraoperative reason for an unplanned conversion should be specified in all MIPS series and classified into urgent and non-urgent. Large prospective studies are needed to define the impact of such events on severe postoperative complications and mortality.

**L5. How should we define a non-urgent conversion in LS?**Recommendation: In laparoscopic pancreatic surgery, a “non-urgent conversion” is a conversion to laparotomy for unexpected conditions (i.g. tumor extension/adhesions to adjacent organs/equipment failure) but not in an emergency setting. During the conversion phase, the patient's vital parameters are stable and there is no active bleeding (GRADE 1C, expert agreement 91.9% , audience agreement 98.2%).

**R5. How should we define a non-urgent conversion in RAS?**Recommendation: In robot-assisted pancreatic surgery, a “non-urgent conversion” is a conversion to laparotomy for unexpected conditions (i.g. tumor extension/adhesions to adjacent organs/equipment failure) but not in an emergency setting. During the conversion phase, the patient's vital parameters are stable and there is no active bleeding (GRADE 1C, expert agreement 91.9% , audience agreement 99.1%).

**L6. How should we define an urgent conversion in LS?**Recommendation: In laparoscopic pancreatic surgery, an “urgent conversion” is an unplanned conversion for unexpected potentially life-threatening conditions such as bleeding or other conditions affecting patients’ vital parameters (GRADE 1C, expert agreement 89.2% , audience agreement 99.1%).

**R6. How should we define an urgent conversion in RS?**Recommendation: In robot-assisted pancreatic surgery, an “urgent conversion” is an unplanned conversion for unexpected potentially life-threatening conditions such as bleeding or other conditions affecting patients’ vital parameters (GRADE 1C, expert agreement 91.9% , audience agreement 97.0%).
Comments L5-R6: The IHPBA guidelines for terminology in MIPS classify conversion from MIPS to open as “conditional or non-urgent” and “reactive or urgent”.^1^ The first term should be used for conversion secondary to tumor infiltration to other organs, difficult in exposure or failure to progress, whereas the term “reactive or urgent” conversion should be used in case of intraoperative bleeding or organ perforation. In two small retrospective studies,^2, 3^ conversion from minimally invasive to open surgery has been classified as elective conversion when performed because of vascular involvement by tumor, oncological concerns, adhesions, or technical difficulties and as emergency conversion, when performed because of bleeding. Based on preliminary data, the different types of conversions seem to be associated with different outcomes.
Proposed action L5-R6: Conversion from laparoscopy/robot-assisted to open surgery should be classified as “non-urgent” or “urgent” conversion in all surgical series. Prospective studies are needed to define the impact of such events on severe postoperative complications and mortality.

**L7. How should we define an unintended conversion in LS (i.g. gastrojejunostomy performed open, even though it was initially planned laparoscopically)?**Recommendation: In laparoscopic pancreatic surgery, the unplanned use of a laparotomy to complete the procedure must be defined as a non-urgent conversion (GRADE 1 EXPERT OPINION, expert agreement 83.8% , audience agreement 89.3%).

**R7. How should we define an unintended conversion in RAS (i.g. gastrojejunostomy performed open, even though it was initially planned laparoscopically)?**Recommendation: In robot-assisted pancreatic surgery, the unplanned use of a laparotomy to complete the procedure must be defined as a non-urgent conversion (GRADE 1 EXPERT OPINION, expert agreement 83.8% , audience agreement 83.9%).
Comments L7-R7: Several studies reported on performing one or more anastomoses using an open approach through the incision used for specimen extraction^4-16^. This technique is mostly reported by surgeons in the initial phase of the learning curve toward a pure-robotic approach. At this stage of the learning curve, this technique is safe and has been proven to have comparable intraoperative and postoperative outcomes compared to a total minimally invasive procedure. However, data on the unplanned use of these techniques are lacking and it should therefore be considered a non-urgent conversion.
Proposed action L7-R7: The unplanned use of an open technique to perform an anastomosis should be reported in surgical series to further investigate its impact on postoperative outcomes.

**DOMAIN 2. INDICATIONS***Topic 3: Benign and pre-malignant lesions Quality score: 76%*
**L8.** **What is the role of LS in the management of benign or pre-malignant lesions in the pancreatic body and tail?**Recommendation: Laparoscopic distal pancreatectomy (LDP) should be considered over the traditional open approach in the management of benign or pre-malignant lesions in the pancreatic body and tail, when performed by experienced surgeons in high-volume centers. It is associated with a shorter time to functional recovery, shorter hospital stay, and reduced blood loss with similar complication and CR-POPF rates compared to open distal pancreatectomy (GRADE 1A, expert agreement 94.6% , audience agreement 94.4%).
Comments: 66 comparative studies have been published comparing LDP with ODP, including two randomized controlled trials^17, 18^. These trials have shown reduced time to functional recovery, a shorter hospital stay and less bleeding after LDP with comparable morbidity and mortality rates as compared to ODP. Therefore, the previous Miami guidelines have recommended to prefer LDP over ODP for benign and low malignant lesions.^19^

**R8. What is the role of RAS in the management of benign or pre-malignant lesions in the pancreatic body and tail?**Recommendation R8.1: Robot-assisted distal pancreatectomy (RDP) should be considered as a valid approach in the management of benign or pre-malignant lesions in the pancreatic body and tail compared to laparoscopic or open distal pancreatectomy, when performed by experienced surgeons in high-volume centers (GRADE 1B, expert agreement 89.2% , audience agreement 91.4%).
Recommendation R8.2: RDP is comparable to LDP in terms of splenic preservation and early postoperative results, when performed by experienced surgeons in high-volume centers (GRADE 1B, expert agreement 83.8% , audience agreement 86.4%).

Comments: There are 7 cohort studies and 1 systemic review and meta-analysis comparing RDP with LDP for benign and low-malignant lesions.^20-27^ There are many more comparative studies on RDP versus LDP, but they include all indications. RDP seems associated with similar postoperative morbidity rates compared to LDP and ODP and is assumed to offer benefits in splenic preservation, conversion and intraoperative blood loss.
Proposed action L8-R8: Current evidence does not support a clear general definition of benign or low-grade malignant lesions; future consensus on this topic must be considered.

**L9.** **What is the role of LS in the management of benign or pre-malignant lesions in the pancreatic head?**

Recommendation: Laparoscopic pancreatoduodenectomy (LPD) can be considered as an alternative approach to open pancreatoduodenectomy (OPD) in the management of benign or pre-malignant lesions in the pancreatic head, when performed by experienced surgeons in high-volume centers (GRADE 2C, expert agreement 94.6% , audience agreement 93.9%).

Comments: There are 2 comparative cohort studies analyzing a small number of LPDs for pancreatic neuroendocrine tumors.^28, 29^ Preliminary data showed that LPD and OPD have a comparable R0 resection rate, 5-years recurrence-free, and overall survival. Data on the adequacy of lymphadenectomy are contradictory.

**R9. What is the role of RAS in the management of benign or pre-malignant lesions in the pancreatic head?**

Recommendation: Robot-assisted pancreatoduodenectomy (RPD) can be considered as an alternative approach to open pancreatoduodenectomy (OPD) in the management of benign or pre-malignant lesions in the pancreatic head, when performed by experienced surgeons in high-volume centers (GRADE 2C, expert agreement 86.5% , audience agreement 96.2%).

Comments: Only 1 comparative cohort study reports on the outcomes of RPD in the treatment of pancreatic neuroendocrine tumors.^29^ Compared to OPD, RPD is suggested to allow for a higher number of harvested lymph nodes, along with a similar rate of R0 resections, 5-years recurrence-free and overall survival.^29^
Proposed action L9-R9: Large prospective studies with longer follow up are needed on this topic.

*Topic 4: Pancreatic ductal adenocarcinoma Quality score: 79%*

**L10.** **What is the role of LS in the management of PDAC in the pancreatic body and tail?**

Recommendation: Laparoscopic left radical pancreatectomy should be considered as an alternative approach in the management of resectable PDAC in the pancreatic body and tail, when performed by experienced surgeons in high volume centers (GRADE 2B, expert agreement 94.6% , audience agreement 86.8%).

Comments: There are 34 comparative studies on LDP versus ODP for PDAC, of which 5 systematic reviews. Its feasibility, safety and oncological efficiency is similar to open distal pancreatectomy. The two most recent systematic reviews showed comparable survival and R0 resection rates of MIDP (mainly laparoscopic procedures) as compared to ODP but a lower lymph node retrieval in MIDP.^30, 31^ The results of the randomized DIPLOMA-trial (LDP versus ODP for PDAC, NCT04483726) are expected soon and could therefore not be included in the current guidelines. No evidence is available on the role of LDP in locally advanced PDAC or after neo-adjuvant treatment.

**R10.** **What is the role of RAS in the management of PDAC in the pancreatic body and tail?**

Recommendation: Robot-assisted left radical pancreatectomy should be considered as an alternative approach in the management of resectable PDAC in the pancreatic body and tail, when performed by experienced surgeons in high-volume centers (GRADE 2B, expert agreement 86.5% , audience agreement 86.7%).

Comments: There are 3 comparative studies^32-34^ and 1 recent systematic review^35^ on RDP versus LDP in PDAC only, who demonstrate similar oncological outcomes of RDP compared to LDP. Moreover, RDP seems to allow for a higher R0 resection rate and lymph node yield. A systematic review from Chopra et al.,^36^ that compared RDP with LDP with ODP, found similar results and concluded a non-inferiority of RDP compared to LDP and ODP. 2 retrospective cohort studies comparing RDP with ODP^37, 38^ reported comparable oncological outcomes, with 1 of them showing a significant improved survival in the RDP group.^38^ No evidence is available on the role of RDP in locally advanced PDAC or after neo-adjuvant treatment.

Proposed action L10-R10: Large prospective studies and randomized controlled trials are needed on this topic.

**L11. What is the role of LS in the management of PDAC in the pancreatic head?**

Recommendation: LPD can be considered as an alternative approach in the management of PDAC in the pancreatic head, when performed by experienced surgeons in high-volume centers. Surgeons performing LPD must participate in a registry or follow their outcomes in a prospectively maintained database (GRADE 2A, expert agreement 89.2% , audience agreement 90.7%).

Comments: Since 2018, there are 3 large retrospective cohort studies^39-41^, 2 propensity score matched cohort studies^42, 43^, 4 systematic reviews and meta-analyses who focus selectively on the comparison of LPD and OPD in PDAC.^44-47^ The largest cohort study^39^ reported a non-significant difference in survival time between both two groups (20.7 vs. 20.9 months). However, Chapman et al.^40^ compared survival data from the NCDB, and found a significant difference in median overall survival between LPD and OPD of 19.8 versus 15.6 months, respectively. All of the 4 meta-analyses^44-47^ showed that LPD for PDAC is non-inferior to OPD. The included comparative studies were not randomized and therefore a certain risk on bias is present. No evidence is available on the role of LPD in locally advanced PDAC or after neo-adjuvant treatment.
Proposed action: Future studies should focus on long-term oncological outcomes.

**R11. What is the role of RAS in the management of PDAC in the pancreatic head?**
Recommendation: RPD can be considered as an alternative approach in the management of PDAC in the pancreatic head, when performed by experienced surgeons in high-volume centers. Surgeons performing RPD must participate in a registry or follow their outcomes in a prospectively maintained database (GRADE 2C, expert agreement 89.2% , audience agreement 89.1%).
Comments: Since 2016, 1 large retrospective cohort study and 2 propensity score matched analyses on RDP versus LDP for PDAC have been published.^43, 48, 49^ All studies showed comparable short-term outcomes of RPD compared to ODP and 2 studies^48, 49^ showed comparable survival as well. None of the available studies were randomized and do therefore introduce a risk on bias. Also, no systematic review and meta-analyses are available on this topic yet. No evidence is available on the role of the RPD in locally advanced PDAC or after neo-adjuvant treatment.
Proposed action: Future research on the role of RPD compared to both LPD and OPD on this topic are needed.

*Topic 5: Periampullary adenocarcinoma Quality score: 73%*

**L12.** **What is the role of LS in the management of non-pancreatic periampullary adenocarcinoma (NPPC)? (ampullary, duodenal and cholangiocarcinoma)**Recommendation: LPD is an acceptable alternative for patients with a resectable non-pancreatic periampullary adenocarcinoma (NPPC), when performed by experienced surgeons in high-volume centers. Surgeons performing LPD must participate in a registry or follow their outcomes in a prospectively maintained database (GRADE 2C, expert agreement 81.1% , audience agreement 96.4%).
Comments: There are no meta-analyses selectively focusing on NPPC. One meta-analysis^50^ performed subgroup analyses with studies^51, 52^ selectively focusing on the indication NPPC. One comparative study^52^ and one propensity score matched study^53^ focused on NPPC selectively, but both did not perform a subgroup analysis for different NPPC’s. All studies are coherent in their outcomes and show that LPD is not inferior to OPD and that there are potential short-term benefits in terms of less blood-loss and shorter length of stay. These studies support the evidence already established in studies comparing LPD versus OPD for all indications. However, studies focusing selectively on one of the periampullary tumors are scarce and long-term results are lacking.

**R12. What is the role of RAS in the management of non-pancreatic periampullary adenocarcinoma (NPPC)? (ampullary, duodenal and cholangiocarcinoma)**Recommendation: RPD is an acceptable alternative for patients with a resectable non-pancreatic periampullary adenocarcinoma (NPPC), when performed by experienced surgeons in high-volume centers. Surgeons performing RPD must participate in a registry or follow their outcomes in a prospectively maintained database (GRADE 2C, expert agreement 91.9% , audience agreement 93.0%).
Comments: There are no comparative cohort studies or meta-analyses comparing RPD with LPD or OPD for ampullary, distal cholangio or duodenum adenocarcinoma specifically. In addition, there are no reviews assessing the role of RPD with subgroup analyses for the different NPPC’s. RPD is assumed to be safe and feasible for PDAC or all periampullary tumors collectively. Based on these studies, one could assume that RPD is safe in short-term outcomes. However, as data on these subgroups is lacking, the impact of RPD in short- and long-term outcomes for patients with NPPC should still be further evaluated.
Proposed action L12-R12: Future research on the role of RPD and LPD on this topic are needed with the important addition of stratification for different periampullary tumors.

**DOMAIN 3. PATIENTS SELECTION**
*Topic 6: High-risk patients Quality score: 78%***L13. Are there any contraindications for laparoscopic pancreatic resections (LPR) related to; (13.1) patients age, (13.2) obesity, (13.3) previous abdominal surgery?**Recommendation L13.1: Age alone should not be a contra-indication for LPD and LDP. LPD and LDP are appropriate alternatives (GRADE 1B, expert agreement 100%, audience agreement 93.2%).
Recommendation L13.2: Obesity alone should not be considered as a contraindication for performing LDP or LPD. However, obesity is a risk factor for postoperative complications (especially POPF) (GRADE 2C, expert agreement 89.2%, audience agreement 91.2%).
Recommendation L13.3: Previous abdominal surgery alone should not be considered as an absolute contraindication for LDP/LPD (GRADE 2C, expert agreement 94.6%, audience agreement 96.0%).
Comments: (13.1) Outcomes in elderly patients between LPD and ODP and LDP and ODP are comparable.^54-56^ (13.2) No evidence exists to assume that obesity must be considered a contraindication for performing LDP or LPD. However, studies have identified BMI as a risk factor for postoperative complications.^57-59^ (13.3) Limited data on previous abdominal surgery in LPR are available. The available studies do not report any contraindication for LPR in patients with previous abdominal surgery. However, a retrospective single center study reported that previous abdominal surgery might increase the severity of postoperative complications.^60^

**R13. Are there any contraindications for robot-assisted pancreatic resections (RPR) related to; (13.1) patients age, (13.2) obesity, (13.3) previous abdominal surgery?**

Recommendation R13.1: Age alone should not be a contra-indication for RPD. RPD is an appropriate alternative for elderly patients (GRADE 2C, expert agreement 97.3%, audience agreement 93.3%).

Recommendation R13.2: Obesity alone should not be considered as a contraindication for performing RDP or RPD. However, obesity is a risk factor for postoperative complications (especially POPF). (GRADE 2C, expert agreement 83.8%, audience agreement 95.3%).

Recommendation R13.3: Previous abdominal surgery alone should not be considered as an absolute contraindication for RDP/RPD. (GRADE 2C, expert agreement 86.5%, audience agreement 91.5%).

Comments: (13.1) Only 2 retrospective studies^61, 62^ and 1 systematic review^63^ are available on outcomes of RPD in elderly patients. The systematic review and meta-analysis showed that MIPD is a safe and feasible procedure for select elderly patients if performed by experienced surgeons from high-volume pancreatic centers. Data on the outcomes of RDP in elderly patients are lacking. (13.2) There are 4 retrospective studies available on the use of RPD in obese patients.^57, 58, 64, 65^ RPD might be associated with a shorter operation time, less blood loss and a lower POPF rate compared to OPD. Limited evidence is available on the use of RDP in obese patients.^66^ A higher BMI might be a predictor for postoperative overall complications (especially POPF) after RDP.^57, 66^ (13.3) Only one study reported on previous abdominal surgery in RPR.^67^ It suggested that RPR are safe and feasible in patients with a history of minor prior abdominal surgery.
Proposed action L13-R13: Future studies to the role of RDP in elderly and obese patients are required. Future studies should also compare RPR with LPR in obese patients in terms of postoperative morbidity. Definitions of elderly, obesity, and previous abdominal surgery are required.

**L14. Are LPR an appropriate alternative in patients with large lesions?**

Recommendation: Size of the lesion alone is not a contraindication for LDP (GRADE 2C, expert agreement 94.6%, audience agreement 94.6%).

**R14. Are RPR an appropriate alternative in patients with large lesions?**

Recommendation: Size of the lesion alone is not a contraindication for RDP (GRADE 2C, expert agreement 94.6%, audience agreement 92.9%).

Comments L14-R14: A propensity-scored matched study showed that in large tumors (>5cm) RDP offers benefits in terms of conversion and spleen-preserving rates as compared to LDP.^68^ No evidence is available on RPD and LPD.
Proposed action L14-R14: Future studies on this topic are required.

**L15. Are LPR an appropriate alternative in patients with vascular involvement?**

Recommendation: Scarce evidence exists regarding the use of vascular resection in LPR. LPD with vascular resection should only be performed by highly experienced surgeons and in high-volume centers. Further investigation is warranted on this topic and data on patient treatment and outcomes needs to be entered in prospective registries and databases (GRADE 2C, expert agreement 100.0%, audience agreement 91.2%).

Comments: Only 3 retrospective studies are available on vascular involvement in LPD.^69-71^ One study concluded that vascular resection in LPD is feasible and safe as it is associated with similar morbidity and mortality compared to OPD.^70^ No evidence is available on LDP.

**R15.** **Are RPR an appropriate alternative in patients with vascular involvement?**

Recommendation: No evidence exists regarding the use of vascular resection in RPR. RPD with vascular resection should only be performed by highly experienced surgeons and in high-volume centers. Further investigation is warranted on this topic and data on patient treatment and outcomes needs to be entered in prospective registries and databases (GRADE 2C, expert agreement 100.0%, audience agreement 94.6%).

Comments: No evidence exists on RPR in patients with vascular involvement.
Proposed action L15-R15: Future investigation on this topic is warranted.

**L16. Are LPR indicated for the treatment of PDAC after neoadjuvant therapy?**

Recommendation: There is no data available regarding LPD/LDP after neoadjuvant therapy, further investigation is warranted (GRADE 2C, expert agreement 100%, audience agreement 92.4%).

**R16. Are RPR indicated for the treatment of PDAC after neoadjuvant therapy?**Recommendation: There is no data available regarding RPD/RDP after neoadjuvant therapy, further investigation is warranted (GRADE 2C, expert agreement 100%, audience agreement 92.7%).

Comments L16-R16: There is no evidence available on this topic. Results of two randomized controlled trials are expected (NCT04855331 and ChiCTR2000029987).

Proposed action L16-R16: Further studies in this area are required.

**DOMAIN 4. PROCEDURES**
*Topic 7: Pancreatoduodenectomy Quality score: 66%***L17.** **What is the preferred anastomosis technique in LPD?**Recommendation: There is insufficient evidence to define a superior anastomotic technique during LPD. The choice of anastomosis during LPD is the surgeon’s preference (GRADE 2 EXPERT OPINION, expert agreement 100%, audience agreement 90.8%).
Comments: In total, there are 3 comparative studies focusing on different anastomotic techniques of the pancreatojejunostomy in LPD. ^72-74^ All included a low number of patients. Due to the low number of cases, none of the studies could demonstrate significant superior results. In addition, the groups of the studies varied widely. Therefore, a preferable technique for laparoscopic anastomosis could not be supported.

**R17. What is the preferred anastomosis technique in RPD?**Recommendation: There is insufficient evidence to define a superior anastomotic technique during RPD. The choice of anastomosis during RPD is the surgeon’s preference (GRADE 2 EXPERT OPINION, expert agreement 97.3%, audience agreement 92.9%)
Comments: There are no high-evidence studies available that compared different anastomotic techniques in RPD. One retrospective cohort study compared the modified Blumgart in RPD and OPD and found comparable results.^75^ No studies have been performed on robotic pancreato-gastrostomy, gastrojejunostomy or hepato-jejunostomy. Therefore, it is not possible to define a preferable robotic anastomosis technique.
Proposed action: Future studies are required.

*Topic 8: Distal Pancreatectomy Quality score: 80%* **L18.** **What are the recommendations on LS for the different spleen-preserving techniques?**Recommendation: In laparoscopic spleen preserving distal pancreatectomy, both vessel-sparing and vessel-resecting techniques are appropriate alternatives for the treatment of benign and pre-malignant diseases. (GRADE 1C, expert agreement 97.3%, audience agreement 90.8%).

**R18. What are the recommendations on RAS for the different spleen-preserving techniques?**Recommendation: In robot-assisted spleen preserving distal pancreatectomy, both vessel-sparing and vessel-resecting techniques are appropriate alternatives for the treatment of benign and pre-malignant diseases (GRADE 1C, expert agreement 97.3%, audience agreement 90.6%)
Comments L18-R18: Available literature mainly consists of studies comparing the different spleen-preserving techniques but without comparing both techniques in RAS and LS groups. According to two meta-analyses, the Kimura technique should be chosen above the Warshaw technique due to lower splenic infarction, varices and CVIII-V complications.^25, 76^ However, evidence on the outcomes of different spleen-preserving techniques in RAS is still lacking. The highest level of evidence on both spleen-preserving techniques in LS and RAS are 2 retrospective mono-center cohort studies that compared both techniques between RDP and LDP. They concluded that robot-assisted spleen preserving distal pancreatectomy leads to a higher success rate of spleen-vessel preservation as compared to laparoscopic spleen preserving distal pancreatectomy. ^77, 78^
Proposed action: Future studies should focus on the role and potential benefits of a robot-assisted or laparoscopic approach in both spleen-preserving techniques.

*Topic 9: Parenchymal-sparing Quality score: 74%***L19. What is the role of LS in central pancreatectomy, regardless of indication?**Recommendation: The role of LS in central pancreatectomy has yet to be determined. Future studies are recommended (GRADE 1C, expert agreement 100%, audience agreement 84.3%)
Comments: The evidence on laparoscopic central pancreatectomy is based on 3 case-control studies^79-81^ and 1 case-serie.^82^ All these studies have a low level of evidence as they are retrospective, non-matched studies with a high risk of selection bias; hence, no firm conclusions can be drawn.

**R19. What is the role of RAS in central pancreatectomy, regardless of indication?**Recommendation: The role of RAS in central pancreatectomy has yet to be determined. Future studies are recommended (GRADE 1C, expert agreement 97.3%, audience agreement 86.8%)
Comments: A Chinese RCT showed that robot-assisted central pancreatectomy (RCP) was associated with shorter length of stay, reduced operative time, blood loss and clinical POPF rate, and expedited postoperative recovery, compared to open central pancreatectomy (OCP).^83^ Besides this RCT, a recently published meta-analysis of 12 case series reported an average rate of POPF of 42.3% after RCP (37.4% for PJ, 36.5% for PG, and 53.4% for end-to-end anastomosis between the two pancreatic stumps)^84^. Despite this promising results, the role of RCP still needs to be further determined.
Proposed action: Central pancreatectomy is a relatively novel procedure that first needs to be further implemented and investigated before conclusions about its safety and feasibility can be drawn.

**L20. What is the role of LS in enucleation?**Recommendation: Laparoscopic enucleation of pancreatic lesions in selected patients should be considered as an appropriate alternative to open enucleation (GRADE 1B, expert agreement 94.6%, audience agreement 86.4%).

**R20. What is the role of RAS in enucleation?**Recommendation: Robot-assisted enucleation of pancreatic lesions in selected patients should be considered as an appropriate alternative to open enucleation (GRADE 1B, expert agreement 94.6%, audience agreement 81.8%).
Comments L20-R20: Most of the evidence on minimally invasive enucleation (MI-En) does not analyze the laparoscopic and robotic techniques separately. Two meta-analyses showed that MI-En was associated with a shorter operative time and hospital stay with comparable postoperative morbidity as compared to open enucleation (O-En)^85, 86^ However, all the included studies were retrospective studies with a high risk of selection bias and in only 1 study matching was performed. Another large systematic review including nearly 1000 patients undergoing pancreatic enucleation reported a reduced rate of POPF and overall morbidity in patients who received a minimally invasive approach. However, all included studies were also case series with a low quality of evidence. ^87^ The advantages of MI-En in terms of reduced blood loss, operative time and LOS with similar morbidity compared with O-En are confirmed in other propensity score-matched studies^88, 89^.
Proposed action L20-R20: Future studies should analyze robot-assisted and laparoscopic enucleations separately.

*Topic 10: Total pancreatectomy Quality score: 76%* **L21. What is the role of LS in total pancreatectomy, taking into account different indications?**
Recommendation: Laparoscopic total pancreatectomy is an alternative approach to open total pancreatectomy when performed in selected patients by experienced surgeons in high volume centers (GRADE 2C, expert agreement 97.3%, audience agreement 93.3%).
Comments: 3 case-series and 2 case-control studies explored the role of laparoscopy in total pancreatectomy.^90-94^ They suggested that laparoscopic total pancreatectomy (LTP) is safe and feasible when performed in selected patients at the high-volume centers. According to the data from the American National Cancer Database, LTP is associated with shorter hospital stay, lower 30- and 90-day mortality as compared with open total pancreatectomy (OTP), while achieving similar oncologic outcomes.^94^

**R21. What is the role of RAS in total pancreatectomy, taking into account different indications?**Recommendation: Robot-assisted total pancreatectomy is an alternative approach to open total pancreatectomy when performed in selected patients by experienced surgeons in high volume centers (GRADE 2C, expert agreement 97.3%, audience agreement 91.7%).
Comments: Robot-assisted total pancreatectomy (RTP) was studied in 2 case-series and 5 case-control studies, with some of them including propensity-score matching. ^75, 94-99^ Conflicting results were reported for operative time, but less intraoperative blood loss, faster postoperative recovery and shorter hospital stay were found after RTP when compared with OTP.^75, 97-99^ The analysis of the American nationwide data suggested reduced 30- and 90-day mortality after RTP compared to OTP^94^. Furthermore, 1 study demonstrated longer overall and cancer-specific survival in patients with pancreatic cancer and malignant IPMN.^98^
Proposed action L21-R21. Prospective database studies and registries are encouraged to improve the level of evidence on these topics.

**DOMAIN 5. SURGICAL TECHNIQUES AND INSTRUMENTATION**
*Topic 11: Techniques in Pancreatoduodenectomy Quality score: 49%***G22. What are the anatomical landlines when performing a minimally invasive Kocher Maneuver?**Recommendation G22.1: For the safe completion of the Kocher maneuver during MIPS, it is advised to follow these landmarks: (a) Medial edge: exposure of the inferior vena cava (up to the right edge of the aorta) to identify the left renal vein and the origin of the superior mesenteric artery. (b) Anterior edge: entire visualization of the entire posterior surface of the head of the pancreas. (c) Inferior edge: mobilization of the duodenum from the transverse mesocolon up to the right margin of the ligament of Treitz beneath the superior mesenteric vessels. (d) Superior edge: hepatic caudate lobe (GRADE 2 EXPERT OPINION, expert agreement 97.3%, audience agreement 95.6%).
Recommendation G22.2: To safely accomplish specific artery first approaches and venous vascular control during MIPS, a wider mobilization to expose the SMA may be necessary (GRADE 2 EXPERT OPINION, expert agreement 83.8%, audience agreement 95.6%).
Comments: Method sections usually describe the Kocher maneuver as one of the key steps of a MIPD. Anatomical landmarks are rarely mentioned in the literature, but when they are, they are consistently reported. In some cases, such as artery-first approaches and venous vascular involvement, an extended Kocher maneuver has been recommended.

**G23. Is there a specific indication toward the artery first approach in minimally invasive pancreatoduodenectomy (MIPD)?**Recommendation G23.1: An artery first approach is feasible during MIPD. The indications between MIPD and OPD are the same (GRADE 1C, expert agreement 97.3%, audience agreement 98.2%).
Recommendation G23.2: The artery first approach during MIPD should be tailored on a case-by-case basis. Surgeons should be aware of each approach (anterior, posterior, left, right, and combined) to SMA dissection keeping in mind that the right SMA approach could be appropriate but may reveal limitations in specific patients in which combined approaches are recommended (GRADE 2C, expert agreement 97.3%, audience agreement 93.3%).
Comments: A total of 27 papers investigated the artery-first approach in MIPD, including 12 comparative studies, 8 case series, 4 case reports, and 3 narrative reviews. Four reports used a robotic-assisted approach. The SMA approaches were mainly categorized into 4 categories according to the direction toward the SMA when initiating the dissection around the SMA: anterior (2 studies^69, 100^), posterior (6 studies^100-105^), left (3 studies^100, 106, 107^), and right (16 studies^100, 103, 108-121^) approach, the latter being the most frequently reported. One case-control study reported three different SMA-first approaches suggesting each one on a case-by-case basis.^100^ Most studies assessed the feasibility and safety of an SMA-first approach in MIPD at high-volume centers experienced in pancreatic and MI surgery.

**G24. At what stage should the pancreatic parenchyma be divided?**Recommendation G24.1: Standardization of the timing of surgical steps, including pancreatic transection, to safely perform MIPD is recommended when possible (GRADE 2C, expert agreement 91.9%, audience agreement 92.0%).
Recommendation G24.2: Dividing the pancreas after a broad dissection from the portal-mesenteric axis at both the upper and lower edges of the pancreatic neck and possibly completing a retro pancreatic tunnel and a broad Kocher maneuver is advisable during MIPD (GRADE 2C, expert agreement 91.9%, audience agreement 85.6%).
Recommendation G24.3: In MIPD, the pancreatic neck is preferentially divided from the inferior to the superior margin. This approach leads to the identification of the main pancreatic duct, which could be selectively divided with cold scissors (GRADE 1C, expert agreement 94.6%, audience agreement 98.2%).
Comments: No study explicitly investigates the best timing for pancreatic transection during a MIPD, but method sections usually describe the pancreatic transection as a critical standardized step whose timing is based on institutional policies. Surgical principles of proper surgical field view before pancreatic transection were always recommended. Most reports approached pancreatic division from the inferior to the superior pancreatic isthmic margin with a separate transection of the main pancreatic duct with cold scissors.^111, 122-124^

**G25. Are there any benefits or specific indications for the biliary tree's early or delayed division?**Recommendation: In MIPD, biliary duct division is performed after clear visualization of the pertinent vascular anatomy including aberrant arteries. The timing of the division is the surgeons’ preference (GRADE 2C, expert agreement 81.1%, audience agreement 83.2%).
Comments: Studies investigating the best timing for biliary duct division during MIPD are lacking. In most reports, the biliary division has been reported as an intermediate step during MIPD. Surgical principles of proper surgical field view before biliary division were always recommended, specifically facing aberrant vascular and biliary anatomy.
Proposed action: Further studies are needed to define the influence of timing in the biliary division in preventing or worsening infectious complications after a MIPD.

*Topic 12: Techniques in Distal Pancreatectomy Quality score: 50%* **G26. What is the best approach for dissection/control of the splenic vessels?**Recommendation G26.1: When appropriate, dissection between the pancreas and splenic vessels should be carefully performed with a combination of blunt dissection and energy devices after complete mobilization of the colonic splenic flexure (GRADE 1C, expert agreement 81.1%, audience agreement 85.1%).
Recommendation G26.2: Careful attention should be given to control small arterial and venous branches into the pancreas (with clips and/or energy devices) when splenic vessels need to be preserved (GRADE 1C, expert agreement 94.6%, audience agreement 94.7%).
Recommendation G26.3: A tailored approach to the splenic artery should be encouraged according to individual cases and vascular anatomy. Surgeons should be familiar with both the anterior and posterior approaches (GRADE 1C, expert agreement 97.3%, audience agreement 93.4%).
Recommendation G26.4: When dividing the pancreas at the level of the neck, clear visualization of the splenic/portal vein junction should be obtained prior to ligation and division of the splenic vein. When dividing the pancreas to the left of the celiac trunk, the splenic vessels could be individually ligated or incorporated in the pancreatic division according to surgeon preference (GRADE 1C, expert agreement 83.8%, audience agreement 90.6%).
Recommendation G26.5: Accurate preoperative planning and revision of imaging is recommended to evaluate the patient's arterial and venous vascular anatomy to safely approach splenic vessels (GRADE 1C, expert agreement 97.3%, audience agreement 97.9%).
Comments: Retrospective studies, case-control studies, and technical notes investigating splenic vessels dissection described individual centers/surgeons' practices highlighting approach heterogeneity.^125-128^ [26.2-5] A clear visualization of splenic vessels is recommended in all case series, and a systematic review advocated for an individualized approach influenced mainly by tumour location and vascular anatomy.^129^ [47.2] Most reports on spleen-preserving DP suggested precise vascular dissection of the small tributaries of the splenic artery and vein.^125, 126, 128^ [27.3] Shorter operative times and lower estimated blood loss^130^ were described once the splenic artery (SA) was dissected with the anterior approach, but the SA patterns and variations may alter the final planning.^129^ [27.4] A non-inferiority RCT investigated the combined division of the splenic vein (SV) and pancreatic parenchyma to the left of the celiac trunk with a Tri-Staple Reinforced Reload, observing non-inferior POPF rates compared to a separated division.^127^ As specific intraoperative conditions can alter the safety of a combined division, despite the lack of evidence, an agreement has been obtained for a pancreatic division according to surgeon preference.

**G27. Is there any indication for a pancreatic hanging maneuver in minimally invasive distal pancreatectomy (MIDP)?**Recommendation: The pancreatic hanging maneuver is an appropriate option during MIDP (GRADE 1C, expert agreement 83.8%, audience agreement 90.4%).
Comments: Two studies were considered.^131, 132^ Experts describe a pancreatic hanging maneuver as a standard practice to ensure good surgical field exposure and vascular control with suitable tensions.
Proposed action: Prospective studies and RCTs are needed to expand the knowledge on this topic.

*Topic 13: Surgical devices Quality score: 55%***G28. What type of energy and instruments should be used during the dissection phase?**Recommendation: The choice of energy devices and instruments for dissection during MIPS should be based upon surgeons' preferences (GRADE 1C, expert agreement 100.0%, audience agreement 96.7%).
Comments: 3 RCTs and 6 case-control studies examined surgical devices used for pancreatic dissection.^125, 133-140^ Of these, only one addressed the use of these devices in MIPS, comparing the outcomes for LigaSure with those for ultrasonic shears and endo-clips.^125^ This study suggests that using LigaSure in laparoscopic spleen-preserving distal pancreatectomy results in shorter operative time, less blood loss, better splenic vein patency and shorter hospital stay. Three RCTs found no differences in clinical outcomes when comparing LigaSure, ultrasonic dissection and conventional techniques in open pancreatoduodenectomy and total pancreatectomy.^133-135^ One of these studies reported increased costs for LigaSure compared with the conventional technique.^135^

**G29. What is the role of the hand-assisted technique for pancreatic resections?**Recommendation: There is a limited role for hand-assisted procedures in contemporary minimally invasive pancreatic surgical practice (GRADE 1C, expert agreement 89.2%, audience agreement 86.8%).
Comments: Several case reports and small series have explored the role of hand-assisted technique.^141-146^ These were published in the introduction phase of MIPS. The hand-assisted technique was considered when minimally invasive pancreatic resection could not be safely accomplished, like during the learning curve and complex procedures due to patient- and tumour-specific characteristics.

*Topic 14: Vessel and Hemorrhage control Quality score: 46%***G30. Is there any approach indicated when venous resections are considered during MIPD?**Recommendation G30.1: A careful expansion of selection criteria for MIPD to include major venous resections can be an option for highly experienced pancreatic surgeons in high-volume centers. Surgeons performing minimally invasive vascular resection should participate in a registry or have a prospectively maintained database to follow their outcomes (GRADE 2C, expert agreement 97.3%, audience agreement 94.0%).
Recommendation G30.2: Reserving the venous resection as the final step of a MIPD once dissection is completed and after correct exposure of the portal-mesenteric axis is recommended to minimize clamp time (GRADE 1C, expert agreement 97.3%, audience agreement 93.5%).
Comments: A total of 20 studies,^41, 69-71, 147-161^ including retrospective, case-control series and case reports, 2 systematic reviews^162, 163^ and 1 meta-analysis^164^ investigated venous vascular resections during MI pancreatic surgery. Seven reports used a robotic-assisted approach.^148, 149, 151, 152, 155, 158, 161^. All reports remarked that the role of MI surgery for pancreatic cancer with vascular involvement at the time of surgery is likely to expand but emphasized this implementation specifically by highly experienced pancreatic surgeons in high-volume centres.^153-155^ [30.2] Several papers suggested reserving the venous resection as the final step of a MIPD to limit the clamp time and ensure safe vascular exposure.^69, 71, 150, 153, 159^ Available data, despite limited, stems from high-volume institutional cohorts demonstrating similar operative and oncologic outcomes for MIS compared to standard open venous vascular resections.^41, 70, 153, 164^ Given the limited evidence, an agreement has been obtained for including all MIPD with vascular resection in registries, allowing precise implementation monitoring and constant procedure quality assessment.

**G31. Is there any approach indicated when arterial resections are considered during MIPS?**Recommendation: Arterial resection and/or reconstruction open or MI is not common practice. The MI approach for arterial resection/reconstruction or DP with coeliac axis resection can be performed by highly experienced pancreatic surgeons in carefully selected pancreas tumors. Surgeons performing minimally invasive vascular resection should participate in a registry or have a prospectively maintained database to follow and report their outcomes (GRADE 1C, expert agreement 94.6%, audience agreement 92.2%).
Comments: The literature review highlighted 9 manuscripts, including 6 case reports, 1 case series and 2 comparative studies dealing with MI arterial resections apart from the approach.^37, 151, 165-171^. The role of arterial resections in locally advanced pancreatic cancer remains controversial. Sporadic reports investigated the safety and feasibility of Distal Pancreatectomy with en-bloc Celiac Axis Resection (DP-CAR), both in the open or MI approach, although in small numbers and with limited long-term data on survival. Only 1 case-control study reported perioperative and oncologic outcomes of open and robotic DP-CAR at a high-volume pancreatic center, suggesting the feasibility and similar outcomes between the two approaches.^37^ Other data referred to arterial resection/reconstruction, or arterial repair, during MI pancreatic resections mainly required to manage an unexpected tumour infiltration or an intraoperative injury.^167^ Given the limited evidence, an agreement has been obtained to emphasize the importance of proper preoperative planning and patient selection besides including all MI arterial resections in registries, allowing precise implementation monitoring and constant procedure quality assessment.
Proposed action: Future registry-based studies should further investigate short- and long-term outcomes associated with MI vascular resections with pre-defined patient selection criteria.

**G32. What are the optimal techniques for control of hemorrhage during MIPS?**Recommendation G32.1: Of paramount importance in minimizing excessive blood loss during MIPS is optimizing prevention strategies by assuring adequate exposure, gentle dissection, and securing critical vessels (GRADE 1C, expert agreement 94.6%, audience agreement 92.0%).
Recommendation G32.2: Targeted interventions should be applied to treat intraoperative bleeding based on the extent and type of bleeding vessels. Bipolar cautery could be used to stop limited bleeding from small venous branches. Moderate venous bleeding can be temporally controlled by gauze compression and then by venous or arterial vessel clipping or suturing (GRADE 1C, expert agreement 97.3%, audience agreement 90.6%).

**G33. What are the optimal techniques for control of hemorrhage during MIDP with spleen preservation?**Recommendation G33.1: Proximal preparation and slinging of the splenic artery and vein before proceeding with pancreatic dissection is suggested during a Kimura's MI spleen preserving DP. This will allow their temporarily clamping in case of hemorrhage or definitive section (Warshaw’s MIDP/splenectomy) if hemostasis is not achieved (GRADE 1C, expert agreement 94.6%, audience agreement 93.4%).
Recommendation G33.2: Avoiding splenic injury is important during spleen preserving pancreatic resections. Surgeons should be familiar with best surgical practices to stop splenic bleeding (GRADE 1C, expert agreement 94.6%, audience agreement 94.9%).
Comments G32-G33: No studies specifically investigated optimal intraoperative hemorrhage control techniques during MIPS. Surgeries general principles include preventing and avoiding intraoperative bleeding by following the Halstedian surgical principles. Once vascular damage occurs, several techniques are used as a common practice to control bleeding based on the extent and type of bleeding vessels, confirming that surgeons can deal with specific bleeding complications still with the MI approach.^128^
Proposed action: Intraoperative bleeding complications should be consistently reported in the pancreatological literature. Specific intraoperative adverse events definitions and grading should be eventually developed for MI surgery to ensure outcomes and practice comparisons.

*Topic 15: Stump closure after Distal Pancreatectomy Quality score: 56%***G34. What are the technical details of pancreatic stump transection with staple devices indicated for the division of pancreatic parenchyma in MIDP?**Recommendation G34.1: In MIDP, a standardized technique for using a stapler to obtain adequate pancreatic stump compression is not available, although a gradual stepwise compression is advised (GRADE 1C, expert agreement 94.6%, audience agreement 96.8%).
Recommendation G34.2: The optimal choice of cartridges tailored to pancreatic parenchymal features is currently lacking and should be further investigated (GRADE 2C, expert agreement 94.6%, audience agreement 92.9%).
Comments: Two case-control studies and one retrospective case series^172-174^ examined peri-firing compression of the pancreatic stump before transection as a possible method for reducing the incidence of CR-POPF after distal pancreatectomy reporting lower incidence of CR-POPF after applying this technique.^173, 174^ One study reported only laparoscopic resections,^173^ while the other included both laparoscopic and open procedures.^174^ The retrospective cohort study reported no CR-POPF after using peri-firing compression in laparoscopic distal pancreatectomy.^172^ Four retrospective cohort studies have explored the possibility of optimal stapler cartridge selection for CR-POPF prevention in distal pancreatectomy.^175-178^ One of these included solely laparoscopic procedures, with no significant differences in CR-POPF when using white (2.5mm), blue (3.5mm) or green (4.1mm) Echelon 60 mm cartridges (Ethicon Endo-Surgery, Issy les Moulineaux, France). Three studies included open and minimally-invasive procedures reporting conflicting results on CR-POPF incidence for different stapler cartridges and their association with pancreatic thickness at the transection site.

**L35. Should staple versus another type of closure be used for the stump closure in LDP?**Recommendation: A stapling device can be considered for pancreatic stump closure in LDP. However, there are no clear advantages over other pancreatic stump closure techniques to prevent postoperative pancreatic fistula (GRADE 1B, expert agreement 100.0%, audience agreement 96.7%).

**R35. Should staple versus another type of closure be used for the stump closure in RDP?**Recommendation: A stapling device can be considered for pancreatic stump closure in RDP. However, there are no clear advantages over other pancreatic stump closure techniques to prevent postoperative pancreatic fistula (GRADE 1B, expert agreement 97.3%, audience agreement 96.7%).
Comments L35-R35: 4 RCTs have compared stapling devices with other types of stump closure, aiming to examine their impact on CR-POPF incidence after distal pancreatectomy. ^179-182^ All of these included both minimally invasive and open procedures suggesting no advantages for staplers. One retrospective, propensity-score matched study including both open and minimally invasive resections reported CR-POPF reduction using reinforced tri-staplers compared with ultrasonic dissection. ^183^ By contrast, another report found fewer CR-POPF for hand-sewn closures with retroperitoneal tissue covering than the stapling devices.^184^

**L36. Should staple line reinforcement versus no reinforcement be used for stump closure in LDP when a stapler is used?**Recommendation: Available evidence shows that the standard use of staple line reinforcements for pancreatic stump closure in LDP demonstrates no statistically clinical benefits over no reinforcement stapling (GRADE 1B, expert agreement 100.0%, audience agreement 82.1%).

**R36. Should staple line reinforcement versus no reinforcement be used for stump closure in RDP when a stapler is used?**Recommendation: Available evidence shows that the standard use of staple line reinforcements for pancreatic stump closure in RDP demonstrates no statistically clinical benefits over no reinforcement stapling (GRADE 1B, expert agreement 100.0%, audience agreement 82.1%).
Comments L36-R36: 5 RCTs, including both open and minimally-invasive procedures, have examined staple line reinforcement for CR-POPF prevention in distal pancreatectomy. One of these explored TachoSil, suggesting no significant effect. ^185^ The other 4 RCTs focused on reinforced staplers. Although one study observed a significant reduction in CR-POPF with reinforced staplers,^186^ this was not confirmed in the other RCTs. ^187-189^ The RCT on Hemopatch suggests no decrease in CR-POPF when analyzing the subgroup of patients with stapler closure. ^190^ One case-control study, including only laparoscopic procedures, reported no advantages for TachoSil,^191^ while another similar study observed a reduction in CR-POPF when the staple line was supplemented by additional sutures.^192^
Proposed action: Future RCTs and registry-based studies should further explore pathways for improving the stump closure technique and options for effective staple line reinforcement.

*Topic 16: Drain management Quality score: 56%* **L37. Are there any specific recommendations on the use and the positioning of drains in LDP, other than those known in the traditional open approach?**Recommendation: There is limited evidence to support the routine use of drains in LDP. Further studies are required (GRADE 1C, expert agreement 91.9%, audience agreement 77.8%).

**R37. Are there any specific recommendations on the use and the positioning of drains in RDP, other than those known in the traditional open approach?**Recommendation: There is limited evidence to support the routine use of drains in RDP. Further studies are required (GRADE 1C, expert agreement 91.9%, audience agreement 77.8%).
Comments L37-R37: 1 systematic review and 1 RCT concluded that omitting surgical drains after DP does not lead to higher complication rates or reinterventions.^193, 194^ Most papers included in the meta-analysis had mixed metrics but reported no significant difference between the drain and the no-drain group in terms of mortality and morbidity^1^. Based on this evidence, experts of the EGUMIPS meeting recommended that standard drain placement might not be advocated routinely in DP, in tertiary, referral, and high-volume centres with 24/7 available interventional radiology facilities, without specific recommendations for minimally invasive approaches over open surgery. During the meeting, this statement received an audience agreement of 54.4% and the validation committee found the literature limited to support this statement, so the statement was changed into the current statement. However, still no audience agreement above 80% could be reached.

**L38. Are there any specific recommendations on the use and the positioning of drains in LPD, other than those known in the traditional open approach?**Recommendation: Drain placement could be considered during LPD depending on patient, pancreas and procedure risks, regardless of the approach. However, no evidence exists on the specific use of drains in LPD (GRADE 1B, expert agreement 100.0%, audience agreement 85.1%).

**R38. Are there any specific recommendations on the use and the positioning of drains in RPD, other than those known in the traditional open approach?**Recommendation: Drain placement could be considered during RPD depending on patient, pancreas and procedure risks, regardless of the approach. However, no evidence exists on the specific use of drains in RPD (GRADE 1B, expert agreement 100.0%, audience agreement 85.1%).
Comments L38-R38: 11 studies^195-205^ investigated drain placement after PD, including 3 RCTs^199, 202, 204^ and 2 meta-analyses.^203, 205^ One RCT reported that drain elimination during PD was associated with increased morbidity and mortality.^202^ This trial was prematurely stopped but was the only one including laparoscopic procedures. Several subsequent studies highlighted the danger of abandoning routine intraperitoneal drainage during PD. Another RCT reported a high deviation rate from a standard no-drain protocol in higher-risk procedures.^204^ Once stratified by fistula risk, patients with negligible/low risk benefited from drain omission.^200^ A recent meta-analysis concluded that no difference occurred between drains and no drains groups, but it included stratified and no-stratified cohorts.^205^ Given no evidence in MIPD, the same indication for open procedures should be applied in MIPD until further evidence is reached.

**DOMAIN 6. ASSESSMENT TOOLS**
*Topic 17: Tools and Methods Quality score: 59%***L39. Which parameters should be defined as core outcomes in the assessment of LPR?**Recommendation: Core parameters in the assessment of LPR should include: severe morbidity, mortality, postoperative pancreatic fistula, conversion rate and patient-reported outcomes. Postoperative complications should be assessed up to 90 days. R0 resection rate, 3-year overall survival, and disease-free survival should be considered core outcomes for PDAC (GRADE 1 EXPERT OPINION, expert agreement 86.5%, audience agreement 95.0%).

**R39. Which parameters should be defined as core outcomes in the assessment of RPR?**Recommendation: Core parameters in the assessment of RPR should include: severe morbidity, mortality, postoperative pancreatic fistula, conversion rate and patient-reported outcomes. Postoperative complications should be assessed up to 90 days. R0 resection rate, 3-year overall survival, and disease-free survival should be considered core outcomes for PDAC (GRADE 1 EXPERT OPINION, expert agreement 83.8%, audience agreement 98.7%).
Comments L39-R39: When analyzing the RCTs available in the literature, there is wide variability in primary outcomes chosen in the different studies. The most frequently assessed postoperative outcomes are severe morbidity, mortality, postoperative pancreatic fistula, length of stay (LOS), time to functional recovery (TTFR), and conversion rate. However, LOS has been known to be affected by social, logistic, and cultural factors that can differ among departments and countries and should therefore not be considered a core outcome. TTFR is a more objective outcome but is dependent on patient compliance. In studies regarding laparoscopic pancreatic resections for PDAC R0 resection rate, 3-year overall survival and disease-free survival are the most used primary outcomes to assess MIPS.

**L40.** **Which are the most suitable outcome measurements that can be used to assess the validity of LPR?**Recommendation L40.1: The most suitable outcome measurements to assess the validity and efficacy of LPR are procedural outcomes as Benchmarks, Textbook Outcome, Comprehensive Complication Index, and Clavien-Dindo classification and patient outcomes as Patient Reported Outcome Measures (PROMs) and Quality-Adjusted Life Year (QALY). However, system-based outcomes such as costs, time, and resources should also be taken into consideration. All outcome measurements should be applied as appropriate based on the validity and efficacy aspect being measured (GRADE 1 EXPERT OPINION, expert agreement 97.3%, audience agreement 98.9%).
Recommendation L40.2: A multidimensional composite outcome measure should be developed in order to assess the entire operative process and validity of LPR (GRADE 1 EXPERT OPINION, expert agreement 97.3%, audience agreement 95.2%).

**R40.** **Which are the most suitable outcome measurements that can be used to assess the validity of RPR?**Recommendation R40.1: The most suitable outcome measurements to assess the validity and efficacy of RPR are procedural outcomes as Benchmarks, Textbook Outcome, Comprehensive Complication Index, and Clavien-Dindo classification and patient outcomes as Patient Reported Outcome Measures (PROMs) and Quality-Adjusted Life Year (QALY). However, system-based outcomes such as costs, time, and resources should also be taken into consideration. All outcome measurements should be applied as appropriate based on the validity and efficacy aspect being measured (GRADE 1 EXPERT OPINION, expert agreement 97.3%, audience agreement 98.8%).
Recommendation R40.2: A multidimensional composite outcome measure should be developed in order to assess the entire operative process and validity of RPR (GRADE 1 EXPERT OPINION, expert agreement 100.0%, audience agreement 95.2%).
Comments L40-R40: A full assessment of the validity of MIPS depends on many aspects as the overall burden or success of a minimally invasive procedure is influenced by multiple clinical and patient-specific factors. Single outcome measures are therefore insufficient, and the assessment of the validity of MIPS requires a broader judgment. However, no outcome measurements exist that capture all aspects of the validity or clinical assessment of MIPS. Therefore, it is essential to consider which aspect of validity is being assessed and which outcome measure fits as appropriate.

**DOMAIN 7. IMPLEMENTATION AND TRAINING**
*Topic 18: Volumes and Learning Curves Quality score: 74%***L41. What center volume should be maintained for the safe implementation of LPR (LPD/LDP)?**Recommendation: Center volume strongly affects outcomes after LPD. Morbidity, mortality, and R0 rate are better when LPD is done in centers performing at least 20 LPD procedures per year. Centers should aim to perform at least 20 LPD procedures per year, however it may be acceptable for centers to perform a lower volume per year as long as they can demonstrate maintenance of equivalent outcomes and they have a well-trained multidisciplinary pancreas team (GRADE 1B, expert agreement 91.9%, audience agreement 86.5%).

**R41. What center volume should be maintained for the safe implementation of RPR (RPD/RDP)?**Recommendation: Center volume strongly affects outcomes after RPD. Morbidity, mortality, and R0 rate are better when RPD is done in centers performing at least 20 LPD procedures per year. Centers should aim to perform at least 20 RPD procedures per year, however it may be acceptable for centers to perform a lower volume per year as long as they can demonstrate maintenance of equivalent outcomes and they have a well-trained multidisciplinary pancreas team (GRADE 1B, expert agreement 91.9%, audience agreement 86.5%).
Comments L41-R41: Studies assessing the effect of center volume on outcomes in LPD or RPD, reported a significant association between increasing hospital volume and improved outcomes in MIPD.^206-210^ A threshold of at least 20 MIPD cases/year seems to result in significantly lower postoperative morbidity and mortality. No specific data on center volume of LDP or RDP are available.
Proposed action L41-R41: Centers performing MIPS should be recommended to participate in national and regional (European) registries. Consequently, more data on center volume and outcomes will become accessible.

**L42. What are the suggested learning curves and surgeon volumes for LPR (LPD/LDP)?**Recommendation: The learning curve for operative time is 16 procedures for LDP and 39 for LPD. The learning curve for postoperative complications is 25 procedures for LDP and 25-80 for LPD. During the learning curve surgeons are recommended to participate in a structured training program and ensure that competency is reached (GRADE 1B, expert agreement 86.5%, audience agreement 92.0%).
Comments: In recent years, learning curves of LDP^211-215^ and LPD^82, 211, 214, 216-220^ have increasingly been studied. The majority of the studies in LDP identified learning curves of at least 15 procedures to reach the first phase and 25 procedures to reach the second phase. In LPD, more procedures are needed to surpass the learning curve of which the majority of the studies described around 35-40 procedures for the first phase, but staying inconclusive on the second phase reporting a wide range of 25-80 procedures. No conclusive data on surgeon volume are available.

**R42. What are the suggested learning curves and surgeon volumes for RPR (RPD/RDP)?**Recommendation: The learning curve for operative time is 15 procedures for RDP and 25 for RPD. The learning curve for postoperative complications is 21 for RDP and 25-40 for RPD. During this period, surgeons are recommended to participate in a structured training program and assure that competency is reached (GRADE 1B, expert agreement 91.9%, audience agreement 88.5%).
Comments: Available literature report learning curves in RDP ranging from 15 to 21 procedures.^211, 214, 221, 222^ In RPD, the majority of the studies identified at least 25 procedures as first phase and for the second phase a range of 25-40 procedures is observed.^161, 211, 214, 218, 223-226^. The learning curves of RPD are suggested to be lower as compared to LPD due to trainings programs and previous experience in LPD. No conclusive data on surgeon volume are available.
Proposed action L42-R42: To minimize potential bias, studies reporting on learning curves of pancreatic surgery should include information on institutional factors, with at least data on frequency of the procedure and team familiarity. A difficulty scoring system taking into account type of operation, risk factors, resectability and anatomic variations, would enhance the comparability of learning curves between studies and is therefore recommended. There is currently no gold standard in the evaluation of a learning curve. More work needs to be done to standardize learning curve assessment in a patient-centered manner. Formal mentorship and proficiency-based curricula should become a validated way to decrease learning curves while maintaining quality patient outcomes.

*Topic 19: Training Quality score: 68%*
**L43. What training and preparation should surgeons pursue before performing LPR and what is their impact?**Recommendation: A potentially higher rate of severe complications suggests the need for caution in introducing LPR techniques. Procedure-specific training programs for LPR mitigated the learning curve. Formal mentorship and structured training programs, which could include virtual reality, bio tissue drills, and off- and on-site proctoring facilitate safe introduction and expansion of LPR (GRADE 2B, expert agreement 100.0%, audience agreement 93.0%).

**R43. What training and preparation should surgeons pursue before performing RPR and what is their impact?**Recommendation: A potentially higher rate of severe complications suggests the need for caution in introducing RPR techniques. Procedure-specific training programs for RPR mitigated the learning curve. Formal mentorship and structured training programs, which could include virtual reality, bio tissue drills, and off- and on-site proctoring facilitate safe introduction and expansion of RPR (GRADE 2B, expert agreement 100.0%, audience agreement 96.5%).
Comments L43-R43: The main body of work in this topic comes from limited number of institutions with a high volume and increased experience over time; therefore, there is a concern regarding generalizability of their findings to other institutions. Most studies are retrospective observational single-center studies and difficult to replicate at another institution and RCTs are lacking.^227-238^
Proposed action L43-R43: There are currently two reports on the feasibility and impact of national training programs for MIPR from the Dutch group.^239, 240^ Future studies should determine whether such a training program is applicable in other settings.

*Topic 20: Registries Quality score: 60%* **G44. What should be the role of national and international registries in the wider implementation of MIPS?**Recommendation: The wider implementation of MIPS should be promoted by national and international HPB associations who should strongly encourage the development, implementation and coordination of national registries and participation into international registries, as it will enhance the position of the country in the international debate and propagate/disseminate collaborative studies, e.g., snapshot studies (GRADE 1B, expert agreement 97.3%, audience agreement 96.6%).

**G45. Should centers be asked to include patients having MIPS in registries for quality control?**Recommendation: For MIPS, inclusion into registries for quality control by validated national and international centralized registries should be strongly encouraged to allow for transparent analysis and discussions for surgical procedures over time and new surgical techniques (GRADE 1B, expert agreement 97.3%, audience agreement 97.9%).
Comments G44-G45: Several national as well as European registries currently exist to monitor the use and outcome of MIPS, who are positively received.^210, 241-244^ Participation in these registries or the development of new registries should be continued to be encouraged.
Proposed action G44-G45**:** Data on long-term survival should be aimed to be collected as well in registries.

*Topic 21: Cost-effectiveness Quality score: 58%***L46. Is the laparoscopic approach more costly than the traditional open approach?**Recommendation: The intraoperative costs are higher for LPR compared to OPR but may be offset by the reduction in length of hospital stay and functional recovery time (GRADE 1B, expert agreement 89.2%, audience agreement 82.8%).
Comments: Multiple studies have reported on costs in LPR compared to OPR. Most of the studies reported higher intraoperative costs in LPR but at least comparable or higher cost-effectiveness as compared to OPR.^245-252^ This is due to the reduction in length of hospital stay, functional recovery time and quality-adjusted life-years in LPR.

**R46. Is the robot-assisted approach more costly than the traditional open approach?**Recommendation: Studies assessing costs for robot-assisted pancreatic surgery are encouraged and should include capital costs, maintenance and training (GRADE 1C, expert agreement 100.0%, audience agreement 94.1%).
Comments: Limited and contradicting evidence is available to provide conclusions on the costs of RPR as compared to OPR. RPR seem to be associated with higher intraoperative costs, but also with reduced length of stays and lower median payments during the index hospitalization, resulting in lower total costs as compared to OPR or LPR.^246, 253, 254^
Proposed action L46-R46: Future studies should aim to apply similar definitions of intraoperative and inter-hospital costs. Also, more data on inter-country variabilities on healthcare financing should be collected to provide stronger future conclusions.  **DOMAIN 8. ARTIFICIAL INTELLIGENCE (AI)**
*Topic 22: The role of AI in pancreatic surgery Quality score: 51%* **L47. What is the role of AI in the future expansion of LPR?**Recommendation L47.1: AI in MIPS is expected to impact all areas of surgical practice, from preoperative risk assessment and surgical planning to augmenting surgeons' intra-operative abilities up to tailored follow-up strategies. Surgeons should be continuously updating themselves with these upcoming innovations and encourage their introduction into their clinical practice once their validity is proven. Surgeons should be encouraged to facilitate the development of AI data gathering (GRADE 2C, expert agreement 97.3%, audience agreement 92.4%).
Recommendation L47.2: Enhanced computer vision with augmented reality, virtual reality, and mixed reality are fields of AI that should continue to be developed because these technologies have great potential to improve surgical outcomes and to reduce short-term complications of LPR (GRADE 2C, expert agreement 97.3%, audience agreement 91.0%).
Comments: Currently, no evidence exists regarding the role of AI in laparoscopic or robotic pancreatic surgery. Published studies have mainly focused on assessing the technical feasibility of utilizing AI, and there are no studies with proven clinical implementation and validation at multiple centers. Investigated AI interventions encompass preoperative risk assessment, augmenting the surgeon's intraoperative skills or tasking surgical automation, and designing specific follow-up strategies.
Proposed action: Future research on delivering and assessing the clinical impact of these promising techniques is warranted to achieve individualized treatments and improve surgical outcomes.

**R47. What is the role of AI in the future expansion of RPR?**Recommendation R47.1: Autonomous actions are central to AI in future RPR. Today, tools with only a level 1 (telemanipulation – robot-assisted surgery) or a level 2 (limited autonomous actions) and arguably level 3 autonomy are available. Level 4 and 5 autonomy could eventually be possible; however, as of now, surgery should not be done without the control of a human surgeon who is fully capable of performing and completing the procedure via robotic assistance, standard laparoscopy and/or open surgical techniques. Surgeons should be encouraged to facilitate the development of AI data gathering (GRADE 2C, expert agreement 100.0%, audience agreement 87.8%).
Recommendation R47.2: Initially, it may be preferable for only parts of pancreatic surgical procedures to become fully automated with an emphasis on creating autonomous dexemes (parts of surgical gestures) and then surgemes (parts of entire operations) that are safe for patients and can be proven to provide patient benefit (GRADE 2C, expert agreement 97.3%, audience agreement 82.9%).
Comments: With robotics implementation in operation theatres, AI in surgery involve autonomous movements. Complete surgical systems are examples of only telemanipulation (level 1 autonomy) today. By contrast, many surgical devices already used in clinical practice may be considered with a higher level of autonomy, as they modify their action based on incoming data adapting independently from human control (level 2 autonomy).
Proposed action: Participation in retrospective and/or prospectively maintained database studies and registries is recommended, and the development of AI with strict human supervision for the expansion of MIPS should be encouraged.

**REFERENCES**

1. Montagnini AL, Rosok BI, Asbun HJ, et al. Standardizing terminology for minimally invasive pancreatic resection. *HPB (Oxford)* 2017; 19(3):182-189.

2. Lof S, Korrel M, van Hilst J, et al. Outcomes of Elective and Emergency Conversion in Minimally Invasive Distal Pancreatectomy for Pancreatic Ductal Adenocarcinoma: An International Multicenter Propensity Score-matched Study. *Ann Surg* 2021; 274(6):e1001-e1007.

3. Lof S, Vissers FL, Klompmaker S, et al. Risk of conversion to open surgery during robotic and laparoscopic pancreatoduodenectomy and effect on outcomes: international propensity score-matched comparison study. *Br J Surg* 2021; 108(1):80-87.

4. Al-Sadairi AR, Mimmo A, Rhaiem R, et al. Laparoscopic hybrid pancreaticoduodenectomy: Initial single center experience. *Ann Hepatobiliary Pancreat Surg* 2021; 25(1):102-111.

5. Deichmann S, Bolm LR, Honselmann KC, et al. Perioperative and Long-term Oncological Results of Minimally Invasive Pancreatoduodenectomy as Hybrid Technique - A Matched Pair Analysis of 120 Cases. *Zentralbl Chir* 2018; 143(2):155-161.

6. Goh BKP, Low TY, Kam JH, et al. Initial experience with laparoscopic and robotic surgery for the treatment of periampullary tumours: single institution experience with the first 30 consecutive cases. *ANZ J Surg* 2019; 89(4):E137-E141.

7. Klompmaker S, van Hilst J, Wellner UF, et al. Outcomes After Minimally-invasive Versus Open Pancreatoduodenectomy: A Pan-European Propensity Score Matched Study. *Ann Surg* 2020; 271(2):356-363.

8. Nieuwenhuijs VB, de Klein GW, van Duijvendijk P, et al. Lessons Learned from the Introduction of Laparoscopic Pancreaticoduodenectomy. *J Laparoendosc Adv Surg Tech A* 2020; 30(5):495-500.

9. Patel B, Leung U, Lee J, et al. Laparoscopic pancreaticoduodenectomy in Brisbane, Australia: an initial experience. *ANZ J Surg* 2018; 88(5):E440-E444.

10. Pham H, Nahm CB, Hollands M, et al. Hybrid laparoscopic pancreaticoduodenectomy: an Australian experience and a proposed process for implementation. *ANZ J Surg* 2020; 90(7-8):1422-1427.

11. Piedimonte S, Wang Y, Bergman S, et al. Early experience with robotic pancreatic surgery in a Canadian institution. *Can J Surg* 2015; 58(6):394-401.

12. Speicher PJ, Nussbaum DP, White RR, et al. Defining the learning curve for team-based laparoscopic pancreaticoduodenectomy. *Ann Surg Oncol* 2014; 21(12):4014-9.

13. Tyutyunnik P, Klompmaker S, Lombardo C, et al. Learning curve of three European centers in laparoscopic, hybrid laparoscopic, and robotic pancreatoduodenectomy. *Surg Endosc* 2022; 36(2):1515-1526.

14. Vladimirov M, Bausch D, Stein HJ, et al. Hybrid Laparoscopic Versus Open Pancreatoduodenectomy. A Meta-Analysis. *World J Surg* 2022; 46(4):901-915.

15. Wang C, Qi R, Li H, et al. Comparison of Perioperative and Oncological Outcomes of Hybrid and Totally Laparoscopic Pancreatoduodenectomy. *Med Sci Monit* 2020; 26:e924190.

16. Wellner UF, Kusters S, Sick O, et al. Hybrid laparoscopic versus open pylorus-preserving pancreatoduodenectomy: retrospective matched case comparison in 80 patients. *Langenbecks Arch Surg* 2014; 399(7):849-56.

17. de Rooij T, van Hilst J, van Santvoort H, et al. Minimally Invasive Versus Open Distal Pancreatectomy (LEOPARD): A Multicenter Patient-blinded Randomized Controlled Trial. *Ann Surg* 2019; 269(1):2-9.

18. Bjornsson B, Larsson AL, Hjalmarsson C, et al. Comparison of the duration of hospital stay after laparoscopic or open distal pancreatectomy: randomized controlled trial. *Br J Surg* 2020; 107(10):1281-1288.

19. Asbun HJ, Moekotte AL, Vissers FL, et al. The Miami International Evidence-based Guidelines on Minimally Invasive Pancreas Resection. *Ann Surg* 2020; 271(1):1-14.

20. Alfieri S, Butturini G, Boggi U, et al. Short-term and long-term outcomes after robot-assisted versus laparoscopic distal pancreatectomy for pancreatic neuroendocrine tumors (pNETs): a multicenter comparative study. *Langenbeck's Archives of Surgery* 2019; 404:459-468.

21. Chen S, Zhan Q, Chen JZ, et al. Robotic approach improves spleen-preserving rate and shortens postoperative hospital stay of laparoscopic distal pancreatectomy: a matched cohort study. *Surgical Endoscopy* 2015; 29:3507-3518.

22. Eckhardt S, Schicker C, Maurer E, et al. Robotic-Assisted Approach Improves Vessel Preservation in Spleen-Preserving Distal Pancreatectomy. *Digestive surgery* 2016; 33:406-413.

23. Jiang Y, Zheng K, Zhang S, et al. Robot-assisted distal pancreatectomy improves spleen preservation rate versus laparoscopic distal pancreatectomy for benign and low-grade malignant lesions of the pancreas. *Translational Cancer Research* 2020; 9:5166-5172.

24. Najafi N, Mintziras I, Wiese D, et al. A retrospective comparison of robotic versus laparoscopic distal resection and enucleation for potentially benign pancreatic neoplasms. *Surgery Today* 2020; 50:872-880.

25. Rompianesi G, Montalti R, Ambrosio L, et al. Robotic versus Laparoscopic Surgery for Spleen-Preserving Distal Pancreatectomies: Systematic Review and Meta-Analysis. *J Pers Med* 2021; 11(6).

26. Yang SJ, Hwang HK, Kang CM, et al. Revisiting the potential advantage of robotic surgical system in spleen-preserving distal pancreatectomy over conventional laparoscopic approach. *Annals of Translational Medicine* 2020; 8:188.

27. Zhang J, Jin J, Chen S, et al. Minimally invasive distal pancreatectomy for PNETs: laparoscopic or robotic approach? *Oncotarget* 2017; 8(20):33872-33883.

28. Kim H, Song KB, Hwang DW, et al. Laparoscopic versus open pancreaticoduodenectomy for pancreatic neuroendocrine tumors: a single-center experience. *Surg Endosc* 2019; 33(12):4177-4185.

29. Zheng J, Pulvirenti A, Javed AA, et al. Minimally Invasive vs Open Pancreatectomy for Pancreatic Neuroendocrine Tumors: Multi-Institutional 10-Year Experience of 1,023 Patients. *J Am Coll Surg* 2022; 235(2):315-330.

30. Takagi K, Umeda Y, Yoshida R, et al. A Systematic Review of Minimally Invasive Versus Open Radical Antegrade Modular Pancreatosplenectomy for Pancreatic Cancer. *Anticancer Res* 2022; 42(2):653-660.

31. van Hilst J, Korrel M, de Rooij T, et al. Oncologic outcomes of minimally invasive versus open distal pancreatectomy for pancreatic ductal adenocarcinoma: A systematic review and meta-analysis. *Eur J Surg Oncol* 2019; 45(5):719-727.

32. Raoof M, Nota C, Melstrom LG, et al. Oncologic outcomes after robot-assisted versus laparoscopic distal pancreatectomy: Analysis of the National Cancer Database. *J Surg Oncol* 2018; 118(4):651-656.

33. Qu L, Zhiming Z, Xianglong T, et al. Short- and mid-term outcomes of robotic versus laparoscopic distal pancreatosplenectomy for pancreatic ductal adenocarcinoma: A retrospective propensity score-matched study. *International Journal of Surgery* 2018; 55:81-86.

34. Baimas-George M, Watson M, Salibi P, et al. Oncologic Outcomes of Robotic Left Pancreatectomy for Pancreatic Adenocarcinoma: a Single-Center Comparison to Laparoscopic Resection. *American surgeon* 2020:3134820949524.

35. Feng Q, Jiang C, Feng X, et al. Robotic Versus Laparoscopic Distal Pancreatectomy for Pancreatic Ductal Adenocarcinoma: A Systematic Review and Meta-Analysis. *Front Oncol* 2021; 11:752236.

36. Chopra A, Nassour I, Zureikat A, et al. Perioperative and oncologic outcomes of open, laparoscopic, and robotic distal pancreatectomy for pancreatic adenocarcinoma. *Updates in surgery* 2021; 73:947-953.

37. Ocuin LM, Miller-Ocuin JL, Novak SM, et al. Robotic and open distal pancreatectomy with celiac axis resection for locally advanced pancreatic body tumors: a single institutional assessment of perioperative outcomes and survival. *HPB (Oxford)* 2016; 18(10):835-842.

38. Nassour I, Winters SB, Hoehn R, et al. Long-term oncologic outcomes of robotic and open pancreatectomy in a national cohort of pancreatic adenocarcinoma. *J Surg Oncol* 2020; 122(2):234-242.

39. Kantor O, Talamonti MS, Sharpe S, et al. Laparoscopic pancreaticoduodenectomy for adenocarcinoma provides short-term oncologic outcomes and long-term overall survival rates similar to those for open pancreaticoduodenectomy. *Am J Surg* 2017; 213(3):512-515.

40. Chapman BC, Gajdos C, Hosokawa P, et al. Comparison of laparoscopic to open pancreaticoduodenectomy in elderly patients with pancreatic adenocarcinoma. *Surg Endosc* 2018; 32(5):2239-2248.

41. Stauffer JA, Coppola A, Villacreses D, et al. Laparoscopic versus open pancreaticoduodenectomy for pancreatic adenocarcinoma: long-term results at a single institution. *Surg Endosc* 2017; 31(5):2233-2241.

42. Zhou W, Jin W, Wang D, et al. Laparoscopic versus open pancreaticoduodenectomy for pancreatic ductal adenocarcinoma: a propensity score matching analysis. *Cancer Commun (Lond)* 2019; 39(1):66.

43. Kwon J, Song KB, Park SY, et al. Comparison of Minimally Invasive Versus Open Pancreatoduodenectomy for Pancreatic Ductal Adenocarcinoma: A Propensity Score Matching Analysis. *Cancers (Basel)* 2020; 12(4).

44. Yin Z, Jian Z, Hou B, et al. Surgical and Oncological Outcomes of Laparoscopic Versus Open Pancreaticoduodenectomy in Patients With Pancreatic Duct Adenocarcinoma. *Pancreas* 2019; 48(7):861-867.

45. Jiang YL, Zhang RC, Zhou YC. Comparison of overall survival and perioperative outcomes of laparoscopic pancreaticoduodenectomy and open pancreaticoduodenectomy for pancreatic ductal adenocarcinoma: a systematic review and meta-analysis. *BMC Cancer* 2019; 19(1):781.

46. Chen K, Zhou Y, Jin W, et al. Laparoscopic pancreaticoduodenectomy versus open pancreaticoduodenectomy for pancreatic ductal adenocarcinoma: oncologic outcomes and long-term survival. *Surg Endosc* 2020; 34(5):1948-1958.

47. Feng Q, Liao W, Xin Z, et al. Laparoscopic Pancreaticoduodenectomy Versus Conventional Open Approach for Patients With Pancreatic Duct Adenocarcinoma: An Up-to-Date Systematic Review and Meta-Analysis. *Front Oncol* 2021; 11:749140.

48. Weng Y, Jiang Y, Fu N, et al. Oncological outcomes of robotic-assisted versus open pancreatoduodenectomy for pancreatic ductal adenocarcinoma: a propensity score-matched analysis. *Surg Endosc* 2021; 35(7):3437-3448.

49. Girgis MD, Zenati MS, King JC, et al. Oncologic Outcomes After Robotic Pancreatic Resections Are Not Inferior to Open Surgery. *Ann Surg* 2021; 274(3):e262-e268.

50. Chen K, Liu XL, Pan Y, et al. Expanding laparoscopic pancreaticoduodenectomy to pancreatic-head and periampullary malignancy: major findings based on systematic review and meta-analysis. *BMC Gastroenterol* 2018; 18(1):102.

51. Hakeem AR, Verbeke CS, Cairns A, et al. A matched-pair analysis of laparoscopic versus open pancreaticoduodenectomy: oncological outcomes using Leeds Pathology Protocol. *Hepatobiliary & Pancreatic Diseases International* 2014; 13(4):435-441.

52. Meng L-W, Cai Y-Q, Li Y-B, et al. Comparison of Laparoscopic and Open Pancreaticoduodenectomy for the Treatment of Nonpancreatic Periampullary Adenocarcinomas. *Surg Laparosc Endosc Percutan Tech* 2018; 28.

53. Dang C, Wang M, Zhu F, et al. Comparison of laparoscopic and open pancreaticoduodenectomy for the treatment of nonpancreatic periampullary adenocarcinomas: a propensity score matching analysis. *Am J Surg* 2021; 222(2):377-382.

54. Shin H, Song KB, Kim YI, et al. Propensity score-matching analysis comparing laparoscopic and open pancreaticoduodenectomy in elderly patients. *Sci Rep* 2019; 9(1):12961.

55. van der Heijde N, Balduzzi A, Alseidi A, et al. The role of older age and obesity in minimally invasive and open pancreatic surgery: A systematic review and meta-analysis. *Pancreatology* 2020; 20(6):1234-1242.

56. Zhang W, Huang Z, Zhang J, et al. Effect of Laparoscopic Pancreaticoduodenectomy in Elderly People: A Meta-analysis. *Pancreas* 2021; 50(8):1154-1162.

57. Nassour I, Wang SC, Porembka MR, et al. Robotic Versus Laparoscopic Pancreaticoduodenectomy: a NSQIP Analysis. *J Gastrointest Surg* 2017; 21(11):1784-1792.

58. Yan-Shen CY-JT-KP-JC-J. Impact of body mass index on the early experience of robotic pancreaticoduodenectomy. *Updates in surgery* 2021; 73(3):929-937.

59. Cho CS, Kooby DA, Schmidt CM, et al. Laparoscopic versus open left pancreatectomy: can preoperative factors indicate the safer technique? *Ann Surg* 2011; 253(5):975-80.

60. Sahakyan MA, Tholfsen T, Kleive D, et al. Laparoscopic Distal Pancreatectomy Following Prior Upper Abdominal Surgery (Pancreatectomy and Prior Surgery). *J Gastrointest Surg* 2021; 25(7):1787-1794.

61. Liu Q, Zhao Z, Zhang X, et al. Robotic pancreaticoduodenectomy in elderly and younger patients: A retrospective cohort study. *Int J Surg* 2020; 81:61-65.

62. Paolini C, Bencini L, Gabellini L, et al. Robotic versus open pancreaticoduodenectomy: Is there any difference for frail patients? *Surg Oncol* 2021; 37:101515.

63. Zhu J, Wang G, Du P, et al. Minimally Invasive Pancreaticoduodenectomy in Elderly Patients: Systematic Review and Meta-Analysis. *World J Surg* 2021; 45(4):1186-1201.

64. Girgis MD, Zenati MS, Steve J, et al. Robotic approach mitigates perioperative morbidity in obese patients following pancreaticoduodenectomy. *HPB (Oxford)* 2017; 19(2):93-98.

65. He S, Ding D, Wright MJ, et al. The impact of high body mass index on patients undergoing robotic pancreatectomy: A propensity matched analysis. *Surgery* 2020; 167(3):556-559.

66. Wang SE, Daskalaki D, Masrur MA, et al. Impact of Obesity on Robot-Assisted Distal Pancreatectomy. *J Laparoendosc Adv Surg Tech A* 2016; 26(7):551-6.

67. Ritschl PV, Miller HK, Hillebrandt K, et al. Feasibility of robotic-assisted pancreatic resection in patients with previous minor abdominal surgeries: a single-center experience of the first three years. *BMC Surg* 2022; 22(1):86.

68. Liu R, Liu Q, Zhao ZM, et al. Robotic versus laparoscopic distal pancreatectomy: A propensity score-matched study. *Journal of Surgical Oncology* 2017; 116:461-469.

69. Cai Y, Gao P, Li Y, et al. Laparoscopic pancreaticoduodenectomy with major venous resection and reconstruction: anterior superior mesenteric artery first approach. *Surg Endosc* 2018; 32(10):4209-4215.

70. Croome KP, Farnell MB, Que FG, et al. Pancreaticoduodenectomy with major vascular resection: a comparison of laparoscopic versus open approaches. *J Gastrointest Surg* 2015; 19(1):189-94; discussion 194.

71. Geers J, Topal H, Jaekers J, et al. 3D-laparoscopic pancreaticoduodenectomy with superior mesenteric or portal vein resection for pancreatic cancer. *Surg Endosc* 2020; 34(12):5616-5624.

72. Du Y, Wang J, Li Y, et al. Clinical application of a modified pancreatojejunostomy technique for laparoscopic pancreaticoduodenectomy. *HPB (Oxford)* 2019; 21(10):1336-1343.

73. Kwon J, Shin SH, Lee S, et al. The Effect of Fibrinogen/Thrombin-Coated Collagen Patch (TachoSil(®)) Application in Pancreaticojejunostomy for Prevention of Pancreatic Fistula After Pancreaticoduodenectomy: A Randomized Clinical Trial. *World J Surg* 2019; 43(12):3128-3137.

74. Poves I, Morató O, Burdío F, et al. Laparoscopic-adapted Blumgart pancreaticojejunostomy in laparoscopic pancreaticoduodenectomy. *Surg Endosc* 2017; 31(7):2837-2845.

75. Wang W, Liu Q, Zhao Z, et al. Robotic versus open total pancreatectomy: a retrospective cohort study. *Langenbecks Arch Surg* 2021; 406(7):2325-2332.

76. Li BQ, Qiao YX, Li J, et al. Preservation or Ligation of Splenic Vessels During Spleen-Preserving Distal Pancreatectomy: A Meta-Analysis. *J Invest Surg* 2019; 32(7):654-669.

77. Chen S, Zhan Q, Chen JZ, et al. Robotic approach improves spleen-preserving rate and shortens postoperative hospital stay of laparoscopic distal pancreatectomy: a matched cohort study. *Surg Endosc* 2015; 29(12):3507-18.

78. Yang SJ, Hwang HK, Kang CM, et al. Revisiting the potential advantage of robotic surgical system in spleen-preserving distal pancreatectomy over conventional laparoscopic approach. *Ann Transl Med* 2020; 8(5):188.

79. Huynh F, Cruz CJ, Hwang HK, et al. Minimally invasive (laparoscopic and robot-assisted) versus open approach for central pancreatectomies: a single-center experience. *Surg Endosc* 2022; 36(2):1326-1331.

80. Song KB, Kim SC, Park KM, et al. Laparoscopic central pancreatectomy for benign or low-grade malignant lesions in the pancreatic neck and proximal body. *Surg Endosc* 2015; 29(4):937-46.

81. Zhang RC, Zhang B, Mou YP, et al. Comparison of clinical outcomes and quality of life between laparoscopic and open central pancreatectomy with pancreaticojejunostomy. *Surg Endosc* 2017; 31(11):4756-4763.

82. Dokmak S, Ftériche FS, Aussilhou B, et al. The Largest European Single-Center Experience: 300 Laparoscopic Pancreatic Resections. *J Am Coll Surg* 2017; 225(2):226-234.e2.

83. Chen S, Zhan Q, Jin JB, et al. Robot-assisted laparoscopic versus open middle pancreatectomy: short-term results of a randomized controlled trial. *Surg Endosc* 2017; 31(2):962-971.

84. Rompianesi G, Montalti R, Giglio MC, et al. Robotic central pancreatectomy: a systematic review and meta-analysis. *HPB (Oxford)* 2022; 24(2):143-151.

85. Guerra F, Giuliani G, Bencini L, et al. Minimally invasive versus open pancreatic enucleation. Systematic review and meta-analysis of surgical outcomes. *J Surg Oncol* 2018; 117(7):1509-1516.

86. Zhou Y, Zhao M, Wu L, et al. Short- and long-term outcomes after enucleation of pancreatic tumors: An evidence-based assessment. *Pancreatology* 2016; 16(6):1092-1098.

87. Dalla Valle R, Cremaschi E, Lamecchi L, et al. Open and minimally invasive pancreatic neoplasms enucleation: a systematic review. *Surg Endosc* 2019; 33(10):3192-3199.

88. Ei S, Mihaljevic AL, Kulu Y, et al. Enucleation for benign or borderline tumors of the pancreas: comparing open and minimally invasive surgery. *HPB (Oxford)* 2021; 23(6):921-926.

89. Tian F, Hong XF, Wu WM, et al. Propensity score-matched analysis of robotic versus open surgical enucleation for small pancreatic neuroendocrine tumours. *Br J Surg* 2016; 103(10):1358-64.

90. Zakaria HM, Stauffer JA, Raimondo M, et al. Total pancreatectomy: Short- and long-term outcomes at a high-volume pancreas center. *World J Gastrointest Surg* 2016; 8(9):634-642.

91. Fan CJ, Hirose K, Walsh CM, et al. Laparoscopic Total Pancreatectomy With Islet Autotransplantation and Intraoperative Islet Separation as a Treatment for Patients With Chronic Pancreatitis. *JAMA Surg* 2017; 152(6):550-556.

92. Cai Y, Gao P, Peng B. A novel surgical approach for en-bloc resection laparoscopic total pancreatectomy. *Medicine (Baltimore)* 2020; 99(28):e20948.

93. John GK, Singh VK, Pasricha PJ, et al. Delayed Gastric Emptying (DGE) Following Total Pancreatectomy with Islet Auto Transplantation in Patients with Chronic Pancreatitis. *J Gastrointest Surg* 2015; 19(7):1256-61.

94. Konstantinidis IT, Jutric Z, Eng OS, et al. Robotic total pancreatectomy with splenectomy: technique and outcomes. *Surg Endosc* 2018; 32(8):3691-3696.

95. Zureikat AH, Nguyen T, Boone BA, et al. Robotic total pancreatectomy with or without autologous islet cell transplantation: replication of an open technique through a minimal access approach. *Surg Endosc* 2015; 29(1):176-83.

96. de Mesquita Neto JWB, Macedo FI, Liu Y, et al. Fully robotic total pancreatectomy: technical aspects and outcomes. *J Robot Surg* 2019; 13(1):77-82.

97. Weng Y, Chen M, Gemenetzis G, et al. Robotic-assisted versus open total pancreatectomy: a propensity score-matched study. *Hepatobiliary Surg Nutr* 2020; 9(6):759-770.

98. Kauffmann EF, Napoli N, Genovese V, et al. Feasibility and safety of robotic-assisted total pancreatectomy: a pilot western series. *Updates Surg* 2021; 73(3):955-966.

99. Boggi U, Palladino S, Massimetti G, et al. Laparoscopic robot-assisted versus open total pancreatectomy: a case-matched study. *Surg Endosc* 2015; 29(6):1425-32.

100. Morales E, Zimmitti G, Codignola C, et al. Follow "the superior mesenteric artery": laparoscopic approach for total mesopancreas excision during pancreaticoduodenectomy. *Surg Endosc* 2019; 33(12):4186-4191.

101. Honda G, Kurata M, Okuda Y, et al. Laparoscopic pancreaticoduodenectomy: taking advantage of the unique view from the caudal side. *J Am Coll Surg* 2013; 217(6):e45-9.

102. Jiang CY, Liang Y, Wang HW, et al. Management of the uncinate process via the artery first approach in laparoscopic pancreatoduodenectomy. *J Hepatobiliary Pancreat Sci* 2019; 26(9):410-415.

103. Machado MA, Mattos BV, Lobo Filho MM, et al. Robotic Artery-First Approach During Pancreatoduodenectomy. *Ann Surg Oncol* 2021; 28(11):6257-6261.

104. Pittau G, Sànchez-Cabùs S, Laurenzi A, et al. Laparoscopic Pancreaticoduodenectomy: Right Posterior Superior Mesenteric Artery "First" Approach. *Ann Surg Oncol* 2015; 22 Suppl 3:S345-8.

105. Wang XM, Sun WD, Hu MH, et al. Inferoposterior duodenal approach for laparoscopic pancreaticoduodenectomy. *World J Gastroenterol* 2016; 22(6):2142-8.

106. Cho A, Yamamoto H, Kainuma O. Tips of laparoscopic pancreaticoduodenectomy: superior mesenteric artery first approach (with video). *J Hepatobiliary Pancreat Sci* 2014; 21(3):E19-21.

107. Liao CH, Liu YY, Wang SY, et al. The feasibility of laparoscopic pancreaticoduodenectomy-a stepwise procedure and learning curve. *Langenbecks Arch Surg* 2017; 402(5):853-861.

108. Asbun HJ, Harada E, Stauffer JA. Tips for laparoscopic pancreaticoduodenectomy. *J Hepatobiliary Pancreat Sci* 2016; 23(3):E5-9.

109. Boggi U, Signori S, De Lio N, et al. Feasibility of robotic pancreaticoduodenectomy. *Br J Surg* 2013; 100(7):917-25.

110. Chen XM, Sun DL, Zhang Y. Laparoscopic versus open pancreaticoduodenectomy combined with uncinated process approach: A comparative study evaluating perioperative outcomes (Retrospective cohort study). *Int J Surg* 2018; 51:170-173.

111. Kendrick ML, Cusati D. Total laparoscopic pancreaticoduodenectomy: feasibility and outcome in an early experience. *Arch Surg* 2010; 145(1):19-23.

112. Kim H, Kim JR, Han Y, et al. Early experience of laparoscopic and robotic hybrid pancreaticoduodenectomy. *Int J Med Robot* 2017; 13(3).

113. Lai EC, Yang GP, Tang CN. Robot-assisted laparoscopic pancreaticoduodenectomy versus open pancreaticoduodenectomy--a comparative study. *Int J Surg* 2012; 10(9):475-9.

114. Liu R, Zhang T, Zhao ZM, et al. The surgical outcomes of robot-assisted laparoscopic pancreaticoduodenectomy versus laparoscopic pancreaticoduodenectomy for periampullary neoplasms: a comparative study of a single center. *Surg Endosc* 2017; 31(6):2380-2386.

115. Mendoza AS, 3rd, Han HS, Yoon YS, et al. Laparoscopy-assisted pancreaticoduodenectomy as minimally invasive surgery for periampullary tumors: a comparison of short-term clinical outcomes of laparoscopy-assisted pancreaticoduodenectomy and open pancreaticoduodenectomy. *J Hepatobiliary Pancreat Sci* 2015; 22(12):819-24.

116. Nagakawa Y, Hosokawa Y, Sahara Y, et al. Approaching the superior mesenteric artery from the right side using the proximal-dorsal jejunal vein preisolation method during laparoscopic pancreaticoduodenectomy. *Surg Endosc* 2018; 32(9):4044-4051.

117. Nagakawa Y, Hosokawa Y, Sahara Y, et al. A Novel "Artery First" Approach Allowing Safe Resection in Laparoscopic Pancreaticoduodenectomy: The Uncinate Process First Approach. *Hepatogastroenterology* 2015; 62(140):1037-40.

118. Palanivelu C, Rajan PS, Rangarajan M, et al. Evolution in techniques of laparoscopic pancreaticoduodenectomy: a decade long experience from a tertiary center. *J Hepatobiliary Pancreat Surg* 2009; 16(6):731-40.

119. Park MY, Lee W, Kwon J, et al. Comparison of perioperative outcomes in pancreatic head cancer patients following either a laparoscopic or open pancreaticoduodenectomy with a superior mesenteric artery first approach. *Ann Hepatobiliary Pancreat Surg* 2021; 25(3):358-365.

120. Pędziwiatr M, Pisarska M, Małczak P, et al. Laparoscopic uncinate process first pancreatoduodenectomy-feasibility study of a modified 'artery first' approach to pancreatic head cancer. *Langenbecks Arch Surg* 2017; 402(6):917-923.

121. Zhang Y, Sun DL, Chen XM. The Uncinate Process First Approach in Laparoscopic Pancreaticoduodenectomy: A Single-institution Experience. *Surg Laparosc Endosc Percutan Tech* 2017; 27(6):e141-e144.

122. Galvez D, Sorber R, Javed AA, et al. Technical considerations for the fully robotic pancreaticoduodenectomy. *J Vis Surg* 2017; 3:81.

123. Giulianotti PC, Mangano A, Bustos RE, et al. Operative technique in robotic pancreaticoduodenectomy (RPD) at University of Illinois at Chicago (UIC): 17 steps standardized technique : Lessons learned since the first worldwide RPD performed in the year 2001. *Surg Endosc* 2018; 32(10):4329-4336.

124. Jacobs MJ, Kamyab A. Total laparoscopic pancreaticoduodenectomy. *Jsls* 2013; 17(2):188-93.

125. Kim S, Yoon YS, Han HS, et al. A blunt dissection technique using the LigaSure vessel-sealing device improves perioperative outcomes and postoperative splenic-vessel patency after laparoscopic spleen- and splenic-vessel-preserving distal pancreatectomy. *Surg Endosc* 2018; 32(5):2550-2558.

126. Nagakawa Y, Sahara Y, Hosokawa Y, et al. The Straightened Splenic Vessels Method Improves Surgical Outcomes of Laparoscopic Distal Pancreatectomy. *Dig Surg* 2017; 34(4):289-297.

127. Yamada S, Fujii T, Kawai M, et al. Splenic vein resection together with the pancreatic parenchyma versus separated resection after isolation of the parenchyma during distal pancreatectomy (COSMOS-DP trial): study protocol for a randomised controlled trial. *Trials* 2018; 19(1):369.

128. Zhou ZQ, Kim SC, Song KB, et al. Laparoscopic spleen-preserving distal pancreatectomy: comparative study of spleen preservation with splenic vessel resection and splenic vessel preservation. *World J Surg* 2014; 38(11):2973-9.

129. Nishino H, Zimmitti G, Ohtsuka T, et al. Precision vascular anatomy for minimally invasive distal pancreatectomy: A systematic review. *J Hepatobiliary Pancreat Sci* 2022; 29(1):136-150.

130. Wada Y, Aoki T, Murakami M, et al. Individualized procedures for splenic artery dissection during laparoscopic distal pancreatectomy. *BMC Surg* 2020; 20(1):32.

131. Abu Hilal M, Richardson JR, de Rooij T, et al. Laparoscopic radical 'no-touch' left pancreatosplenectomy for pancreatic ductal adenocarcinoma: technique and results. *Surg Endosc* 2016; 30(9):3830-8.

132. Azagra JS, Rosso E, Pascotto B, et al. Real robotic total mesopancreas excision (TMpE) assisted by hanging manoeuver (HM): Standardised technique. *Int J Med Robot* 2021; 17(4):e2259.

133. Gehrig T, Josef V, Billeter AT, et al. Dissection with LigaSure Impact™ versus conventional resection in pylorus-preserving partial pancreatoduodenectomy (DISSECT): a single-institution randomized controlled trial. *Langenbecks Arch Surg* 2020; 405(7):949-958.

134. Uzunoglu FG, Stehr A, Fink JA, et al. Ultrasonic dissection versus conventional dissection techniques in pancreatic surgery: a randomized multicentre study. *Ann Surg* 2012; 256(5):675-9; discussion 679-80.

135. Uzunoglu FG, Bockhorn M, Fink JA, et al. LigaSure™ vs. conventional dissection techniques in pancreatic surgery--a prospective randomised single-centre trial. *J Gastrointest Surg* 2013; 17(3):494-500.

136. Gehrig T, Müller-Stich BP, Kenngott H, et al. LigaSure versus conventional dissection technique in pancreatoduodenectomy: a pilot study. *Am J Surg* 2011; 201(2):166-70.

137. Satoi S, Yanagimoto H, Toyokawa H. Use of the new ultrasonically curved shear in pancreaticoduodenectomy for periampullary cancer. *J Hepatobiliary Pancreat Sci* 2011; 18(4):609-14.

138. Heverhagen AE, Dietzel K, Waldmann J, et al. Harmonic scalpel versus conventional dissection technique in pylorus-preserving partial duodenopancreatectomy. *Dig Surg* 2012; 29(5):420-5.

139. Eng OS, Goswami J, Moore D, et al. Safety and efficacy of LigaSure usage in pancreaticoduodenectomy. *HPB (Oxford)* 2013; 15(10):747-52.

140. Yui R, Satoi S, Toyokawa H, et al. Less morbidity after introduction of a new departmental policy for patients who undergo open distal pancreatectomy. *J Hepatobiliary Pancreat Sci* 2014; 21(1):72-7.

141. D'Angelica M, Are C, Jarnagin W, et al. Initial experience with hand-assisted laparoscopic distal pancreatectomy. *Surg Endosc* 2006; 20(1):142-8.

142. Misawa T, Shiba H, Usuba T, et al. Safe and quick distal pancreatectomy using a staggered six-row stapler. *Am J Surg* 2008; 195(1):115-8.

143. Laxa BU, Carbonell AM, 2nd, Cobb WS, et al. Laparoscopic and hand-assisted distal pancreatectomy. *Am Surg* 2008; 74(6):481-6; discussion 486-7.

144. Luo Y, Liu R, Hu MG, et al. Laparoscopic surgery for pancreatic insulinomas: a single-institution experience of 29 cases. *J Gastrointest Surg* 2009; 13(5):945-50.

145. Gumbs AA, Chouillard EK. Laparoscopic distal pancreatectomy and splenectomy for malignant tumors. *J Gastrointest Cancer* 2012; 43(1):83-6.

146. Dokmak S, Aussilhou B, Sauvanet A, et al. Hand-assisted laparoscopic total pancreatectomy: a report of two cases. *J Laparoendosc Adv Surg Tech A* 2013; 23(6):539-44.

147. Allan BJ, Novak SM, Hogg ME, et al. Robotic vascular resections during Whipple procedure. *J Vis Surg* 2018; 4:13.

148. Beane JD, Zenati M, Hamad A, et al. Robotic pancreatoduodenectomy with vascular resection: Outcomes and learning curve. *Surgery* 2019; 166(1):8-14.

149. Boggi U, Napoli N, Costa F, et al. Robotic-Assisted Pancreatic Resections. *World J Surg* 2016; 40(10):2497-506.

150. Dokmak S, Aussilhou B, Calmels M, et al. Laparoscopic pancreaticoduodenectomy with reconstruction of the mesentericoportal vein with the parietal peritoneum and the falciform ligament. *Surg Endosc* 2018; 32(7):3256-3261.

151. Giulianotti PC, Addeo P, Buchs NC, et al. Robotic extended pancreatectomy with vascular resection for locally advanced pancreatic tumors. *Pancreas* 2011; 40(8):1264-70.

152. Kauffmann EF, Napoli N, Menonna F, et al. Robotic pancreatoduodenectomy with vascular resection. *Langenbecks Arch Surg* 2016; 401(8):1111-1122.

153. Kendrick ML, Sclabas GM. Major venous resection during total laparoscopic pancreaticoduodenectomy. *HPB (Oxford)* 2011; 13(7):454-8.

154. Khatkov IE, Izrailov RE, Khisamov AA, et al. Superior mesenteric-portal vein resection during laparoscopic pancreatoduodenectomy. *Surg Endosc* 2017; 31(3):1488-1495.

155. Marino MV, Giovinazzo F, Podda M, et al. Robotic-assisted pancreaticoduodenectomy with vascular resection. Description of the surgical technique and analysis of early outcomes. *Surg Oncol* 2020; 35:344-350.

156. Park H, Kang I, Kang CM. Laparoscopic pancreaticoduodenectomy with segmental resection of superior mesenteric vein-splenic vein-portal vein confluence in pancreatic head cancer: can it be a standard procedure? *Ann Hepatobiliary Pancreat Surg* 2018; 22(4):419-424.

157. Rosso E, Zimmitti G, Iannelli A, et al. The 'TRIANGLE Operation' by Laparoscopy: Radical Pancreaticoduodenectomy with Major Vascular Resection for Borderline Resectable Pancreatic Head Cancer. *Ann Surg Oncol* 2020; 27(5):1613-1614.

158. Shyr BU, Chen SC, Shyr YM, et al. Surgical, survival, and oncological outcomes after vascular resection in robotic and open pancreaticoduodenectomy. *Surg Endosc* 2020; 34(1):377-383.

159. Wang X, Cai Y, Jiang J, et al. Laparoscopic Pancreaticoduodenectomy: Outcomes and Experience of 550 Patients in a Single Institution. *Ann Surg Oncol* 2020; 27(11):4562-4573.

160. Wang X, Cai Y, Zhao W, et al. Laparoscopic pancreatoduodenectomy combined with portal-superior mesenteric vein resection and reconstruction with interposition graft: Case series. *Medicine (Baltimore)* 2019; 98(3):e14204.

161. Zureikat AH, Beane JD, Zenati MS, et al. 500 Minimally Invasive Robotic Pancreatoduodenectomies: One Decade of Optimizing Performance. *Ann Surg* 2021; 273(5):966-972.

162. Addeo P. Minimally invasive pancreaticoduodenectomy with venous resection: results of a systematic review. *Mini-invasive Surgery* 2020; 4:64.

163. Zhang YH, Zhang CW, Hu ZM, et al. Pancreatic cancer: Open or minimally invasive surgery? *World J Gastroenterol* 2016; 22(32):7301-10.

164. Wang S, Shi N, You L, et al. Minimally invasive surgical approach versus open procedure for pancreaticoduodenectomy: A systematic review and meta-analysis. *Medicine (Baltimore)* 2017; 96(50):e8619.

165. Cho A, Yamamoto H, Kainuma O, et al. Pure laparoscopic distal pancreatectomy with en bloc celiac axis resection. *J Laparoendosc Adv Surg Tech A* 2011; 21(10):957-9.

166. Greer J, Zureikat AH. Robotic distal pancreatectomy combined with celiac axis resection. *J Vis Surg* 2017; 3:145.

167. Kauffmann EF, Napoli N, Cacace C, et al. Resection or repair of large peripancreatic arteries during robotic pancreatectomy. *Updates Surg* 2020; 72(1):145-153.

168. Kim JH, Gonzalez-Heredia R, Daskalaki D, et al. Totally replaced right hepatic artery in pancreaticoduodenectomy: is this anatomical condition a contraindication to minimally invasive surgery? *HPB (Oxford)* 2016; 18(7):580-5.

169. Kim YS, Kim JS, Kim SH, et al. Laparoscopic radical distal pancreatosplenectomy with celiac axis excision following neoadjuvant chemotherapy for locally advanced pancreatic cancer. *Ann Hepatobiliary Pancreat Surg* 2022; 26(1):118-123.

170. Rao P, Schmidt CR, Boone BA. Robot Assisted Distal Pancreatectomy with Celiac Axis Resection (DP-CAR) for Pancreatic Cancer: Surgical Planning and Technique. *J Vis Exp* 2021(174).

171. Salehi O, Vega EA, Kutlu OC, et al. Combining Appleby with RAMPS - Laparoscopic Radical Antegrade Modular Pancreatosplenectomy with Celiac Trunk Resection. *J Gastrointest Surg* 2020; 24(11):2700-2701.

172. Ariyarathenam AV, Bunting D, Aroori S. Laparoscopic Distal Pancreatectomy Using the Modified Prolonged Prefiring Compression Technique Reduces Pancreatic Fistula. *J Laparoendosc Adv Surg Tech A* 2015; 25(10):821-5.

173. Nakamura M, Ueda J, Kohno H, et al. Prolonged peri-firing compression with a linear stapler prevents pancreatic fistula in laparoscopic distal pancreatectomy. *Surg Endosc* 2011; 25(3):867-71.

174. Hirashita T, Ohta M, Yada K, et al. Effect of pre-firing compression on the prevention of pancreatic fistula in distal pancreatectomy. *Am J Surg* 2018; 216(3):506-510.

175. Sepesi B, Moalem J, Galka E, et al. The influence of staple size on fistula formation following distal pancreatectomy. *J Gastrointest Surg* 2012; 16(2):267-74.

176. Kim H, Jang JY, Son D, et al. Optimal stapler cartridge selection according to the thickness of the pancreas in distal pancreatectomy. *Medicine (Baltimore)* 2016; 95(35):e4441.

177. Dokmak S, Ftériche FS, Meniconi RL, et al. Pancreatic fistula following laparoscopic distal pancreatectomy is probably unrelated to the stapler size but to the drainage modality and significantly decreased with a small suction drain. *Langenbecks Arch Surg* 2019; 404(2):203-212.

178. Kang MK, Kim H, Byun Y, et al. Optimal stapler cartridge selection to reduce post-operative pancreatic fistula according to the pancreatic characteristics in stapler closure distal pancreatectomy. *HPB (Oxford)* 2021; 23(4):633-640.

179. Carter TI, Fong ZV, Hyslop T, et al. A dual-institution randomized controlled trial of remnant closure after distal pancreatectomy: does the addition of a falciform patch and fibrin glue improve outcomes? *J Gastrointest Surg* 2013; 17(1):102-9.

180. Kawai M, Hirono S, Okada K, et al. Randomized Controlled Trial of Pancreaticojejunostomy versus Stapler Closure of the Pancreatic Stump During Distal Pancreatectomy to Reduce Pancreatic Fistula. *Ann Surg* 2016; 264(1):180-7.

181. Shubert CR, Ferrone CR, Fernandez-Del Castillo C, et al. A multicenter randomized controlled trial comparing pancreatic leaks after TissueLink versus SEAMGUARD after distal pancreatectomy (PLATS) NCT01051856. *J Surg Res* 2016; 206(1):32-40.

182. Landoni L, De Pastena M, Fontana M, et al. A randomized controlled trial of stapled versus ultrasonic transection in distal pancreatectomy. *Surg Endosc* 2021.

183. Pulvirenti A, Landoni L, Borin A, et al. Reinforced stapler versus ultrasonic dissector for pancreatic transection and stump closure for distal pancreatectomy: A propensity matched analysis. *Surgery* 2019; 166(3):271-276.

184. Ji W, Wang Y, Wang L, et al. Modified Hand-Sewn Closure With Retroperitoneal Tissue-covering Method Prevents Pancreatic Fistula in Laparoscopic Distal Pancreatectomy. *Surg Laparosc Endosc Percutan Tech* 2016; 26(5):e95-e99.

185. Park JS, Lee DH, Jang JY, et al. Use of TachoSil patches to prevent pancreatic leaks after distal pancreatectomy: a prospective, multicenter, randomized controlled study. *J Hepatobiliary Pancreat Sci* 2015.

186. Hamilton NA, Porembka MR, Johnston FM, et al. Mesh reinforcement of pancreatic transection decreases incidence of pancreatic occlusion failure for left pancreatectomy: a single-blinded, randomized controlled trial. *Ann Surg* 2012; 255(6):1037-42.

187. Merdrignac A, Garnier J, Dokmak S, et al. Effect of the Use of Reinforced Stapling on the Occurrence of Pancreatic Fistula After Distal Pancreatectomy: Results of the REPLAY (REinforcement of the Pancreas in distaL pAncreatectomY) Multicenter Randomized Clinical Trial. *Ann Surg* 2022; 276(5):769-775.

188. Kondo N, Uemura K, Nakagawa N, et al. A Multicenter, Randomized, Controlled Trial Comparing Reinforced Staplers with Bare Staplers During Distal Pancreatectomy (HiSCO-07 Trial). *Ann Surg Oncol* 2019; 26(5):1519-1527.

189. Wennerblom J, Ateeb Z, Jönsson C, et al. Reinforced versus standard stapler transection on postoperative pancreatic fistula in distal pancreatectomy: multicentre randomized clinical trial. *Br J Surg* 2021; 108(3):265-270.

190. Uranues S, Fingerhut A, Belyaev O, et al. Clinical Impact of Stump Closure Reinforced With Hemopatch on the Prevention of Clinically Relevant Pancreatic Fistula After Distal Pancreatectomy: A Multicenter Randomized Trial. *Annals of Surgery Open* 2021; 2(1):e033.

191. Pavlik Marangos I, Rosok BI, Kazaryan AM, et al. Effect of TachoSil patch in prevention of postoperative pancreatic fistula. *J Gastrointest Surg* 2011; 15(9):1625-9.

192. Aoki T, Mansour DA, Koizumi T, et al. Preventing clinically relevant pancreatic fistula with combination of linear stapling plus continuous suture of the stump in laparoscopic distal pancreatectomy. *BMC Surg* 2020; 20(1):223.

193. van Bodegraven EA, van Ramshorst TME, Balduzzi A, et al. Routine abdominal drainage after distal pancreatectomy: meta-analysis. *Br J Surg* 2022.

194. Van Buren G, 2nd, Bloomston M, Schmidt CR, et al. A Prospective Randomized Multicenter Trial of Distal Pancreatectomy With and Without Routine Intraperitoneal Drainage. *Ann Surg* 2017; 266(3):421-431.

195. Adham M, Chopin-Laly X, Lepilliez V, et al. Pancreatic resection: drain or no drain? *Surgery* 2013; 154(5):1069-77.

196. Correa-Gallego C, Brennan MF, Dʼangelica M, et al. Operative drainage following pancreatic resection: analysis of 1122 patients resected over 5 years at a single institution. *Ann Surg* 2013; 258(6):1051-8.

197. Kunstman JW, Starker LF, Healy JM, et al. Pancreaticoduodenectomy Can Be Performed Safely with Rare Employment of Surgical Drains. *Am Surg* 2017; 83(3):265-273.

198. Lim C, Dokmak S, Cauchy F, et al. Selective policy of no drain after pancreaticoduodenectomy is a valid option in patients at low risk of pancreatic fistula: a case-control analysis. *World J Surg* 2013; 37(5):1021-7.

199. McMillan MT, Malleo G, Bassi C, et al. Multicenter, Prospective Trial of Selective Drain Management for Pancreatoduodenectomy Using Risk Stratification. *Ann Surg* 2017; 265(6):1209-1218.

200. McMillan MT, Malleo G, Bassi C, et al. Drain Management after Pancreatoduodenectomy: Reappraisal of a Prospective Randomized Trial Using Risk Stratification. *J Am Coll Surg* 2015; 221(4):798-809.

201. Mehta VV, Fisher SB, Maithel SK, et al. Is it time to abandon routine operative drain use? A single institution assessment of 709 consecutive pancreaticoduodenectomies. *J Am Coll Surg* 2013; 216(4):635-42; discussion 642-4.

202. Van Buren G, 2nd, Bloomston M, Hughes SJ, et al. A randomized prospective multicenter trial of pancreaticoduodenectomy with and without routine intraperitoneal drainage. *Ann Surg* 2014; 259(4):605-12.

203. van der Wilt AA, Coolsen MM, de Hingh IH, et al. To drain or not to drain: a cumulative meta-analysis of the use of routine abdominal drains after pancreatic resection. *HPB (Oxford)* 2013; 15(5):337-44.

204. Witzigmann H, Diener MK, Kienkotter S, et al. No Need for Routine Drainage After Pancreatic Head Resection: The Dual-Center, Randomized, Controlled PANDRA Trial (ISRCTN04937707). *Ann Surg* 2016; 264(3):528-37.

205. Lyu Y, Cheng Y, Wang B, et al. Peritoneal drainage or no drainage after pancreaticoduodenectomy and/or distal pancreatectomy: a meta-analysis and systematic review. *Surg Endosc* 2020; 34(11):4991-5005.

206. Adam MA, Thomas S, Youngwirth L, et al. Defining a Hospital Volume Threshold for Minimally Invasive Pancreaticoduodenectomy in the United States. *JAMA Surg* 2017; 152(4):336-342.

207. Eguia E, Kuo PC, Sweigert PJ, et al. The laparoscopic approach to pancreatoduodenectomy is cost neutral in very high-volume centers. *Surgery* 2019; 166(6):1027-1032.

208. Tran TB, Dua MM, Worhunsky DJ, et al. The First Decade of Laparoscopic Pancreaticoduodenectomy in the United States: Costs and Outcomes Using the Nationwide Inpatient Sample. *Surg Endosc* 2016; 30(5):1778-83.

209. Conroy PC, Calthorpe L, Lin JA, et al. Determining Hospital Volume Threshold for Safety of Minimally Invasive Pancreaticoduodenectomy: A Contemporary Cutpoint Analysis. *Ann Surg Oncol* 2022; 29(3):1566-1574.

210. Mackay TM, Wellner UF, van Rijssen LB, et al. Variation in pancreatoduodenectomy as delivered in two national audits. *Br J Surg* 2019; 106(6):747-755.

211. Chan KS, Wang ZK, Syn N, et al. Learning curve of laparoscopic and robotic pancreas resections: a systematic review. *Surgery* 2021; 170(1):194-206.

212. Hua Y, Javed AA, Burkhart RA, et al. Preoperative risk factors for conversion and learning curve of minimally invasive distal pancreatectomy. *Surgery* 2017; 162(5):1040-1047.

213. Kim HS, Park JS, Yoon DS. True learning curve of laparoscopic spleen-preserving distal pancreatectomy with splenic vessel preservation. *Surg Endosc* 2019; 33(1):88-93.

214. Müller P, Kuemmerli C, Cizmic A, et al. Learning Curves in Open, Laparoscopic, and Robotic Pancreatic Surgery. A Systematic Review and Proposal of a Standardization. *Annals of Surgery Open* 2022; 1.

215. Lof S, Moekotte AL, Al-Sarireh B, et al. Multicentre observational cohort study of implementation and outcomes of laparoscopic distal pancreatectomy. *Br J Surg* 2019; 106(12):1657-1665.

216. Dokmak S, Aussilhou B, Ftériche FS, et al. The outcome of laparoscopic pancreatoduodenectomy is improved with patient selection and the learning curve. *Surg Endosc* 2022; 36(3):2070-2080.

217. Ke J, Liu Y, Liu F, et al. Application of Laparoscopic Pancreatoduodenectomy in Elderly Patients. *J Laparoendosc Adv Surg Tech A* 2020; 30(7):797-802.

218. Kim H, Choi SH, Jang JY, et al. Multicenter comparison of totally laparoscopic and totally robotic pancreaticoduodenectomy: Propensity score and learning curve-matching analyses. *J Hepatobiliary Pancreat Sci* 2022; 29(3):311-321.

219. Nagakawa Y, Nakamura Y, Honda G, et al. Learning curve and surgical factors influencing the surgical outcomes during the initial experience with laparoscopic pancreaticoduodenectomy. *J Hepatobiliary Pancreat Sci* 2018; 25(11):498-507.

220. Wang M, Meng L, Cai Y, et al. Learning Curve for Laparoscopic Pancreaticoduodenectomy: a CUSUM Analysis. *J Gastrointest Surg* 2016; 20(5):924-35.

221. Shakir M, Boone BA, Polanco PM, et al. The learning curve for robotic distal pancreatectomy: an analysis of outcomes of the first 100 consecutive cases at a high-volume pancreatic centre. *HPB (Oxford)* 2015; 17(7):580-6.

222. Shyr BU, Chen SC, Shyr YM, et al. Learning curves for robotic pancreatic surgery-from distal pancreatectomy to pancreaticoduodenectomy. *Medicine (Baltimore)* 2018; 97(45):e13000.

223. Boone BA, Zenati M, Hogg ME, et al. Assessment of quality outcomes for robotic pancreaticoduodenectomy: identification of the learning curve. *JAMA Surg* 2015; 150(5):416-22.

224. Napoli N, Kauffmann EF, Palmeri M, et al. The Learning Curve in Robotic Pancreaticoduodenectomy. *Dig Surg* 2016; 33(4):299-307.

225. Shi Y, Wang W, Qiu W, et al. Learning Curve From 450 Cases of Robot-Assisted Pancreaticoduocectomy in a High-Volume Pancreatic Center: Optimization of Operative Procedure and a Retrospective Study. *Ann Surg* 2021; 274(6):e1277-e1283.

226. Zwart MJW, Nota CLM, de Rooij T, et al. Outcomes of a Multicenter Training Program in Robotic Pancreatoduodenectomy (LAELAPS-3). *Ann Surg* 2021.

227. Al Abbas AI, Wang C, Hamad AB, et al. Mentorship and formal robotic proficiency skills curriculum improve subsequent generations' learning curve for the robotic distal pancreatectomy. *HPB (Oxford)* 2021; 23(12):1849-1855.

228. Hogg ME, Besselink MG, Clavien PA, et al. Training in Minimally Invasive Pancreatic Resections: a paradigm shift away from "See one, Do one, Teach one". *HPB (Oxford)* 2017; 19(3):234-245.

229. Hogg ME, Tam V, Zenati M, et al. Mastery-Based Virtual Reality Robotic Simulation Curriculum: The First Step Toward Operative Robotic Proficiency. *J Surg Educ* 2017; 74(3):477-485.

230. Jones LR, Zwart MJW, Molenaar IQ, et al. Robotic Pancreatoduodenectomy: Patient Selection, Volume Criteria, and Training Programs. *Scand J Surg* 2020; 109(1):29-33.

231. Klompmaker S, van der Vliet WJ, Thoolen SJ, et al. Procedure-specific Training for Robot-assisted Distal Pancreatectomy. *Ann Surg* 2021; 274(1):e18-e27.

232. Knab LM, Zenati MS, Khodakov A, et al. Correction to: Evolution of a Novel Robotic Training Curriculum in a Complex General Surgical Oncology Fellowship. *Ann Surg Oncol* 2019; 26(Suppl 3):879.

233. Knab LM, Zureikat AH, Zeh HJ, 3rd, et al. Towards standardized robotic surgery in gastrointestinal oncology. *Langenbecks Arch Surg* 2017; 402(7):1003-1014.

234. Nota CL, Zwart MJ, Fong Y, et al. Developing a robotic pancreas program: the Dutch experience. *J Vis Surg* 2017; 3:106.

235. Rice MK, Hodges JC, Bellon J, et al. Association of Mentorship and a Formal Robotic Proficiency Skills Curriculum With Subsequent Generations' Learning Curve and Safety for Robotic Pancreaticoduodenectomy. *JAMA Surg* 2020; 155(7):607-615.

236. Ryoo DY, Eskander MF, Hamad A, et al. Mitigation of the Robotic Pancreaticoduodenectomy Learning Curve through comprehensive training. *HPB (Oxford)* 2021; 23(10):1550-1556.

237. Schmidt CR, Harris BR, Musgrove KA, et al. Formal robotic training diminishes the learning curve for robotic pancreatoduodenectomy: Implications for new programs in complex robotic surgery. *J Surg Oncol* 2021; 123(2):375-380.

238. Tam V, Zenati M, Novak S, et al. Robotic Pancreatoduodenectomy Biotissue Curriculum has Validity and Improves Technical Performance for Surgical Oncology Fellows. *J Surg Educ* 2017; 74(6):1057-1065.

239. de Rooij T, van Hilst J, Boerma D, et al. Impact of a Nationwide Training Program in Minimally Invasive Distal Pancreatectomy (LAELAPS). *Ann Surg* 2016; 264(5):754-762.

240. de Rooij T, van Hilst J, Topal B, et al. Outcomes of a Multicenter Training Program in Laparoscopic Pancreatoduodenectomy (LAELAPS-2). *Ann Surg* 2019; 269(2):344-350.

241. Mackay TM, Gleeson EM, Wellner UF, et al. Transatlantic registries of pancreatic surgery in the United States of America, Germany, the Netherlands, and Sweden: Comparing design, variables, patients, treatment strategies, and outcomes. *Surgery* 2021; 169(2):396-402.

242. Mintziras I, Keck T, Werner J, et al. Indications for resection and perioperative outcomes of surgery for pancreatic neuroendocrine neoplasms in Germany: an analysis of the prospective DGAV StuDoQ|Pancreas registry. *Surg Today* 2019; 49(12):1013-1021.

243. van der Heijde N, Vissers FL, Boggi U, et al. Designing the European registry on minimally invasive pancreatic surgery: a pan-European survey. *HPB (Oxford)* 2021; 23(4):566-574.

244. Zerbi A, Capretti G, Napoli N, et al. The Italian National Registry for minimally invasive pancreatic surgery: an initiative of the Italian Group of Minimally Invasive Pancreas Surgery (IGoMIPS). *Updates Surg* 2020; 72(2):379-385.

245. Eguia E, Kuo PC, Sweigert P, et al. The laparoscopic approach to distal pancreatectomy is a value-added proposition for patients undergoing care in moderate-volume and high-volume centers. *Surgery* 2019; 166(2):166-171.

246. Fisher AV, Fernandes-Taylor S, Schumacher JR, et al. Analysis of 90-day cost for open versus minimally invasive distal pancreatectomy. *HPB (Oxford)* 2019; 21(1):60-66.

247. Gerber MH, Delitto D, Crippen CJ, et al. Analysis of the Cost Effectiveness of Laparoscopic Pancreatoduodenectomy. *J Gastrointest Surg* 2017; 21(9):1404-1410.

248. Joechle K, Conrad C. Cost-effectiveness of minimally invasive pancreatic resection. *J Hepatobiliary Pancreat Sci* 2018; 25(6):291-298.

249. Limongelli P, Vitiello C, Belli A, et al. Costs of laparoscopic and open liver and pancreatic resection: a systematic review. *World J Gastroenterol* 2014; 20(46):17595-602.

250. Partelli S, Ricci C, Cinelli L, et al. Evaluation of cost-effectiveness among open, laparoscopic and robotic distal pancreatectomy: A systematic review and meta-analysis. *Am J Surg* 2021; 222(3):513-520.

251. Rutz DR, Squires MH, Maithel SK, et al. Cost comparison analysis of open versus laparoscopic distal pancreatectomy. *HPB (Oxford)* 2014; 16(10):907-14.

252. van Hilst J, Strating EA, de Rooij T, et al. Costs and quality of life in a randomized trial comparing minimally invasive and open distal pancreatectomy (LEOPARD trial). *Br J Surg* 2019; 106(7):910-921.

253. Cunningham KE, Zenati MS, Petrie JR, et al. A policy of omitting an intensive care unit stay after robotic pancreaticoduodenectomy is safe and cost-effective. *J Surg Res* 2016; 204(1):8-14.

254. Magge DR, Zenati MS, Hamad A, et al. Comprehensive comparative analysis of cost-effectiveness and perioperative outcomes between open, laparoscopic, and robotic distal pancreatectomy. *Hpb* 2018; 20:1172-1180.

**SUPPLEMENTARY APPENDIX**

**Appendix S1.** Steering Committee

| **Name** | **Country** |
| --- | --- |
| Marc Besselink - *Chair* | The Netherlands |
| Ugo Boggi - *Co chair* | Italy |
| Safi Dokmak - *Co chair* | France |
| Tobias Keck - *Co chair* | Germany |
| Igor Khatkov*- Co chair* | Russia |
| Bjørn Edwin - *Co chair* | Norway |

**Appendix S2.** European Expert Committee

| **Name** | **Country** |
| --- | --- |
| Bas Groot Koerkamp | The Netherlands |
| Misha Luyer | The Netherlands |
| Alessandro Esposito | Italy |
| Giovanni Ferrari | Italy |
| Alessandro Zerbi | Italy |
| Cristiano Huscher | Italy |
| Bergthor Björnsson | Sweden |
| Andrew Gumbs | France |
| Olivier Saint-Marc | France |
| Tullio Piardi | France |
| Mikhail Efanov | Russia |
| Steve White | United Kingdom |
| Jawad Ahmad | United Kingdom |
| Krishna Menon | United Kingdom |
| Benedetto Ielpo | Spain |
| Francisco Espin-Alvarez | Spain |
| Mathieu D'Hondt | Belgium |
| Fatih Can | Turkey |
| Hani Alsaati | Bahrein |
| Arpad Ivanecz | Slovenia |
| Juan Azagra | Luxembourg |

**Appendix S3.** International Expert Committee

| **Name** | **Country** |
| --- | --- |
| Adnan Alseidi | United States of America |
| Melissa Hogg | United States of America |
| Masafumi Nakamura | Japan |
| Rong Liu | China |
| Jin-Young Jang | Korea |
| Yoo-Seok Yoon | Korea |

**Appendix S4.** Validation Committee

| **Name** | **Country** |
| --- | --- |
| Horacio Asbun - *Chair* | United States of America |
| Claudio Bassi | Italy |
| Massimo Falconi | Italy |
| Isabella Frigerio | Italy |
| Piero Rivizzigno – *Patient representative* | Italy |
| Clarissa Ferrari – *Methodologist* | Italy |
| Andrew Cook - *Methodologist* | United Kingdom |
| Ajith Siriwardena | United Kingdom |
| John Primrose | United Kingdom |
| Giuseppe Kito Fusai | United Kingdom |
| Paul Over - *Patient representative* | United Kingdom |
| Omar Saleh - *Patient representative* | United Kingdom |
| Christos Dervenis | Greece |
| Alejandro Serrablo | Spain |
| Michelle De Oliveira | Swiss |
| Frederik Berrevoet | Belgium |
| Kevin Conlon | Ireland |
| Alain Sauvanet | France |
| Sameer Smadi | Jordan |
| Felipe Coimbra | Brazil |
| Antonio Pinna | Florida |
| Carlos Chan | Mexico |

**Appendix S5.** Jury Committee

| **Name** | **Country** |
| --- | --- |
| Roberto Salvia - *Chair* | Italy |
| Sergio Alfieri | Italy |
| Stefano Berti | Italy |
| Giovanni Butturini | Italy |
| Fabrizio Di Benedetto | Italy |
| Giuseppe Maria Ettorre | Italy |
| Felice Giuliante | Italy |
| Elio Jovine | Italy |
| Nazario Portolani | Italy |
| Riccardo Memeo | Italy |
| Andrea Ruzzenente | Italy |

**Appendix S6.** Research Committee

| **Name** | **Country** |
| --- | --- |
| Tess van Ramshorst - *Chair* | The Netherlands |
| Jony van Hilst | The Netherlands |
| Nine de Graaf | The Netherlands |
| Anouk Emmen | The Netherlands |
| Maurice Zwart | The Netherlands |
| Leia Jones | The Netherlands |
| Bas Uijterwijk | The Netherlands |
| Ward van Bodegraven | The Netherlands |
| Marco Ramera | Italy |
| Alessandra Pulvirenti | Italy |
| Ali Badran | Italy |
| Elisa Bannone | Italy |
| Alessandro Giani | Italy |
| Pietro Zampedri | Italy |
| Mushegh Sahakyan | Norway |
| Magomet Baichorov | Russia |
| Giovanni Battista Levi Sandri | Italy |
| Niki Rashidian | Belgium |

**Appendix S7.** Grading of the quality of evidence.

| **A. High quality of evidence** | Consistent evidence from well performed randomized, controlled trials or overwhelming evidence of some other form. Further research is unlikely to change our confidence in the estimate of benefit and risk. |
| --- | --- |
| **B. Moderate quality of evidence** | Evidence from randomized, controlled trials with important limitations (inconsistent results, methodologic flaws, indirect or imprecise), or very strong evidence of some other form. Further research (if performed) is likely to have an impact on our confidence in the estimate of benefit and risk and may change the estimate. |
| **C. Low quality of evidence** | Evidence from observational studies, unsystematic clinical experience, or from randomized, controlled trials with serious flaws. Any estimate of effect is uncertain. |

Adapted with permission from: Grading Tutorial. In: UpToDate, Post TW (Ed), UpToDate, Waltham, MA. (Accessed on Nov 1st, 2022.) Copyright © 2022 UpToDate, Inc. For more information, visit [www.uptodate.com](http://www.uptodate.com)

**Appendix S8.** GRADE recommendations.

| **Quality of evidence** | **1. Strong recommendation** | **2. Weak recommendation** |
| --- | --- | --- |
| A. High quality of evidence | 1A. Benefits clearly outweigh risk and burdens, or vice versa | 2A. Benefits closely balanced with risks and burdens |
| B. Moderate quality of evidence | 1B. Benefits clearly outweigh risk and burdens, or vice versa | 2B. Benefits closely balanced with risks and burdens, some uncertainty in the estimates of benefits, risks, and burdens |
| C. Low quality of evidence | 1C. Benefits appear to outweigh risk and burdens, or vice versa | 2C. Uncertainty in the estimates of benefits, risks, and burdens; benefits may be closely balanced with risks and burdens |

Adapted, with permission, from: Grading Tutorial. In: UpToDate, Post TW (Ed), UpToDate, Waltham, MA. (Accessed on Nov 1st, 2022.) Copyright © 2022 UpToDate, Inc. For more information, visit [www.uptodate.com](http://www.uptodate.com)

**Appendix S9.** Endorsement letter International Hepato-Pancreato-Biliary Association

**
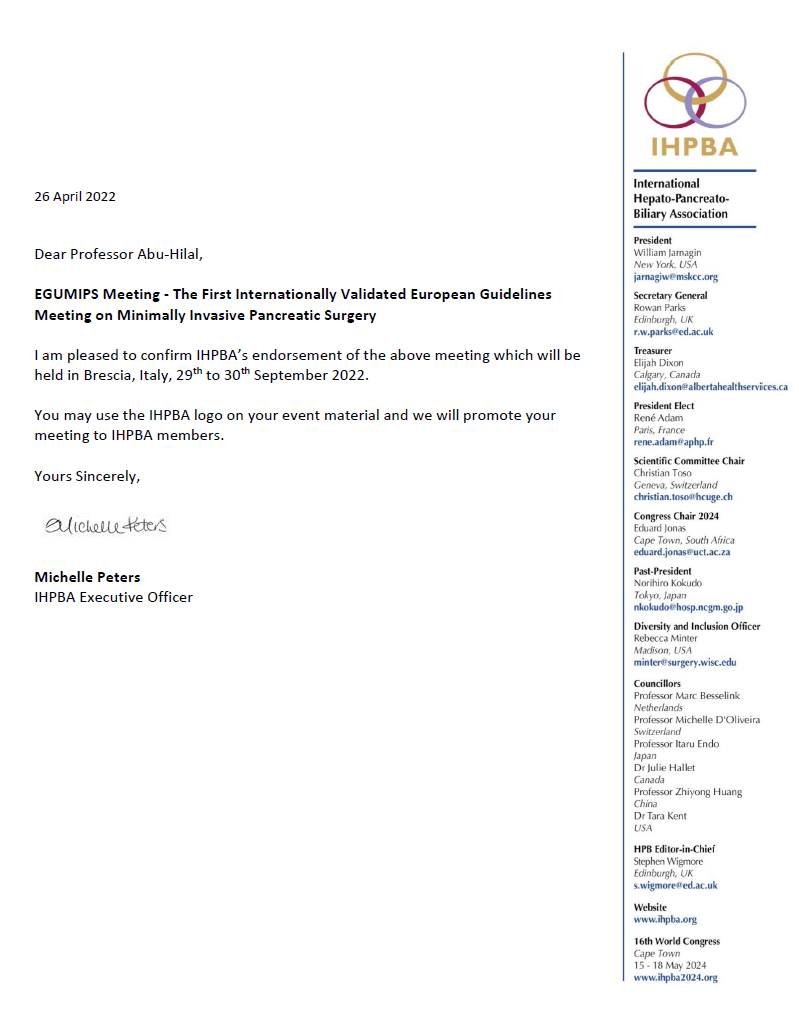
**

**Appendix S10.** Endorsement letter European-African Hepato-Pancreato-Biliary Association

**
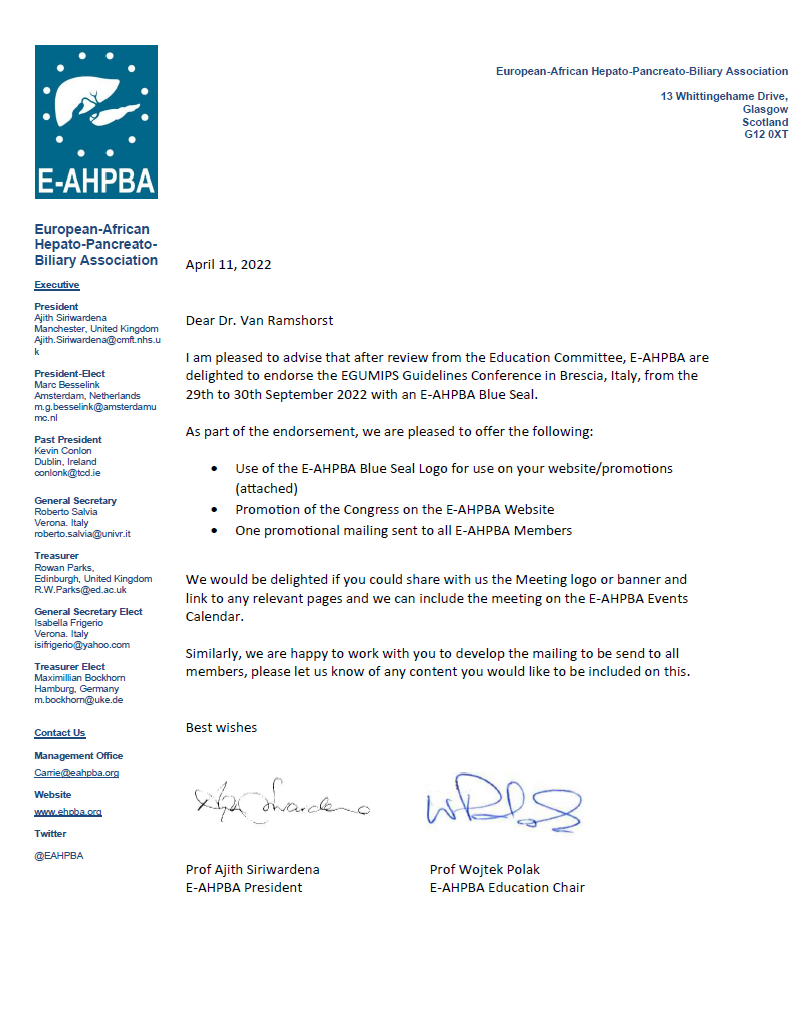
**

**Appendix S11.** Endorsement letter Society of American Gastrointestinal and Endoscopic Surgeons


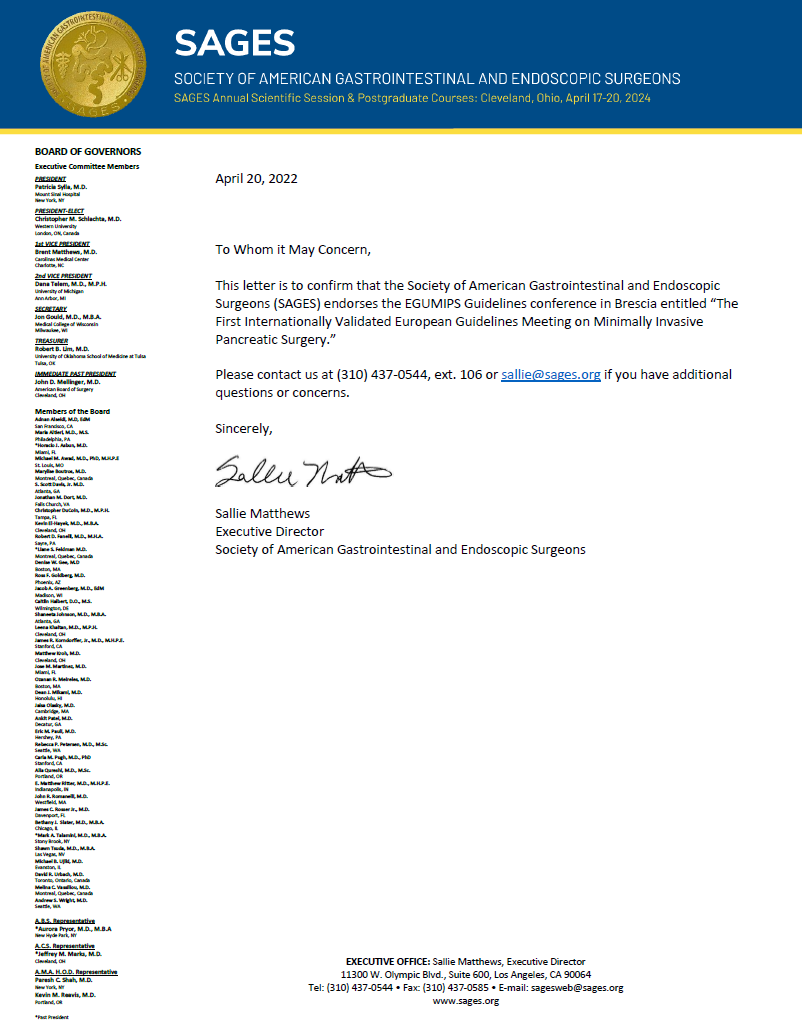


**Appendix S12.** Endorsement letter Società Italiana di Chirurgia

**
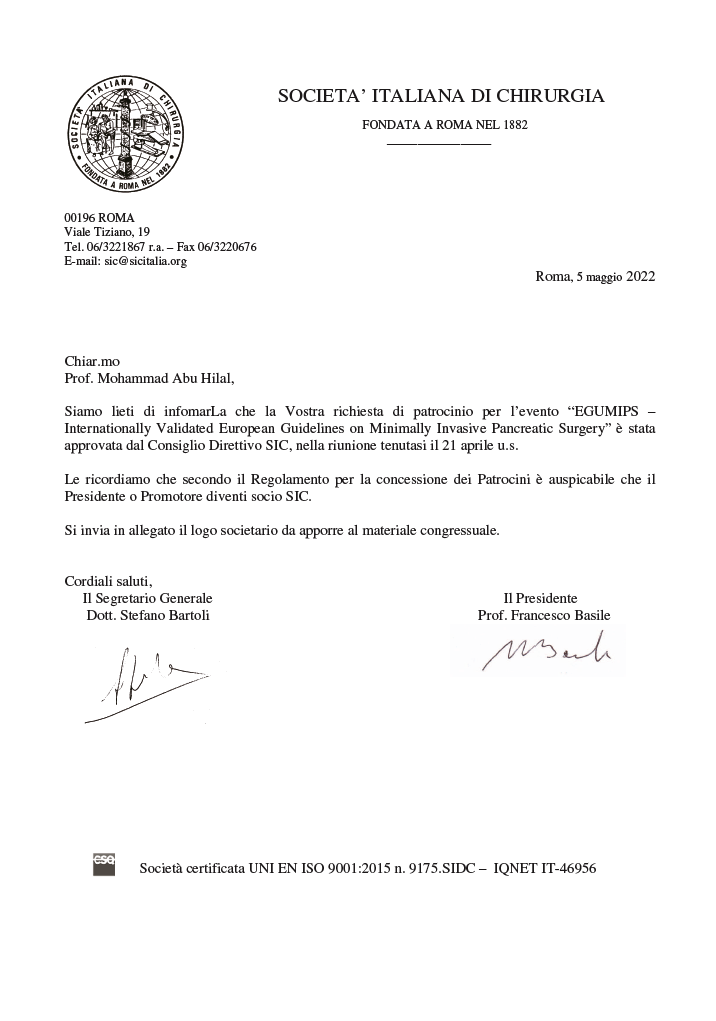
**

**Appendix S13.** Endorsement letter Associazione Italiana per lo Studio del Pancreas

**
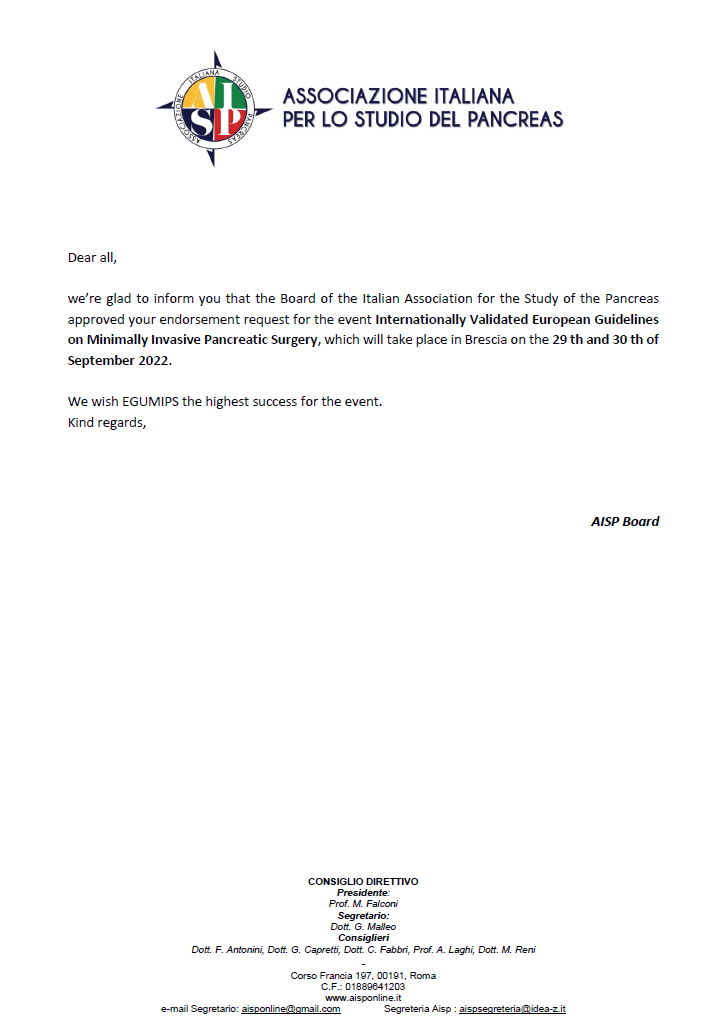
**

**Appendix S14.** Endorsement letter Women in Surgery

**
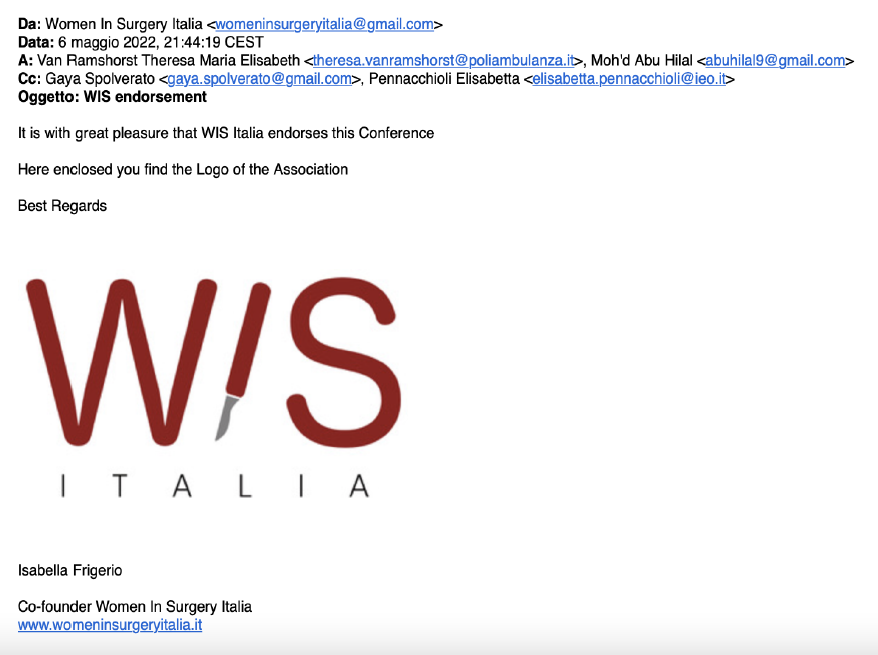
**

**SUPPLEMENTARY FIGURES**

**FIGURE S1.** PRISMA diagram of the systematic literature review of all domains.


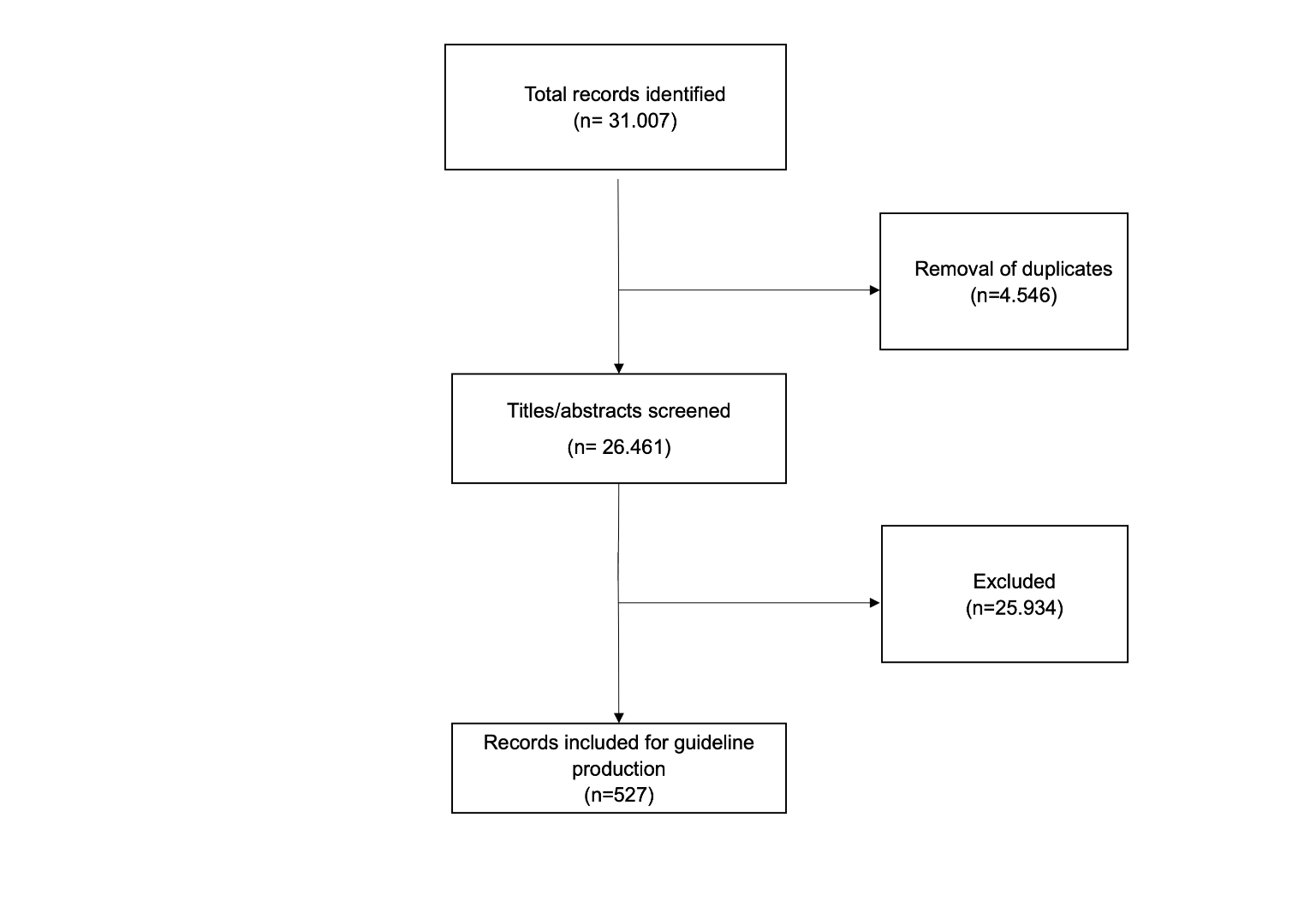


**FIGURE S2.** Flow chart of the recommendations process.
